# Structures of dynamic interactors at native proteasomes by PhIX-MS and cryo-electron microscopy

**DOI:** 10.1016/j.molcel.2026.06.032

**Published:** 2026-07-21

**Authors:** Kitaik Lee, Hitendra Negi, Xiang Chen, Katerina Atallah-Yunes, Sophia Truslow, Rithik E. Castelino, Mary R. Guest, Anthony M. Ciancone, Xiuxiu Lu, Sergey G. Tarasov, Raj Chari, Kylie J. Walters, Francis J. O’Reilly

**Affiliations:** 1 Structural System Biology Section, Center for Structural Biology, Center for Cancer Research, National Cancer Institute (NCI), National Institutes of Health, Frederick, MD 21702-1201, USA; 2 Protein Processing Section, Center for Structural Biology, Center for Cancer Research, National Cancer Institute (NCI), National Institutes of Health, Frederick, MD 21702-1201, USA; 3 Genome Modification Core, Laboratory Animal Sciences Program, Frederick National Laboratory for Cancer Research, Frederick, MD 21702-1201, USA; 4 Biophysics Resource, Center for Structural Biology, Center for Cancer Research, National Cancer Institute (NCI), National Institutes of Health, Frederick, MD 21702-1201, USA; 5 Center of Excellence for Data Driven Discovery, Department of Structural Biology, St Jude Children’s Research Hospital, Memphis, TN 38105, USA; 6 These authors contributed equally; 7 Senior author; 8 Lead contact

## Abstract

Molecular machines rely on dynamic, low-affinity interactions to perform their functional roles. We developed PhIX-MS (photo-induced *in situ* crosslinking-mass spectrometry), a structural proteomics workflow to capture topological information for such transient interactions in cells by UV-activated crosslinking. Applying PhIX-MS with cryo-electron microscopy (cryo-EM) to proteasomes, we mapped the redox sensor TXNL1 at the proteasome regulatory particle (RP), including its dynamic thioredoxin-like domain near RPN2/PSMD1 and RPN13/ADRM1, where it is ideal for reducing substrates prior to proteolysis. RPs without the proteolytic core particle (CP) were structurally resolved while bound to TXNL1 and/or the chaperone PSMD5/S5b, which inserts its C terminus into the ATPase pore, causing extensive structural rearrangements. Additionally, PhIX-MS and AlphaFold identified the ubiquitin ligase UBE3C/Hul5 at RPN2, RPN3, and a dynamic RPN10 region, tethering UBE3C above the substrate entry channel. Our integrative approach enables the localization of native, low-affinity protein interactions and is broadly applicable to dynamic macromolecular assemblies.

## In brief

Lee et al. developed PhIX-MS, a UV-activated crosslinking structural proteomics workflow that traps transient interactions in cells to provide residue-to-residue distance information. These data are integrated with AlphaFold and/or cryo-EM to solve structures of the 26S or 19S proteasome bound to transient interactors, notably UBE3C, PSMD5, and TXNL1.

## Graphical Abstract

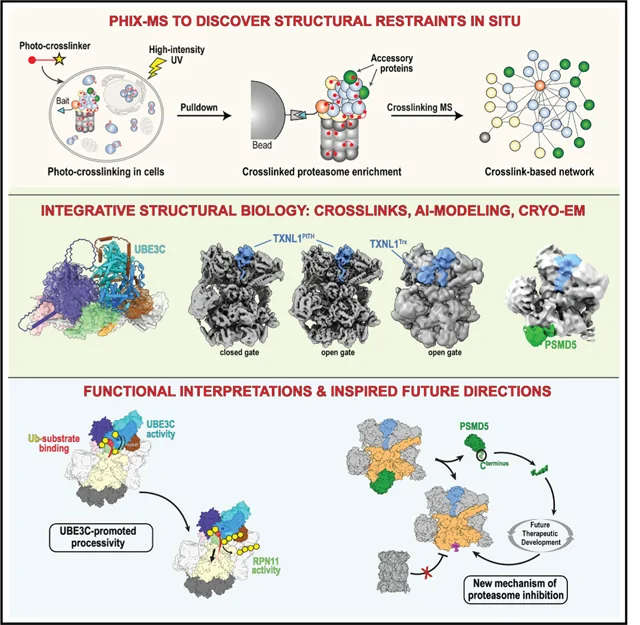

## INTRODUCTION

The ubiquitin-proteasome system is the primary mechanism for targeted protein degradation, removing damaged, misfolded, and/or regulatory proteins that require tightly controlled lifespans.^1^ Proteolysis occurs within a hollow, cylindrical 20S core particle (CP) formed by four heptameric rings: two interior β-rings (PSMB1–7) and two outer α-rings (PSMA1–7). CP inhibitors are used as the standard treatment for hematological cancers.^2^ Proteasomal degradation is highly regulated and coordinated with ~1,000 enzymes involved in the posttranslational modification of substrates with ubiquitin.^3,4^

To degrade ubiquitinated proteins, the CP is capped at one end (26S) or both ends (30S) by a 19S regulatory particle (RP),^5,6^ which orchestrates substrate recognition, deubiquitination, unfolding, and translocation into the CP and can be biochemically separated into base and lid subcomplexes.^7,8^ Within the base are ubiquitin receptors (RPN1, RPN10, and RPN13) and a dynamic heterohexameric AAA+ ATPase ring (RPT1–6) that unfolds and translocates substrates into the CP.^1^ Lid proteins fan out along the base and include the essential deubiquitinase RPN11/PSMD14.^9^ The proteasome binds transient regulators, including ubiquitination machinery,^10–14^ such as the ubiquitin HECT (Homologous to the E6AP Carboxyl Terminus) E3 ligase paralogs UBE3A/E6AP and UBE3C/HUL5.

30S/26S proteasomes can specialize across tissue and cell types by incorporating proteins expressed only in a specific tissue.^15^ Alternative proteasome regulators can replace the RP to confer specialized functions in response to cellular stress or immune signaling.^16,17^ Notably, bacterial infection was found to trigger changes in proteasome composition and function by recruiting PSME3 to the CP to generate antimicrobial peptides.^18^ However, these interactions are notoriously dynamic, resulting in technical challenges for capturing the architecture of these varied proteasome complexes.

Mass spectrometry (MS) has been foundational to defining the dynamic proteasome interactome, through tagged proteasome purifications,^13,19,20^ the use of crosslinking approaches,^21–23^ differential ultracentrifugation,^24^ and proximity labeling.^25^ Many of these approaches lack topological information for the discovered protein interactions, so they cannot readily direct further structure/function experiments. We therefore developed PhIX-MS (photo-induced *in situ* crosslinking MS), a targeted structural proteomics strategy based on rapid *in situ* photo-crosslinking to capture dynamic, native protein interactions with high temporal fidelity. Photo-activated cross-linkers, when activated by UV light, react for only nanoseconds and therefore provide less background than homobifunctional NHS (N-hydroxysuccinimide)-ester crosslinkers,^26,27^ limiting crosslinking to true interfaces.^28–30^ These crosslinks serve as distance restraints to guide AI-assisted structure modeling and cryo-electron microscopy (cryo-EM) analyses. Applying PhIX-MS to HCT116 (human colon tumor 116) cells revealed the binding sites of several proteasome regulators, including UBE3C/HUL5.^10,14,31–33^ PhIX-MS combined with cryo-EM also resolved the structure of the proteasome chaperone PSMD5/S5b/HSM3 bound to RP, revealing how it promotes RP assembly^34–36^ and restricts 26S/30S proteasome formation.^37,38^ Together, these data provide a structural blueprint of the human proteasome interactome and establish a framework for studying dynamic protein complexes in their native environment.

## RESULTS

### PhIX-MS: A strategy to trap the proteasome interactome

**Figure 1.**
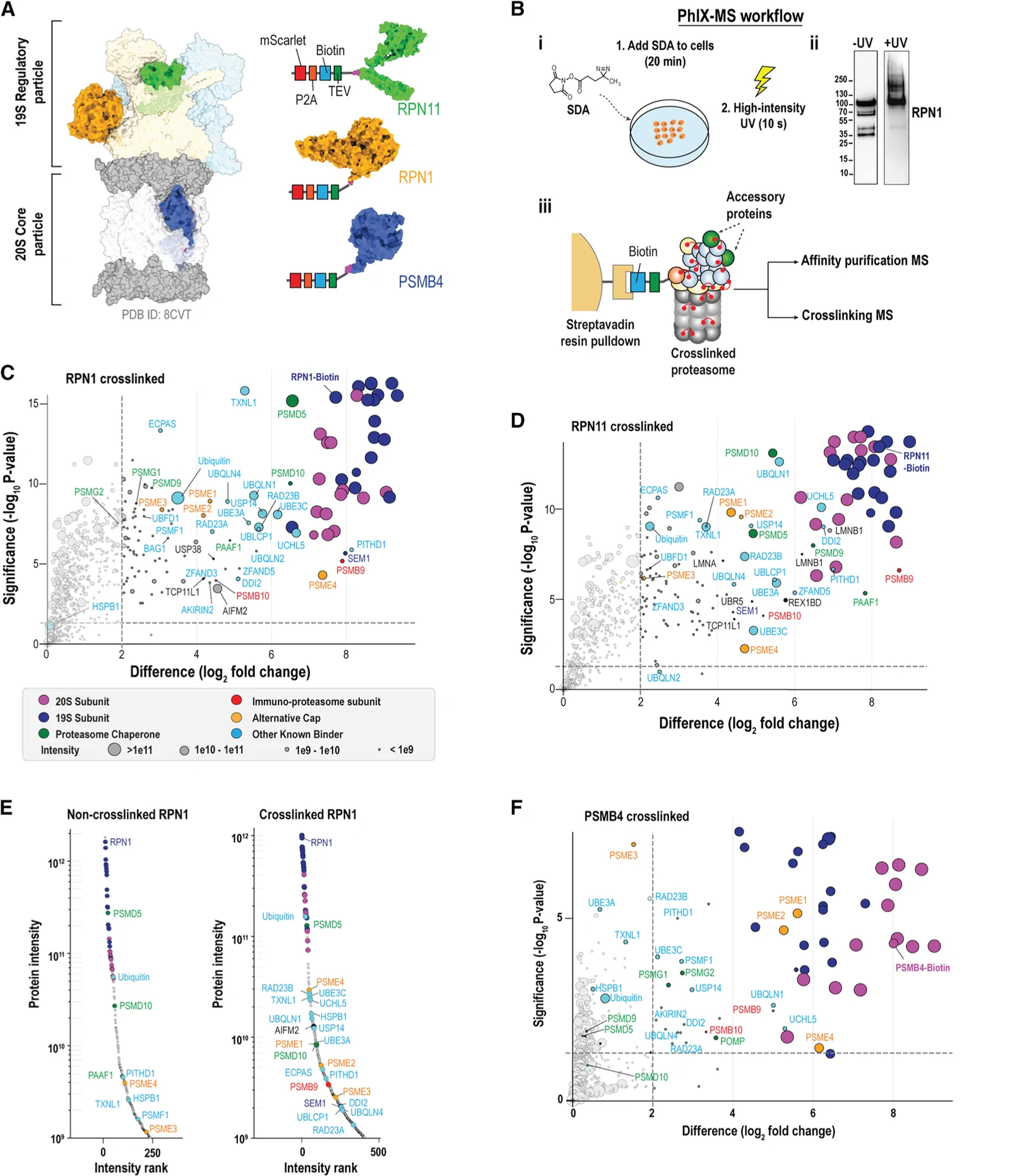
PhIX-MS approach (A) Surface rendering of the 26S proteasome highlighting subunits engineered with endogenous biotin-TEV purification tags. (B) PhIX-MS workflow. (i) Cells are treated with SDA and UV-irradiated to stabilize native interactions. (ii) Immunoprobing of lysates for RPN1 indicates higher-molecular-weight protein complexes following crosslinking of cells. (iii) Crosslinked proteasomes are pulled down on streptavidin beads. On-bead digestion is used to identify enriched proteins by AP-MS. Proteasomes are eluted with TEV for analysis by crosslinking MS. (C, D, and F) AP-MS enrichment for crosslinked pull-downs of RPN1, RPN11, and PSMB4. The horizontal dashed line denotes statistical significance (Benjamini Hochberg [BH]-adjusted *p* < 0.05), and the vertical dashed lines mark log_2_ fold changes in abundance between tagged and wild-type pull-downs. (E) Rank intensity plots showing protein abundance (intensity from the MSFragger search) from biotin-tagged RPN1 pull-downs, from crosslinked and non-crosslinked cells.

To isolate proteasomes for MS analyses, we used HCT116 cells edited to include a biotinylated tag-TEV (tobacco etch virus) purification handle fused to endogenously expressed proteins at different positions within the proteasome complex, including the RP lid (RPN1), RP base (RPN11), or CP (PSMB4). RPN1 and RPN11 tagging had previously been used for proteasome purification,^13,39,40^ and we used our HCT116 RPN1-tagged cell line^40^ and generated two additional HCT116 cell lines (Data S1) to fuse the biotin-TEV handle to RPN11 or PSMB4/β4 (Figures 1A and S1A). The tagged proteasomes were readily purified by streptavidin beads with the expected SDS-PAGE profile (Figure S1B) and were proteolytically active (Figure S1C). RPN11 tagging indicated equivalent activity for 26S and 30S complexes, whereas RPN1 or PSMB4 tagging predominantly yielded active 30S or CP, respectively.

We next developed PhIX-MS (Figures 1B and S1D) to trap weakly bound proteasome interactors *in situ*. Before lysis, we treated cells with the cell-permeable, heterobifunctional cross-linker succinimidyl 4,4′-azipentanoate (SDA), which first reacts with primary amines (including lysine and N termini of proteins) and then, upon UV activation, forms nanosecond-lived intermediates that react with nearby residues (Cα to Cα, < 27Å).^28,29^ Following purification by the biotin handle, label-free quantitative MS (affinity purified-MS [AP-MS]) was used to identify crosslinked proteins.

By comparing pull-downs without or with crosslinking (Figures S2A and S2B for RPN1; Figures S2C and S2D for RPN11), we found this approach to significantly enrich known proteasome interactors (Figures 1C and 1D; Data S2). The crosslinking maintained the fidelity of native proteasome interactions, as shown by the enrichment of very few typical AP-MS contaminants and abundant cellular proteins.^41^ Even with the very mild crosslinking performed here, the abundance of the proteasome-interacting proteins in these RP pull-downs increased dramatically compared with non-crosslinked pull-downs (Figures 1E, S2E, and S2F). Ubiquitin is particularly increased following crosslinking with the purification handle on RPN1, perhaps because it itself is a ubiquitin-binding substrate receptor,^42^ and correspondingly, so too are proteins that interact with proteasomes in a ubiquitin-dependent manner, including ubiquitin shuttle factors^43^ RAD23B, RAD23A, UBQLN1, UBQLN4, DDI2, the deubiquitinase USP14,^44^ and the ubiquitin E3 ligase UBE3C^44^ (Figure 1E).

UBE3C is undetectable without crosslinking but represents 4.6% and 3.0% of the RP label-free quantification (LFQ) intensity in the RPN11 and RPN1 pull-downs (Figures 1E, S2E, and S2F), with significant enrichment compared with the control (Figures S2A–S2D). TXNL1 was similarly crosslinking-dependent, increasing 39- and 13-fold in RPN1 (Figure S2E) and RPN11 (Figure S2F) pull-downs, respectively. Strikingly, alternative regulatory caps, including PSME4 (PA200) and PSME1/2/3 (PA28α/β/γ), were also strongly enriched in crosslinked samples (Figures S2E and S2F). PSME4 was present at 30% (RPN1, Figure S2G, top) or 17% (RPN11) of the CP LFQ intensity identified in the crosslinked pull-downs (Figure S2G, top). This enrichment suggests that the abundance of hybrid proteasome complexes, such as 19S-20S-PSME4 and 19S-20S-PA28, is higher than expected, perhaps because these complexes are labile and earlier studies lacked *in situ* stabilization.^20^

The PSMB4 pull-downs enriched the CP chaperones PSMG1, PSMG2, and POMP, which were not pulled down by tagged RPN11 (Figures 1D, 1F, S2D, S2F, and S2H), and only PSMG1 was above significance for tagged RPN1 (Figure 1C). Conversely, the RP chaperones PSMD9/p27, PSMD5/S5B, PSMD10/p28, and PAAF were not enriched in the CP pull-downs (Figures 1F, S2I, and S2J). Alternate CP caps PSME1, PSME2, and PSME4 were strongly enriched in the PSMB4 pull-downs, which did not suggest any further alternate caps beyond those previously reported (Figures 1F and S2G, bottom).

Altogether, this photo-crosslinking approach traps transient interactors and chaperones at proteasome complexes with minimal non-specific background and reveals a greater-than-expected abundance of hybrid proteasomes.

### PhIX-MS-derived distance restraints filter AlphaFold predictions to identify novel interfaces

We identified crosslinked residue pairs in our pull-downs, with 2,236 inter-protein crosslinks describing 276 protein-protein interactions (PPIs) for the combined RPN1 and RPN11 datasets (Data S3). Between canonical proteasome subunits, we identified 562 and 1,697 crosslinks from the RPN1-purified and RPN11-purified complexes, respectively (Figure S3A). The majority of these intra-proteasome crosslinks are localized within the RP (Data S3), as expected from the location of the affinity tags (Figure 1A). Mapping these restraints onto individual proteasome structures revealed clusters of overlength links (>27 Å Cα-Cα), consistent with the conformational heterogeneity of the RP (Figure S3B).^1^ However, 93.5% of the crosslink distances were satisfied on at least one of the 20 available proteasome structures (Figure S3C; Data S4). Different clusters of crosslinks representing different conformational states are apparent by inspection of the position of RPN1 relative to the ATPase ring in S_B_^USP14^ (PDB: 7W3I) and E_A1_^UBL^ (PDB: 7W37) (Figure S3D). We therefore concluded that the purified proteasome samples previously studied closely recapitulate the native in-cell conformational ensembles. One area where the crosslinking data suggest a previously unobserved configuration is between RPN8 and RPN9 (Figure S3E).

**Figure 2.**
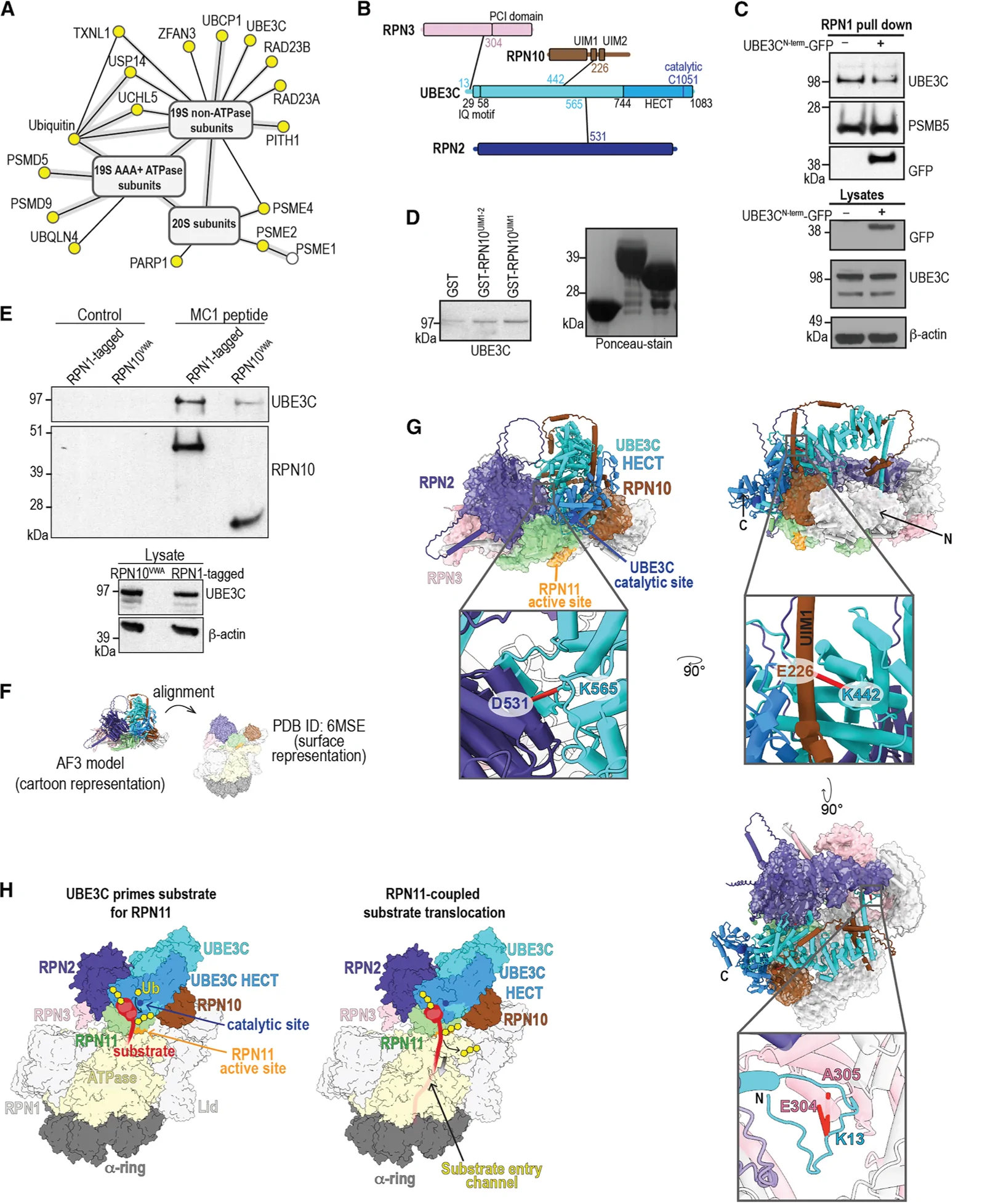
PhIX-MS-derived crosslinks and structural modeling localize UBE3C on the RP (A) Crosslink-based PPI network of proteasome-associated proteins filtered for AP-MS enrichment. (B) Protein sequence map of UBE3C crosslinked residues to proteasome subunits, with domains and catalytic C1051 indicated. (C) Immunoblots showing displacement of endogenous UBE3C from RPN1-purified proteasomes after overexpression of UBE3C^N-term^-GFP. PSMB5 and β-actin are controls. (D) Immunoblot for UBE3C of samples from a pull-down experiment in which glutathione sepharose resin bound to GST-RPN10^UIM1–2^, GST-RPN10^UIM1^, or GST (control) was incubated with HCT116 cell lysates and immunoprobed with anti-UBE3C antibodies (left). Ponceau S staining of the polyvinylidene fluoride (PVDF) membrane served as a loading control for bound proteins (right). (E) Immunoblots from a pull-down experiment from RPN1-tagged or RPN10^VWA^ HCT116 cells using biotinylated MC1 against RPN1. As a control, the biotinylated peptide was omitted. (F) Alignment strategy placing the AlphaFold3 UBE3C-RP subcomplex model onto the substrate-engaged 26S proteasome structure PDB: 6MSE. The CP β-ring is not shown. (G) UBE3C and the local proteasome environment. The model positions UBE3C near RPN2, RPN3, RPN10, and RPN11, with key PhIX-MS residue pairs highlighted. (H) Proposed model for coordinated UBE3C and RPN11 activities in which UBE3C ubiquitinates proteasome-bound substrates near RPN11 to support processive substrate translocation. The model structure is generated from that in (G) and follows the same coloring scheme. See also Figures S3 and S4.

Despite the large number of intra-proteasome crosslinks detected, only a limited number of crosslinks were between canonical proteasome subunits and transient interactors (Figure S3F). Correspondingly, the PSMB4 dataset identified only seven RP binders (Figure S3G). RP assembly chaperones, including PSMD5, PSMD10, PSMD9, and PAAF1, dissociate from the RP prior to or upon 26S proteasome formation and are therefore not expected to be represented in CP-tagged pull-downs. Consistently, RP subunits account for only ~25% of total CP LFQ intensity in PSMB4 pull-downs (Figure S2G), confirming that this dataset is dominated by CP-proximal interactions. Structural flexibility and the mild crosslinking conditions used may additionally limit the detection of any RP-associated proteins that transiently contact the assembled CP. The 276 PPIs in the combined RPN1 and RPN11 datasets have an estimated false discovery rate (FDR) of 7.7% based on our target-decoy approach (Data S3). These false PPI matches are unlikely to be between intrinsic proteasome subunits, and we therefore assumed them to concentrate among the potential proteasome interactors. To remove likely false matches, we filtered out crosslinking interactions involving proteins that were enriched in the AP-MS samples (Figures 1C, 1D, and 1F). This filtering step reduced the number of proteins identified with direct crosslinks to intrinsic proteasome subunits from 30 (Figure S3F) to 16 (Figure 2A). Of these, experimental structures are available for proteasome complexes for PSME1/2^45^ (Figure S4A, PDB: 7DR6), ubiquitin chains (Figure S4B, PDB: 8JTI), and the deubiquitinase USP14^46,47^ (Figure S4C, PDB: 7W37), all of which are consistent with the measured crosslinks. Similarly, the structure of UCHL5^48,49^ with the RPN13 DEUBAD (deubiquitinase adaptor) domain and ubiquitin is consistent with our crosslinking data (Figure S4D, PDB: 4UEL). These findings provide confidence that the determined structures are adopted in the cellular context and that our crosslinking data can be applied to provide high-fidelity structural information.

We used AlphaFold2-Multimer^50^ to predict the binding sites of the proteins directly crosslinked to the RP. As proteins may have additional interfaces with subunits of the proteasome other than those found crosslinked, we predicted pairwise structural models for the 19 established RP subunits for a total of 270 potential binary pairs (Data S5). Five models were generated per pair, and interface confidence was evaluated using the average model-confidence scores.^51^ Applying a stringent cutoff of 0.65 for average model-confidence yielded six novel interfaces: UBLCP1:RPN1, PSMD5:RPT1, UBE3C:RPN2, RAD23A:RPN1, RAD23B:RPN1, and PITHD1:RPN10 (Data S5; Figure S4E). UBLCP1 was predicted to bind the RPN1 T2 site through its UBL domain, where USP14 UBL binds,^42^ and this interaction is supported by eight unique crosslinks (Figure S4F). RAD23A (Figure S4G) and RAD23B (Figure S4H) were predicted by AlphaFold to bind to the RPN1 T1 site, as previously shown in *S. cerevisiae*,^42,52,53^ and a crosslink was identified between the RPN1 T1 and T2 sites that matches but cannot distinguish between RAD23A or RAD23B UBL (Figure S4E). PITHD1 is predicted to bind to the RPN10 VWA (von Willebrand factor type A) domain (Figure S4I) and, together with a crosslink to nearby RPN2, this binding location agrees with a recent preprint that reports the structure of PITHD1 at RPN10 and RPN2 on the proteasome.^54^

### PhIX-MS positions UBE3C along the upper rim of the RP

PhIX-MS produces experimental restraints that can rescue lower-confidence AlphaFold predictions. The structural positioning of UBE3C on the RP has remained incompletely defined.^31–33^ We identified crosslinks linking UBE3C to three RP subunits, RPN2, RPN3, and RPN10 (Figure 2B). AlphaFold2-Multimer predicted a high-confidence interface with RPN2, whereas candidate interfaces with RPN3 and RPN10 had lower confidence (Figures S4K–S4M). These lower-confidence interfaces were supported, however, by PhIX-MS, with two crosslinks to RPN3 and one to the RPN10 UIM (ubiquitin-interacting motif) region that was detected in two independent pull-down datasets (RPN11 and PSMB4).

The predicted interaction between RPN3 and UBE3C is consistent with previous evidence that the UBE3C disordered N-terminal region contributes to its proteasome association.^31^ To test this requirement directly, we expressed an N-terminal fragment of UBE3C fused to green fluorescent protein (GFP) (UBE3C^N-term^-GFP) in RPN1-tagged cells. UBE3C^N-term^-GFP reduced endogenous UBE3C at RPN1-purified proteasomes, supporting a role for the UBE3C N terminus in proteasome binding (Figure 2C).

One of the five UBE3C:RPN10-predicted structures suggested that UBE3C interacts with the RPN10 UIM1 region (Figure S4M). This interaction is supported by a crosslink between UBE3C K442 and RPN10 E226 that was detected in both RPN11 and PSMB4 pull-downs (Figures 2B and S3G; Data S6). To test further whether the RPN10 UIM region is involved in UBE3C binding, we expressed and purified glutathione S-transferase (GST)-RPN10^UIM1–2^ (203–310), which contains both UIMs, and GST-RPN10^UIM1^ (196–272), from *E. coli*. GST-RPN10^UIM1^ and GST-RPN10^UIM1–2^ each bound modestly to UBE3C in a pull-down assay with GST as a control (Figure 2D; Data S7), consistent with a direct but likely low-affinity interaction. To test whether the UBE3C interaction with the UIM region of RPN10 is functionally significant, we used a biotinylated macrocyclic peptide (macrocyclic peptide 1, MC1) against RPN1^55^ to pull down proteasomes from RPN1-tagged or RPN10^VWA^ HCT116 cells, the latter of which were gene-edited to truncate RPN10 at its VWA domain.^56^ Indeed, UBE3C was reduced at RPN10^VWA^ proteasomes (Figure 2E). Altogether, these data establish an interaction between UBE3C and the RPN10 UIM region that is consequential to UBE3C interaction with proteasomes.

To assess whether these interfaces are structurally compatible, we generated an AlphaFold3 model containing UBE3C together with RPN2, RPN3, RPN8, RPN9, RPN10, RPN11, and RPN12, which satisfied all three interaction sites (Figure S4N). After alignment to the substrate-engaged human 26S proteasome (PDB: 6MSE, Figure 2F), UBE3C could be positioned without major steric clashes (Figures 2G, S4O, and S4P). In this model, the UBE3C N terminus localizes near RPN3, while the central regions extend toward RPN2 and RPN10, placing UBE3C along the upper rim of the RP. Because no crosslinks were identified within the HECT domain, we do not assign a fixed position for the catalytic region and instead interpret it as conformationally dynamic at a location that is ideal for interaction with the RPN11-engaged substrate (Figure 2H).

### Proteasome-binding mechanism of TXNL1 by cryo-EM

**Figure 3.**
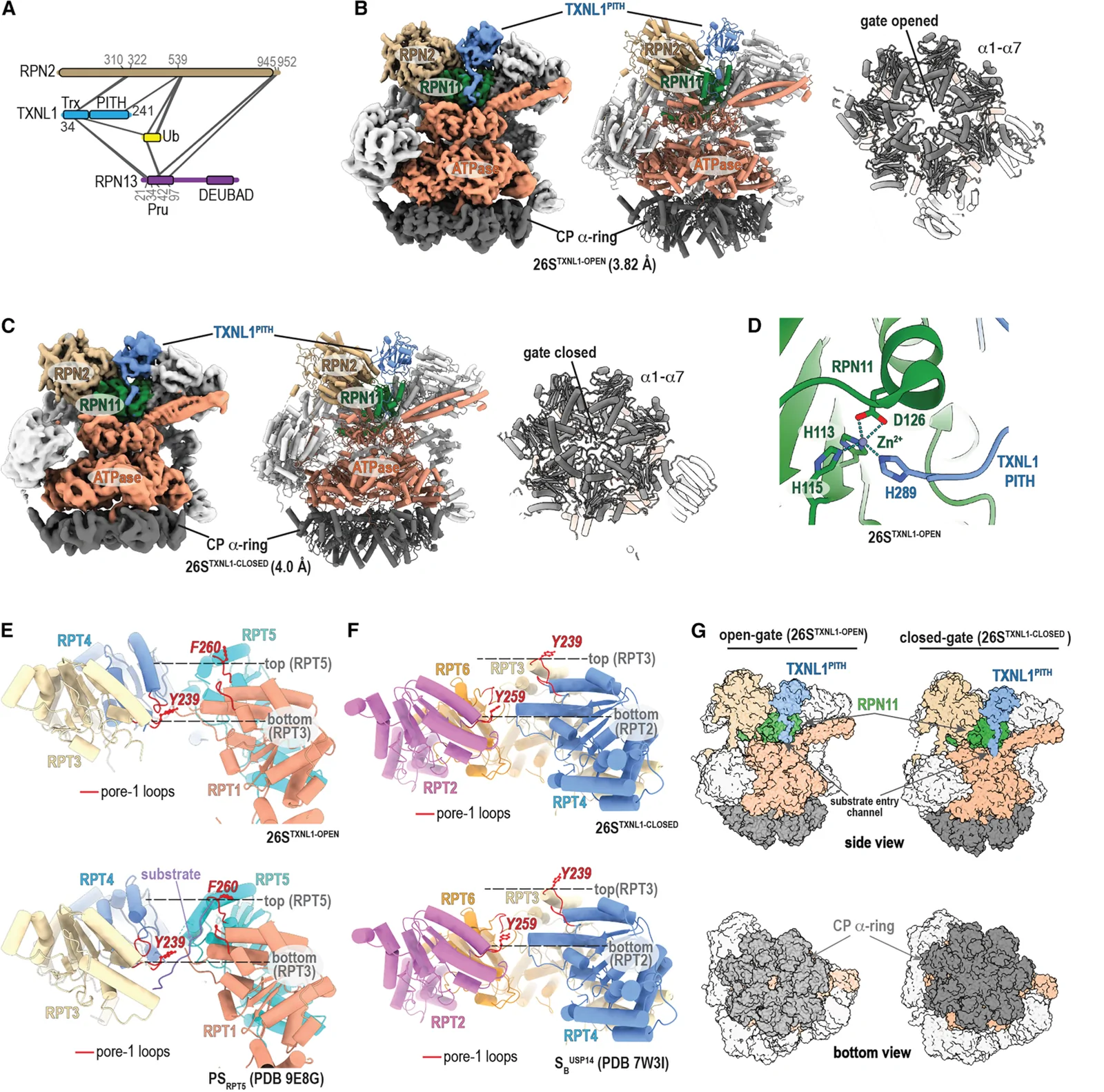
Structures of 26S proteasomes with TXNL1 determined by cryo-EM (A) Protein sequence map of crosslinked residue pairs between TXNL1 and proteasome subunits or ubiquitin. (B) Cryo-EM density map (3.82 Å, left) and corresponding ribbon diagram (middle) of 26S^TXNL1-OPEN^. The right panel shows a bottom view of the CP α-ring and the opened gate. (C) Cryo-EM density map (4.0 Å, left) and corresponding ribbon diagram (middle) of 26S^TXNL1-CLOSED^, colored as in (B). The right panel shows the closed gate CP conformation. (D) Expanded view of 26S^TXNL1-OPEN^ to display TXNL1 PITH domain H289 coordination with the RPN11 Zn^2+^ and RPN11 H113, H115, and D126. (E and F) Expanded views of the ATPase large AAA+ subdomains in aligned 26S^TXNL1-OPEN^ (E, top) and PS_Rpt5_ (PDB: 9E8G, E, bottom), or 26S^TXNL1-CLOSED^ (F, top) and S_B_^USP14^ (PDB: 7W3I, F, bottom). For clarity, RPT2 and RPT6 in (E), as well as RPT1 and RPT5 in (F), were omitted to improve visualization of the remaining structural features. (G) Cartoon depicting the TXNL1 PITH domain (blue) at the proteasome RP with an open (left) or closed (right) CP α-ring (gray). (B)–(G) were prepared using UCSF (University of California, San Francisco) ChimeraX. See also Table 1 and Figures S5 and S6.

To complement our MS data with high-resolution structural information, we collected cryo-EM data on RPN1-purified proteasomes (Figures S5A–S5AC). UBE3C was not observed, but TXNL1 was identified by selecting particles with additional density near RPN2 where the crosslinking data indicated an interaction (Figures 3A and S5A; Data S6). This effort resolved two distinct structural states, with the TXNL1 PITH domain observable at 26S proteasomes, one at 3.82 Å resolution with an opened CP gate (26S^TXNL1-OPEN^, Figure 3B; Video S1) and the other at 4.0 Å resolution with a closed CP gate (26S^TXNL1-CLOSED^, Figure 3C). In both, TXNL1 sits between RPN2 and the RPN10 VWA domain, with its C terminus crossing RPN11 and ending at the oligonucleotide/oligosaccharide-binding (OB) fold ring of the ATPase (Figures 3B, 3C, and S6A), consistent with our TXNL1 V241 crosslink to RPN2 T539 (Figures 3A and S6B). As in recently reported structures of the TXNL1 PITH (proteasome-interacting thioredoxin) domain at the 26S proteasome,^57,58^ the TXNL1 C-terminal H289 coordinates to the RPN11 Zn^2+^, along with RPN11 H113, H115, and D126 (Figures 3D, S6A, and S6C; Video S1).

The ATPase large AAA+ subdomains in 26S^TXNL1-OPEN^ adopt a spiral-staircase arrangement similar to the substrate-processing state PS_RPT5_ (PDB: 9E8G). In both cases, RPT5 is at the top of the staircase and RPT3 at the bottom (Figure 3E), with less interleaving of RPT4 with its neighboring subunits (Figure S6D). In contrast to PS_RPT5_, however, 26S^TXNL1-OPEN^ lacks substrate, and, in turn, the TXNL1 PITH and RPN10 VWA domains are laterally shifted toward the translocation channel, causing the RPT4:RPT5 coiled coil to be rotated outward (Figure S6E). By contrast, 26S^TXNL1-CLOSED^ adopts a typical resting-state conformation, with RPT3 at the top, RPT2 at the bottom (Figure 3F), and RPT6 mimicking a connecting seam (Figure S6F). 26S^TXNL1-CLOSED^ aligns more closely with USP14-bound 26S proteasomes (Figure 3F), although TXNL1 is not present in this complex^46^ (Figure S6G) and USP14 is not observed in 26S^TXNL1-CLOSED^. In summary, we find TXNL1 to bind to 26S proteasomes with both an opened and a closed CP α-ring, indicating that it can be present at both resting and substrate-processing proteasomes (Figure 3G).

### PhIX-MS helps position the TXNL1 Trx domain at RPN2

**Figure 4.**
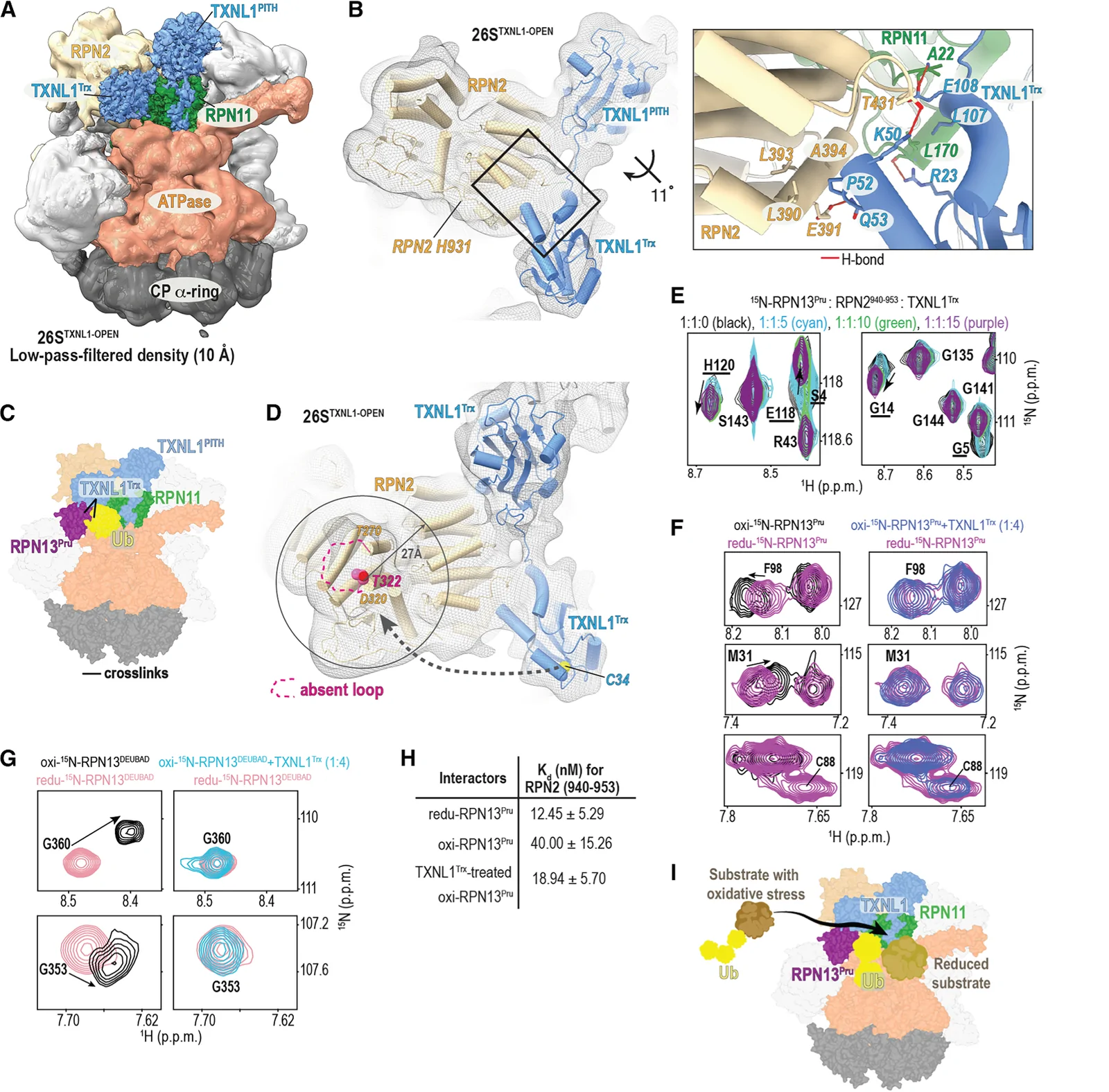
PhIX-MS guides placement of the TXNL1 Trx domain (A) TXNL1 Trx domain fitted into the extra density adjacent to RPN2 and RPN11 of a low-pass-filtered (10 Å) 26S^TXNL1-OPEN^ map. (B) Left: enlarged region of 26S^TXNL1-OPEN^ centered on RPN2 and TXNL1, with the density map displayed in gray. The extreme C terminus of RPN2 (G932-D953) is not visible. Right: enlarged view of the RPN2:TXNL1 interface, with contact residues indicated. (C) Cartoon based on (A) illustrating the expected location of RPN13 Pru (purple) with ubiquitin (yellow) bound. Two crosslinks are shown from TXNL1 C34 to RPN13 K34 and ubiquitin K48. (D) Structural view showing that crosslinks from TXNL1 C34 to RPN2 T322 are compatible with mobility of the partially unresolved RPN2 linker. A gray circle with a radius of 27 Å is displayed centered at Cα of RPN2 T322. (E) Expanded view of overlaid ^1^H, ^15^N HSQC spectra of 20 μM ^15^N-labeled RPN13^Pru^ preincubated with equimolar unlabeled RPN2^940–953^ (black), and with a 5-fold (cyan), 10-fold (green), or 15-fold (purple) molar excess of unlabeled TXNL1 Trx. RPN13^Pru^ signals that shift or attenuate following addition of unlabeled TXNL1 Trx are labeled and underlined. (F and G) Expanded view of overlaid ^1^H, ^15^N HSQC spectra of 22 μM ^15^N-labeled RPN13^Pru^ or 25 μM ^15^N-labeled RPN13^DEUBAD^ in reduced (redu, shades of pink) or oxidized (oxi) states without (black) or with a 4-fold molar excess of the TXNL1 Trx domain (shades of blue). The enlarged regions focus on (F) F98 (top), M31 (middle), and C88 (bottom) or (G) G360 (top) and G353 (bottom). Spectra were collected at 600 MHz, 10°C, and in NMR buffer containing 20 mM sodium phosphate and 50 mM NaCl (pH 6.0) with 1 mM DTT in the reduced samples. (H) Binding affinity, K_d_ (nM), determined by ITC for RPN2 (940–953) and redu or oxi RPN13^Pru^, and oxi-RPN13^Pru^ following incubation with a 3-fold molar excess of TXNL1^Trx^. TXNL1^Trx^ was removed prior to measurements by size-exclusion chromatography. (I) Model in which TXNL1 reduces oxidized proteasome substrates or proteasome-associated proteins before substrate translocation. See also Table 1 and Figure S6.

Dropping the map threshold from 0.04 to 0.012 revealed additional density in 26S^TXNL1-OPEN^ extending from TXNL1 PITH, into which the TXNL1 thioredoxin (Trx) domain fit well (Figures 4A and 4B; Video S1). This placement locates Trx proximal to the RPN2 C terminus where the RPN13 N-terminal Pru (pleckstrin-like receptor for ubiquitin) domain binds,^59–61^ consistent with a crosslink between RPN13 K34 and TXNL1 C34 (Figures 3A and 4C). However, this positioning cannot account for two identified crosslinks from TXNL1 C34 to RPN2 K310 and T322—residues located within a partially unresolved linker region (Figures 3A and 4D; Data S6). This inconsistency may arise from the flexible connection between the Trx and PITH domains, which could facilitate Trx domain movement relative to the PITH domain (Figure 4D). Neither RPN13 nor the RPN2 C terminus was resolved in the density map, most likely due to the dynamics of this region.^61^ We therefore used NMR to test whether RPN13 Pru binds to Trx. Unlabeled TXNL1 Trx domain (TXNL1^Trx^) was added at a 5-, 10-, or 15-fold molar excess to 20 μM ^15^N-labeled RPN13 (1–150), preincubated with equimolar quantities of its proteasome-binding site (RPN2 [940–953]), and the effects were monitored by 2D ^1^H, ^15^N HSQC (heteronuclear single quantum coherence) experiments. Only minor signal shifting was observed, which occurred for amino acids in the helix, such as E118 and H120, and at the N terminus, including G5 and G14 (Figure 4E). These effects likely reflect non-specific binding, as the Pru helix and N terminus interact broadly with peptide sequences.^62^ Therefore, we conclude that TXNL1^Trx^ does not form a stable interaction with RPN13 Pru but interacts transiently.

Because TXNL1 alleviates oxidative stress by reducing disulfide bonds,^63^ we tested whether it is active against oxidized RPN13, which has five cysteines, including C88, positioned peripherally to the proteasome-binding site^59,60^ and susceptible to Michael addition.^64–67^ The ^15^N-labeled RPN13 Pru or DEUBAD domains were separately oxidized by incubating them on ice for 10 min with 0.05% H_2_O_2_ and, following removal of H_2_O_2_, evaluated by MS and 2D NMR experiments to confirm their oxidation. Addition of TXNL1^Trx^ restored the oxidized RPN13 Pru and DEUBAD signals to their reduced position in 2D ^1^H, ^15^N HSQC experiments (Figures 4F, 4G, S6H, and S6I). We next tested the impact of oxidation and TXNL1^Trx^ restoration on RPN13 Pru affinity for RPN2 (940–953) using isothermal titration calorimetry (ITC). RPN13 Pru was measured to bind RPN2 with an affinity of 12.45 ± 5.29 nM, consistent with previous measurements.^60^ Following oxidation, this affinity remained high but was reduced to 40.00 ± 15.26 nM. We next measured the binding affinity after RPN13 Pru was restored by TXNL1^Trx^, which was removed prior to ITC, to find a value of 18.94 ± 5.70 nM (Figures 4H and S6J). These results indicate a small reduction in the affinity of oxidized RPN13 Pru for RPN2 and that TXNL1^Trx^ can rescue RPN13 from oxidative stress to restore RPN2 affinity. Future studies are needed to determine whether RPN13, particularly C88 near the RPN2-binding site, is oxidized at proteasomes and whether TXNL1 restores it in cells.

A general increase in oxidized proteins is not observed following TXNL1 loss, with or without oxidative stress,^57^ suggesting that TXNL1 may act on specific substrates or proteasome-associated proteins under defined stress conditions. Altogether, we find the TXNL1 PITH domain caps RPN11, while its Trx domain extends toward RPN2 near the RPN13-binding site, positioning TXNL1 to reduce oxidized substrates prior to their passage into the substrate entry channel (Figure 4I).

### PSMD5 binds the ATPase ring to regulate RP maturation

**Figure 5.**
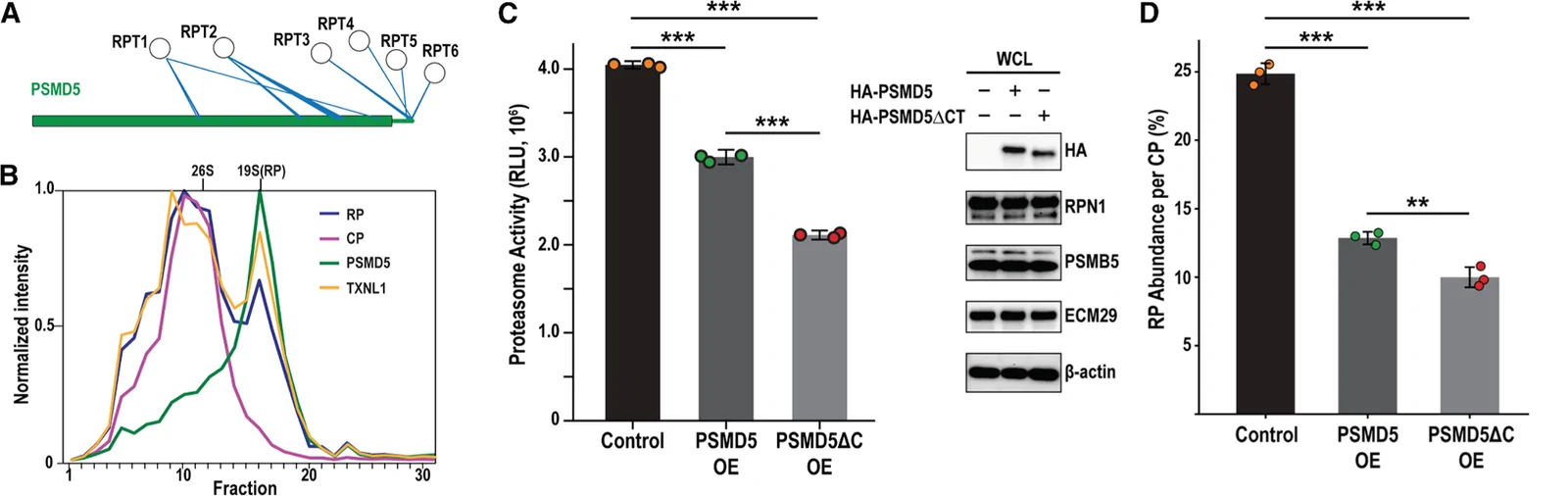
PSMD5 binds to the intact RP (A) PSMD5 sequence map showing crosslinks to all six RP ATPase subunits, including crosslinks within its disordered C-terminal tail. (B) Co-fractionation MS data from a crosslinked RPN1 pull-down (size-exclusion chromatography) showing that PSMD5 elutes with the free RP. TXNL1 elutes with both the 26S proteasome and the free RP. (C) Chymotrypsin-like activity assays showing reduced proteasome activity after HA-PSMD5 or HA-PSMD5ΔC overexpression in RPN1-tagged cells. Bars represent the mean ± SD of three independent biological replicates. *p* values: *p < 0.05, **p < 0.01, ***p < 0.001, ns for *p* ≥ 0.05). Immunoblots confirm expression of HA-PSMD5 or HA-PSMD5ΔC, with RPN1, PSMB5, ECM29, and β-actin as a control. (D) Percentage abundance of RP subunits without (control) or following overexpression of HA-PSMD5 or HA-PSMD5ΔC in PSMB4 pull-down experiments. The data were normalized to the mean RP subunit MaxLFQ intensity within each replicate. Replicates and *p* values are as in (C). See also Figures S7 and S8.

PSMD5 was strongly enriched in both crosslinked RPN1 and RPN11 pull-downs, with LFQ intensities comparable to those of canonical RP subunits, whereas it was not significantly enriched in PSMB4 pull-downs (Figures 1C, 1D, 1F, and S2E–S2J). PSMD5 has been implicated in RP assembly^36^ and inhibition of 26S proteasome formation.^37,38,68^ We identified 31 combined crosslinks between PSMD5 and the ATPase ring in proteasomes purified by RPN1 and RPN11 (Figure 5A; Data S6), together with a high-confidence AlphaFold model of PSMD5 bound to RPT1 (Figure S4J), which is consistent with the determined crystal structure for the yeast ortholog.^69,70^ To define the stage at which PSMD5 associates with the proteasome, we performed co-fractionation MS on crosslinked RPN1 pull-downs. PSMD5 co-eluted with intact free RP at abundances comparable to those of RP subunits, suggesting that the majority of mature free RP particles are PSMD5-bound before CP engagement (Figure 5B).

Because crosslinks were observed between PSMD5 and all six ATPase subunits (Figure 5A; Data S6), we used AlphaFold3 to predict PSMD5 bound to the complete ATPase hexamer. A high-confidence model satisfied 30 of 31 crosslinks (Figures S7A and S7B). The ordered core of PSMD5 engaged RPT1 and RPT2 in an orientation that would be incompatible with 26S assembly. The disordered C-terminal tail of PSMD5 was projected into the central pore of the ATPase ring, a feature supported by a cluster of crosslinks (Figure S7C). Consistent with this model, PSMB4 pull-downs did not enrich PSMD5 (Figures S2H–S2J), supporting the idea that PSMD5 associates with free RP but is excluded from mature RP-CP holoenzymes.

To test whether elevated PSMD5 levels alter holoenzyme assembly, we overexpressed hemagglutinin (HA)-PSMD5 in HCT116 cells. HA-PSMD5 significantly inhibited 26S proteasome activity (Figure 5C; Data S8), consistent with its role as a negative regulator of 26S proteasome assembly.^38^ To determine whether this inhibition reflects altered 26S/30S proteasome abundance, we performed PSMB4 and RPN1 pull-downs following overexpression of PSMD5 or PSMD5ΔC. In PSMB4 pull-downs, both PSMD5 and PSMD5ΔC reduced CP association with the RP (Figure 5D), in agreement with the activity measurements. Likewise, RPN1 pull-downs showed reduced CP association with the RP, whereas the molar ratio of RP subunits to PSMD5 approached 1:1, consistent with the co-fractionation data (Figure S8A). RP subunit distribution was unchanged, arguing against a major defect in RP abundance or stability (Figure S8B). Notably, PSMD5ΔC inhibited 26S proteasome formation to a greater extent than full-length PSMD5 (Figure 5D), indicating that the C-terminal tail either reduces PSMD5 affinity for the RP or promotes RP engagement with the CP.

Together, these data support a model in which PSMD5 associates with the free RP, engages the ATPase ring, and stabilizes a state that is incompatible with RP-CP coupling. These findings extend PSMD5 function beyond being a passive assembly chaperone and position it as a gatekeeper of RP-CP coupling.

### Structure of the PSMD5-RP complex by PhIX-MS and cryo-EM

**Figure 6.**
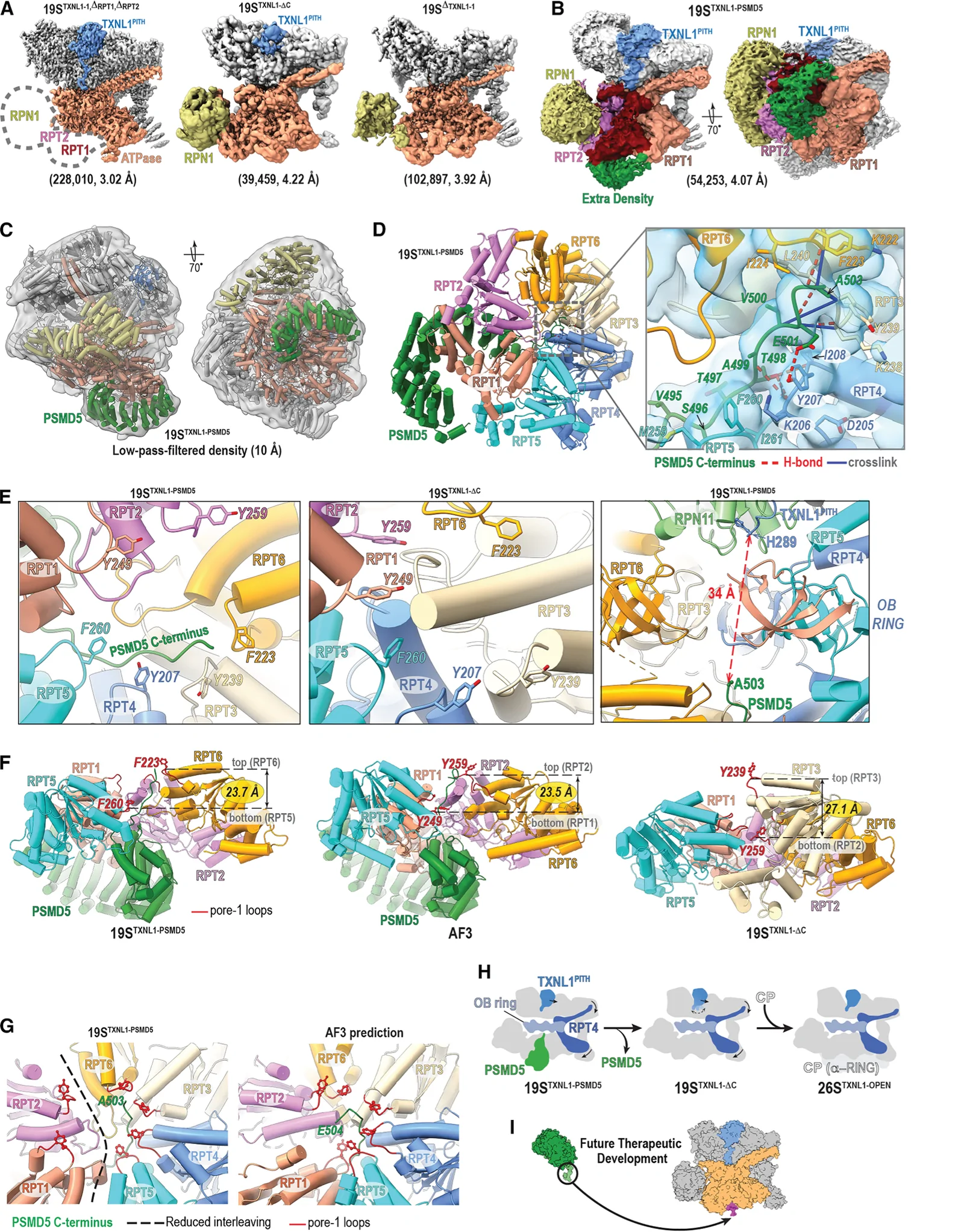
Structures of the 19S RP with TXNL1 and PSMD5 determined by cryo-EM (A) Cryo-EM density map of 19S^TXNL1–1, ΔRPT1, ΔRPT2^ (3.02 Å, left), 19S^TXNL1-ΔC^ (4.22 Å, middle), and 19S^ΔTXNL1–1^ (3.92 Å, right). (B) Cryo-EM density map of 19S^TXNL1-PSMD5^ (4.07 Å), with extra density adjacent to RPT1-RPT2 corresponding to PSMD5. (C) PSMD5 fitted into the extra density adjacent to RPT1 and RPT2 of a low-pass-filtered (10 Å) 19S^TXNL1-PSMD5^ map. (D) Left: ribbon diagram showing the ATPase central channel with the PSMD5 C terminus inserted. Right: expanded view of the structural region with the corresponding density map, displaying the contact surface between the PSMD5 C-terminal residues and RPT subunits. (E) Comparison of the 19S^TXNL1-PSMD5^ ATPase pore (left) with 19S^TXNL1-ΔC^ (middle), and a view showing the distance between the PSMD5 and TXNL1 C termini (right). (F) Expanded view of the ATPase large AAA+ subdomain in aligned 19S^TXNL1-PSMD5^ (left), the AlphaFold3-predicted model of PSMD5 bound to the ATPase ring (middle), and 19S^TXNL1-ΔC^ (right). The Cα positions of the top and bottom Tyr/Phe residues in pore-1 loops are highlighted and labeled by black dashed lines. Cα-Cα distances between the top and bottom Tyr/Phe residues in pore-1 loops are displayed. For clarity, RPT3 and RPT4 were omitted from the left and middle panels, respectively, with RPT4 also omitted from the right panel. PSMD5 is included in dark green in the left and middle panels. (G) Top views of the ATPase large AAA+ subdomains in 19S^TXNL1-PSMD5^ (left) and the aligned AlphaFold3-predicted model of PSMD5 bound to the ATPase ring (right). The color scheme is as in (F). The reduced interleaving between RPT1/RPT2 and their neighboring RPT subunits in 19S^TXNL1-PSMD5^ (left) is highlighted by a black dashed line. (H) Cartoon showing that PSMD5 engagement at the CP-facing side of the RP is allosterically coupled across the OB ring to distal RP regions. Arrows indicate the direction of motion in the RP following PSMD5 (green) release and CP capping. (I) Model for using the PSMD5 C-terminal ATPase-binding sequence as a starting point for inhibitors that block 26S/30S assembly. See also Table 2 and Figure S9.

In our cryo-EM dataset, we observed free RP particles (with no CP present) and selected these for further refinement (Figures S5A and S5B). Three distinct states were observed: two with TXNL1 PITH present; one with poor resolution for RPN1, RPT1, and RPT2 (19S^TXNL1–1,**Δ**RPT1, **Δ**RPT2^, Figure 6A, left), another in which the TXNL1 C-terminal tail is not visible, designated 19S^TXNL1-ΔC^ (Figures 6A, middle and S9A); and a third without TXNL1 present (19S^**Δ**TXNL1–1^, Figure 6A, right). We applied 3D classification and 3D variability analyses followed by local refinement masking on 19S^TXNL1–1,**Δ**RPT1,**Δ**RPT2^, focused on the RPT1-RPT2-RPT5-RPT6 AAA+ domain and RPN1 (Figures S5B and S9B). This data processing yielded two distinct states with RPN1 and the AAA+ domain of RPT1 and RPT2 present, one consistent with PSMD5 (19S^TXNL1-PSMD5^, Figure 6B; Video S2) and another with PSMD10 (19S^TXNL1-PSMD10^, Figure S9C). We fit PSMD10 into 19S^TXNL1-PSMD10^ (Figure S9C) using a previously resolved structure of PSMD10-bound RP from human embryonic kidney (HEK) 293 cells.^71^ This structure lacked TXNL1.

PSMD5 was apparent in 19S^TXNL1-PSMD5^ after applying a 10-Å low-pass filter (Figure 6C). Its C-terminal tail side-chain atoms spanning S496-A503 were unambiguously fitted into the density of a map sharpened by DeepEMhancer,^72^ while its terminal residue E504 was not observed (Figure 6D, right; Video S2). All crosslinks were satisfied except for those between RPT1 A289 and PSMD5 K461 (Figures 6D and S9D; Data S6). PSMD5 was positioned against RPT1 and RPT2, with its C terminus inserted into the ATPase channel where it forms extensive interactions with RPT3, RPT4, RPT5, and RPT6 (Figures 6D and 6E). These interactions induce massive rearrangements in the ATPase ring (Figure 6E, left, compared with Figure 6E, middle), causing the distance between the uppermost and lowermost pore loops to flatten. Specifically, the Cα-Cα distance between the pore-1 loop of the top-most and bottom-most central Phe/Tyr is 23.7 Å for 19S^TXNL1-PSMD5^ (Figure 6F, left) compared with >27 Å in the substrate-processing and resting 26S proteasomes (Figure S9E). 19S^TXNL1-PSMD5^ also has a unique ATPase configuration, with RPT6 at the top and RPT5 at the bottom (Figure 6F, left), as previously reported structures pair top-most RPT6 with bottom-most RPT2 (PDB: 9E8K, Figure S9E, top) and bottom-most RPT5 with top-most RPT2 (PDB: 9E8O, Figure S9E, middle).

The 19S^TXNL1-PSMD5^ ATPase OB ring separates PSMD5 from TXNL1, enforcing a 34-Å distance between the C-terminal TXNL1 H289 and the PSMD5 penultimate A503 (Figure 6E, right). Overlaying the 19S^TXNL1-PSMD5^ OB ring with that of the TXNL1-bound 26S proteasome (both the open-and closed-gated structures) or 19S^TXNL1-**Δ**C^ reveals a rotation in the AAA+ ATPase ring in response to PSMD5 binding that corresponds to structural rearrangements at the other side of the OB ring, including shifting of the coiled coils (Figure S9F; Video S3), TXNL1 PITH, and RPN11 (Figure S9G; Video S3). Thus, PSMD5 binding at the CP side of the RP is allosterically coupled across the OB ring.

When aligning 19S^TXNL1-PSMD5^ with the ATPase-PSMD5 AlphaFold3 model (gray), RPT1 (red), RPT2 (magenta), RPT5 (cyan), and RPT6 (orange) show poor agreement (Figure S9H). AlphaFold3 predicted RPT2 to be at the top and RPT1 at the bottom of the ATPase ring, with the PSMD5 C terminus interacting with the pore-1 loops of all six ATPase subunits rather than only those of RPT3-RPT6 (Figure 6G). Nonetheless, AlphaFold predicted the flatter pore-1 loop distance, measuring 23.5 Å (Figure 6F, middle). Reduced inter-subunit interleaving was observed in 19S^TXNL1-PSMD5^ between RPT1/RPT2 and their neighboring subunits (Figure 6G, left), akin to that induced by PSMD10 for RPT3/RPT6 and their neighboring subunits^71^ (Figure S9I). No such reduction in inter-subunit interleaving was predicted by AlphaFold3 (Figure 6G, right).

Extra density was also observed at 19S^**Δ**TXNL1–2^, where PSMD5 binds (Figure S9J, right, with the 15-Å low-pass-filtered density on the bottom), which was identified by 3D variability analyses and masked local refinement of 35,258 particles from 19S^ΔTXNL1–1^ (Figure S5B). This density, however, was too small to fit. Additional density that could correspond to either PSMD5 or PSMD10 was not observed for 19S^TXNL1-ΔC^ (Figure 6A), which included only 39,459 particles (Figure S5B). 19S^TXNL1-ΔC^ also lacks the flattened pore-1 loop configuration (Figure 6F, right). Its ATPase ring mimics the 26S proteasome resting state, with RPT3 positioned at the top, RPT2 at the bottom, and RPT6 at a seam position bound to ADP (Figures 6F, right and S9K), analogous to a previously observed resting TXNL1-bound 26S proteasome state.^58^

Altogether, we find that PSMD5 sterically occludes the CP as its C-terminal tail inserts into the RP ATPase ring, weakening RPT1-RPT2 interleaving with their neighboring subunits and flattening the pore-1 loop configuration. Moreover, our structural comparisons indicate that these changes are allosterically sensed across the OB ring (Figure 6H).

## DISCUSSION

In this study, we combined PhIX-MS, cryo-EM, and computational modeling to map dynamic proteasome interactions in cells. By applying *in situ* photo-crosslinking before proteasome purification, we preserved labile interactions that were difficult to resolve by cryo-EM alone and obtained residue-level restraints for structural interpretation. Several known regulators, including UBE3C, were significantly enriched only in crosslinked samples.

Combining *in situ*-derived crosslinks with AlphaFold predictions allowed us to generate an experimentally constrained model for the UBE3C interaction with the proteasome. In this model, the UBE3C N terminus contacts RPN3, while its central regions span RPN2 and the RPN10 UIM region, placing the dynamic HECT domain near the RPN11 active site. A recent preprint describing the reconstituted cryo-EM structure of UBE3C bound to the proteasome^73^ independently corroborates the binding interfaces identified here, with the exception of the RPN10 UIM region that PhIX-MS was able to capture. The RPN10 UIM region is highly dynamic,^74^ which would make its observation by cryo-EM challenging. The positioning of UBE3C provides a rationale for how it may ubiquitinate stalled or slowly degraded substrates to prevent their premature release, as RPN11 acts to remove ubiquitin chains at the substrate-proximal end during translocation into the ATPase pore (Figure 2H).

The UBE3C paralog UBE3A binds proteasomes at the extreme C terminus of RPN10^12^; thus, UBE3A and UBE3C occupy distinct proteasome sites and likely have non-equivalent functions. Loss-of-function UBE3A mutations drive Angelman syndrome,^75–77^ whereas its elevated gene dosage correlates with autism spectrum disorders.^78^ Like UBE3A, biallelic loss-of-function mutations in UBE3C associate with Angelman-like symptoms.^79^ Thus, these two ligases are unable to compensate for each other despite their dual presence at proteasomes. In addition, a UBE3C intergenic fusion that loses its HECT domain is linked to distal hereditary motor neuropathy.^80^ In addition to these neurological disorders, UBE3C is also a therapeutic target for melanoma,^81^ glioma,^82^ renal cell carcinoma,^83^ breast cancer,^84^ and cystic fibrosis.^85^

We resolved TXNL1 and PSMD5 at proteasomes by cryo-EM, but their intrinsic dynamics posed challenges. Whereas TXNL1 PITH was readily observed, its Trx domain yielded weaker density, and the *in situ* crosslinks provided critical independent support for its placement (Figures 3A and S6B). TXNL1 positioning near RPN11 places it at a key point in the substrate-processing pathway, where it could reduce oxidized substrates or proteasome-associated proteins before translocation into the CP. PhIX-MS revealed that PSMD5 interacts with all six RPT subunits, and these crosslinking data were critical to resolving the structure of PSMD5-bound RP. With its C terminus inserted into the central pore of the ATPase hexamer, PSMD5 weakens RPT interleaving to generate a unique, flatter pore-1 loop structure. Co-fractionation MS suggests that PSMD5 is likely bound to most free RP particles in HCT116 cells, and our structure suggests that PSMD5 interactions with the RPT subunits stabilize the ATPase ring until full assembly of the RP. Interestingly, PSMD5ΔC inhibited 26S proteasome assembly to a greater extent than full-length PSMD5, suggesting that the pore-inserted tail does not simply block assembly but may also prime PSMD5 for release or CP engagement.

Structural comparison of 19S^TXNL1-PSMD5^ with 26S^TXNL1-OPEN^, 26S^TXNL1-CLOSED^, and 19S^TXNL1-ΔC^ indicated that interactions between the PSMD5 C terminus and the nucleotide-binding domain of the ATPase ring induce allosteric changes in the coiled-coil region, TXNL1 PITH, and RPN11. Whether this allostery promotes PSMD5 release, CP capping, or ATPase-driven maturation remains to be tested. A role for PSMD5 in modulating 26S proteasome formation is consistent with its known downregulation in cancer,^68^ as these cells increase proteasome activity to adapt to increased proteotoxic stress. PSMD5 may be fine-tuned to levels that enable RP assembly with seamless release and generation of 26S/30S proteasomes. Inhibitors of the proteasome CP are the standard of care for hematological cancers,^2^ and the ATPase-binding C-terminal sequence provides a potential starting point for strategies that block proteasome assembly rather than CP catalytic activity (Figure 6I).

In conclusion, PhIX-MS provides a way to stabilize native proteasome assemblies in cells and obtain residue-level restraints for structural analyses. In this study, we revealed how UBE3C, TXNL1, and PSMD5 engage proteasome complexes to regulate their assembly and promote their core activities. Our integrative approach establishes a generalizable framework for mapping dynamic protein assemblies in cells and offers new insights into the cellular logic of proteasome regulation by PSMD5 and the activities of UBE3C and TXNL1.

### Limitations of the study

Photo-activated crosslinking is inefficient due to the high propensity of the activated diazirine to quench with water.^29^ As a result, crosslinked residue pairs were not detected for many crosslinked proteins that were enriched in the *in situ* crosslinked AP-MS experiments. Continued improvements in MS sensitivity should reduce this limitation. In addition, cells were exchanged into PBS during SDA incubation to limit crosslinker quenching, which may introduce cellular stress. All experiments were performed in HCT116 cells under standard culture conditions, and the proteasome interactome may differ across cell types,^15^ stress conditions,^18^ or developmental states. This study also did not establish direct in-cell evidence for RPN13 oxidation or TXNL1-dependent reduction at proteasomes. The weak interaction between RPN13 and TXNL1 suggests that these proteins do not form a stable off-proteasome complex, and future work will be needed to define the physiological substrates and stress conditions that require TXNL1 at proteasomes. Finally, the role of the PSMD5 C-terminal tail remains unresolved. Although this region inserts into the ATPase pore in the PSMD5-bound RP structure, deleting it caused stronger inhibition of 26S proteasome formation. This suggests that the tail is not simply a steric block to CP binding but may instead help prime the RP for PSMD5 release or CP engagement. Future studies are needed to pursue the functional roles of TXNL1 and PSMD5, potentially capturing the PSMD5-mediated handoff of the RP to the CP or the PSMD5ΔC-bound RP. Nonetheless, comparison of the PSMD5-bound state with 26S proteasomes indicates allosteric coupling across the ATPase OB ring, suggesting that PSMD5 release may be coupled to events occurring at the other side of the RP (Figure 6H).

## RESOURCE AVAILABILITY

### Lead contact

Further information and requests for resources and reagents should be directed to and will be fulfilled by the lead contact, Kylie J. Walters, kylie.walters@nih.gov.

### Materials availability

All unique/stable reagents generated in this study are available from the lead contact with a completed materials transfer agreement.

### Data and code availability

- The cryo-EM maps generated in this study have been deposited in the EMDB database with accession codes EMDB: EMD-71740 (26S^TXNL1-OPEN^), EMDB: EMD-71741 (26S^TXNL1-CLOSED^), EMDB: EMD-71813 (19S^TXNL1–1^, ^ΔRPT1^, ΔRPT2), EMDB: EMD-71810 (19S^TXNL1-PSMD5^), EMDB: EMD-76283 (19S^TXNL1–2^), EMDB: EMD-76415 (19S^TXNL1-PSMD10^), EMDB: EMD-71737 (19S^TXNL1-ΔC^), EMDB: EMD-71791 (19S^ΔTXNL1–1^), and EMDB: EMD-71795 (19S^ΔTXNL1–2^). The atomic models have been deposited in the PDB with accession codes PDB: 9PMO (26S^TXNL1-OPEN^), PDB: 9PMQ (26S^TXNL1-CLOSED^), PDB: 9PRT (19S^TXNL1–1,ΔRPT1,ΔRPT2^), PDB: 9PRO (19S^TXNL1-PSMD5^), PDB: 12BM (19S^TXNL1–2^), and PDB: 9PMJ (19S^TXNL1-ΔC^). Raw micrographs have been deposited in EMPIAR with accession number EMPIAR: EMPIAR-12891. All raw and processed pull-down-ID mass-spectrometry datasets used in this study are available in the PRIDE database under accession codes: pull-down ID-1 (non-crosslinked wild-type [WT] vs. RPN1 tag), PRIDE: PXD066281; pull-down ID-2 (crosslinked WT vs. RPN1 tag), PRIDE: PXD066280; pull-down ID-3 (non-crosslinked WT vs. RPN11 tag), PXD066279; pull-down ID-4 (crosslinked WT vs. RPN11 tag), PRIDE: PXD066278; pull-down ID-5 (non-crosslinked WT vs. PSMB4 tag), PRIDE: PXD066268; pull-down ID-6 (crosslinked WT vs. PSMB4 tag), PRIDE: PXD066284; and PSMD5 overexpression pull-down ID, PRIDE: PXD066535 and PXD076795. All raw files and processed crosslinking MS datasets are available in the PRIDE database under accession codes PRIDE: PXD066071, PXD066061, PXD066021 and PXD066073, and the co-fractionation MS dataset is available in the PRIDE database under accession code PRIDE: PXD066287. The AlphaFold3 model containing UBE3C, RPN2, RPN3, RPN8, RPN9, RPN10, RPN11, and RPN12 is available from ModelArchive under accession code ma-gxnsh. All deposited data are publicly available as of the date of publication. Original immunoblot images have been deposited in Mendeley at DOI: https://doi.org/10.17632/zcpm9k38yr.1 and are publicly available as of the date of publication.
- The custom Python scripts used for plotting and analyzing the proteasome activity assay and the quantitative comparison of PSMD5 overexpression are available at DOI: https://doi.org/10.5281/zenodo.20707940. The AlphaFold2 prediction analysis Python pipeline is available at DOI: https://doi.org/10.5281/zenodo.20708496. They are publicly available as of the date of publication.
- Any additional information required to reanalyze the data reported in this paper is available from the lead contact upon request.

## STAR★METHODS

### EXPERIMENTAL MODEL AND STUDY PARTICIPANT DETAILS

#### *Escherichia coli* strains

BL21 (DE3) and pLysS (DE3) cells were used in this study for the production of recombinant proteins. The cells were grown in either Luria-Bertani broth or M9 minimal media with ^15^N-ammonium chloride as the nitrogen source at 37°C in a shaker at 200 rpm until the optical density at 600 nm reached 0.5–0.6. Protein expression was induced by addition of 0.5 mM of isopropyl-1-thio-β-D-thiogalactopyranoside.

#### Mammalian cell culture

HCT116 or HEK293T cells were obtained from American Tissue Culture Collection (ATCC) and cultured at 37°C in a humidified environment of 5% CO_2_ in McCoy’s 5A modified media (Life Technologies Corp) or DMEM (Thermo Fisher Scientific) and supplemented with 10% fetal bovine serum (Thermo Fisher Scientific or Gemini Bio-Products). Modified HCT116 cells such as RPN1-tagged, RPN11-tagged and PSMB5-tagged cells were generated in this study by CRISPR/Cas9 editing as described in method details. The RPN10^VWA^ cell line was generated as previously described^56^ with guide RNAs designed near the point of truncation and at the final protein coding exon. Oligonucleotides corresponding to these guide RNAs were cloned into a modified pX458 vector (Addgene 48138) with eGFP replaced by mCerulean. pX458 was a gift from Feng Zhang.^89^ An HDR donor plasmid was constructed by genomic PCR and isothermal assembly, inserting a P2A-puromycin cassette immediately downstream of G194 and introducing silent mutations at the sgRNA target sites. Cas9-sgRNA plasmids and the HDR donor plasmid were co-transfected into HCT116 cells, followed by puromycin selection. Single-cell clones were isolated and genotyped to confirm correct knock-in of the desired modification.

### METHOD DETAILS

#### Design of CRISPR/Cas9 and donor plasmids

Guide RNAs (gRNAs) were designed in the region encompassing the final protein coding exon and 3′UTR using *sgRNA Scorer 2.0*^92^ (Table S1) and were tested for cutting activity in HEK293T cells. The chromosomal location and gRNA-binding regions within *PSMD14 and PSMB4* are listed in Table S2. Oligonucleotides corresponding to candidates IVT-2995, IVT-2997, JT-IVT-44 and JT-IVT-48, which were chosen for subsequent knock in experiments and named RPN11–01, RPN11–02, PSMB4–01 and PSMB4–02, respectively, were phosphorylated, annealed, and cloned into the pDG458 backbone using golden gate assembly (Table S3).^40^ pDG458 was a gift from Paul Thomas (Cat. No. 100900; Addgene plasmid).

A plasmid donor construct for HDR-based knock-in was generated using a combination of synthesized DNA (Twist Bioscience) of ~800 bp of homology sequence 5′ and 3′ of the point of insertion and DNA sequence encoding TEV-biotin-P2A-mScarlet generated by PCR from existing plasmid. These DNA fragments were then cloned into the pGMC00018 vector (Cat. No. 195320; Addgene plasmid) using two sequential isothermal assembly cloning reactions.^40^ The mScarlet in the donor plasmid was used as a fluorogenic selection markers with cell sorting by FACS.

#### Expression plasmids

A plasmid for expressing the TXNL1 Trx domain (1–112 residues) in the pGEX6P1 vector with a GST tag at its N-terminus followed by PreScission protease cleavage site was synthesized by GenScript Biotech. To express GFP-tagged UBE3C, UBE3C x1 - x84 was in-frame at the N-terminal end of GFP in the pcDNA3.1 vector and synthesized by GenScript Biotech. For the PSMD5 over-expression studies, an HA tag was fused at the N-terminal end of full length PSMD5 (1–504) or PSMD5 truncated at residue 490 (PSMD5ΔC) in pcDNA3.1 by GenScript to generate the HA-PSMD5 and HA-PSMD5ΔC plasmids, respectively. RPN10^UIM1–2^ (203–310), RPN10^UIM1^(196 – 272), RPN2 peptide (940–953) and RPN13 DEUBAD (253–407) were cloned into pGEX6T1 with a GST tag at the N-terminus followed by a PreScission protease cleavage site. RPN13 Pru (1–150) was cloned into the pRSET vector with a 6X-His tag at the N-terminus followed by a PreScission protease cleavage site.

#### Transfection into HCT116 cells

1.5 × 10^5^ HCT116 cells (P3) purchased from American Tissue Culture Collection (CCL-247) were reverse transfected in a 6-well plate (Cat. No. 3506; Costar) with 2 μg of Cas9 plasmid and 5 μg of donor plasmid (RPN11/PSMB4-TEV-Biotin-P2A-mScarlet) by Lipofectamine 3000 (Cat. No. L3000015; Thermo Fisher Scientific, Inc) with Opti-MEM reduced serum medium (Cat. No. 31985070; Life Technologies) according to the manufacturer’s instructions. Transfected cells were incubated at 37°C with 5% CO_2_ humidity for 72 h and then single sorted by FACS as previously described^40^. Briefly, seventy-two hours after transfection, cells were detached with trypsin (Cat no. 25200056; Life Technologies) and resuspended in PBS containing 20% fetal bovine serum. Cells were stained with 4’,6-diamidino-2-phenylindole (DAPI, Cat No. D3571; Thermo Fisher Scientific, Inc) and sorted on a BD FACSymphony S6 Cell Sorter (Waters Biosciences). Debris, doublets, and dead cells were excluded using forward scatter (FSC)-area and side scatter (SSC)-area settings and DAPI staining. Live singlets were analyzed for eGFP and mScarlet expression. Double-positive cells were single-cell sorted into 96-well plates containing 100 μL culture medium. Plates were incubated at 37°C with 5% CO_2_ for 5–6 weeks, with media changes every 2 weeks.

#### PCR genotyping

PCR genotyping was used to confirm integration of the knock-in tag at the genomic level. Different sets of primers (Integrated DNA Technologies, Inc) were designed against different regions of *PSMD14 and PSMB4* as well as the knock-in tag (Table S4). PCR was performed using the Phusion Plus PCR Master mix (Cat. No. F631S; Thermo Fisher Scientific, Inc) and the PCR products were analyzed by Nanopore sequencing.

#### *In situ* photo-crosslinking of CRISPR/Cas9 knock-in cell lines

CRISPR/Cas9 knock-in cells were cultured in 100 mm × 25 mm dishes containing McCoy’s 5A medium (Cat. No. 16600082; Life Technologies Corp.) supplemented with 10 % (v/v) fetal bovine serum (Cat. No A5256701; Thermo Fisher Scientific) at 37°C, 5 % CO_2_. Succinimidyl 4,4′-azipentanoate (SDA) was prepared as a 50 mg mL^−1^ stock in DMSO. Confluent monolayers were rinsed twice with PBS (pH 7.4) and incubated with 4.4 mM SDA (1 mg mL^−1^ in PBS) for 25 min at room temperature in the dark. Excess reagent was quenched by adding Tris-HCl (pH 8.0) to a final concentration of 50 mM and incubating for 5 min. Cells were then washed twice with PBS, overlaid with 5 mL PBS to prevent drying, and irradiated with 365 nm UV light for 10 s using an LED Cube 100 IC chamber paired with an LED Spot 100 HP IC lamp (Hönle UV Technology). After irradiation, cells were scraped, pelleted (200 × g, 5 min, 25°C), snap-frozen in liquid nitrogen, and stored at −80°C for downstream analyses.

#### Sample preparation for pull-down ID mass spectrometry

CRISPR/Cas9 knock-in HCT116 cells (RPN1/RPN11/PSMB4-tagged) were each cultured in 100 mm × 25 mm dishes. For every biological replicate, three dishes per cell line were subjected to the in-situ photo-crosslinking protocol described above, while the remaining three dishes served as non-crosslinked controls. Cells were lysed by Dounce homogenization on ice for 30 min in lysis buffer (50 mM HEPES, pH 7.5, 100 mM NaCl, 1 % NP-40, 10 % glycerol, 5 mM ATP, 10 mM MgCl_2_, protease-inhibitor cocktail; Universal Nuclease, Cat. No. 88700; Thermo Fisher) with gentle inversion every 5 min. Lysates were clarified by centrifugation at 20,000 × g for 30 min at 4°C, and the supernatants were incubated for 2 hours at 4°C with pre-equilibrated MagReSyn Streptavidin MS beads (Cat. No. MR-STP002; ReSyn Biosciences). Beads were washed with wash buffer (50 mM HEPES, pH 7.5, 100 mM NaCl, 10 % glycerol, 2 mM ATP, 5 mM MgCl_2_) four times for 10 min at 4°C with rotation and collected on a magnetic rack.

Bound proteins were denatured in 8 M urea, 100 mM ammonium bicarbonate, and reduced with 5 mM dithiothreitol for 30 min at 37°C, alkylated with 10 mM iodoacetamide for 30 min in the dark, and digested with Lysyl Endopeptidase (enzyme-to-protein ratio 1:100) for 4 h at room temperature with shaking. The urea was then diluted to 1.5 M with 100 mM ammonium bicarbonate and peptides were further digested overnight with trypsin (1:50) at room temperature. Digested peptides were acidified to pH 3, desalted on C18 StageTips, and dried in a vacuum concentrator (Eppendorf) for subsequent LC-MS/MS analysis using data-dependent acquisition (DDA) for label-free quantification (LFQ) method.

#### LFQ data processing

The built-in ‘‘LFQ-MBR’’ workflow in FragPipe v22.0 (https://github.com/Nesvilab/FragPipe) was used for data analysis.^93^ Briefly, MSFragger (v4.1) performed a closed search with initial precursor and fragment mass tolerances set to 20 ppm. Following mass calibration, MSFragger automatically refined these to narrower tolerances. The enzyme was specified as strict trypsin, allowing up to two missed cleavages. Carbamidomethylation of cysteine (+57.0215 Da) was set as a fixed modification, while methionine oxidation (+15.9949 Da), protein N-terminal acetylation (+42.0106 Da), and SDA (and hydrolysed SDA) modifications on lysine residues (+82.04186, +100.05243, +110.04801 Da) were included as variable modifications. After the search, MSBooster was used to calculate deep-learning scores, followed by Percolator for PSM rescoring, and Philosopher for false discovery rate (FDR) estimation at the peptide-spectrum match (PSM) level.^107^ For label-free quantification, IonQuant (v1.10.27) was used with default settings.^108^ The mass tolerance was set to 10 ppm, and the retention time tolerance to 0.4 minutes. Match-between-runs and MaxLFQ intensity calculation were enabled. Additional parameters included ‘‘min scans’’ set to 3, ‘‘min isotopes’’ to 2, and ‘‘MaxLFQ min ions’’ to 2.

#### Data analysis for pull-down ID MS

Label-free quantitative proteomics tables (PulldownID MS) were analyzed with QProMS v2 – ‘‘Quantitative PROteomics Made Simple’’ (https://github.com/ieoresearch/QProMS/). After removal of contaminants, decoys and ‘‘only-identified-by-site’’ entries, proteins were retained if they were quantified in ≥ 80 % of replicates in at least one experimental group. All intensities were log_2_-transformed and variance-stabilizing normalization (VSN) was applied to correct for systematic between-sample effects. Missing values were handled with QProMS’ ‘‘mixed’’ imputation strategy, which treats data missing at random (MAR) and missing not at random (MNAR) separately: MAR values are replaced by the mean of the observed replicates, whereas MNAR values are drawn from a down-shifted Gaussian distribution (down-shift = 1.8 SD; width = 0.3 SD). Differential protein enrichment between conditions was assessed with a two-tailed Welch’s *t* test. *p* values were adjusted for multiple testing with the Benjamini–Hochberg false-discovery-rate (FDR) procedure (‘‘BH truncation’’ in QProMS). Proteins exhibiting an absolute log_2_ fold-change ≥ 2 (i.e., ≥ 4-fold) and FDR-adjusted P < 0.05 were considered significant and visualized in volcano plots generated within QProMS.

All raw and processed Pulldown-ID mass-spectrometry datasets used in this study are available in the PRIDE database under accession code: Pulldown ID-1 (non-crosslinked WT vs RPN1 tag), PXD066281; Pulldown ID-2 (crosslinked WT vs RPN1 tag), PXD066280; Pulldown ID-3 (non-crosslinked WT vs RPN11 tag), PXD066279; Pulldown ID-4 (crosslinked WT vs RPN11 tag), PXD066278; Pulldown ID-5 (non-crosslinked WT vs β4 tag), PXD066268; and Pulldown ID-6 (crosslinked WT vs β4 tag), PXD066284.

#### Sample preparation for co-fractionation MS

Purified proteasome complexes were separated by size-exclusion chromatography on tandem Yarra SEC-4000 columns (3 μm, 300 × 7.8 mm; Phenomenex) equilibrated with 50 mM HEPES (pH 7.5), 100 mM NaCl, 2 % (v/v) glycerol, 1 mM ATP and 2 mM MgCl_2_. A 200 μL sample was injected at a flow rate of 0.4 mL/min. Fractions (200 μL) eluting between 11 mL and 19.8 mL were precipitated with ice-cold acetone (−20°C) and digested sequentially, first with Lys-C and then with trypsin. Peptides were desalted and cleaned using C18 StageTips. The sample from each fraction was further processed to LC-MS/MS.

#### Data-dependent acquisition (DDA) for Label-Free-Quantification (LFQ)

Dried peptides were resuspended in 30 μL of 1.6 % acetonitrile with 0.1 % formic acid, vortexed and sonicated for 1 min before injecting 1 μg of estimated peptide sample onto an Orbitrap Eclipse Mass Spectrometer (Thermo Scientific) coupled to a Vanquish Neo HPLC system. Peptides were ionized using an EASY-Spray source and eluted over an EASY-Spray PepMap Neo 75 μm × 500 mm C18 column with LC–MS quality water or acetonitrile with 0.1% formic acid (mobile A and B, respectively). The flow rate was 0.25 μL/min using a gradient ranging from 1.6 % mobile phase B to 45 % mobile phase B.

Peptides were analyzed using the following MS global parameters: method duration of 120 min; infusion mode, liquid chromatography; expected LC peak widths, 30 s; advanced peak determination checked; default charge state of 2; EASY-IC internal mass calibration; NSI ion source; static spray voltage at 2,000 V in positive mode; static gas mode with a sweep gas setting of 2; ITT temperature of 280°C. Samples were collected using the following shared scan parameters: Duty cycle of 3 s; MS-OT at 120,000 resolution; normal mass range; quadrupole isolation checked; scan range of 400–1,600; RF lens of 35%; a standard AGC target with auto injection time; one microscan in profile mode at positive polarity; EASY-IC checked; subbranch MIPS, peptide; subbranch intensity, 2.5 × 104; subbranch charge state 2–7; subbranch dynamic exclusion of one time after 30 s with a mass tolerance of 5 ppm; exclude within cycle checked; subbranch ddMS2 OT, isolation mode, quadrupole with a 1.4 m/z window; HCD with a fixed, normalized collision energy of 29; 15,000 resolution, normal mass range; auto scan range mode; standard AGC target; one microscan; centroid data.

#### Purification of 26S proteasomes for PhIX-MS

Crosslinked cells were lysed with lysis buffer (50 mM HEPES pH 7.5, 100 mM NaCl, 1% NP-40, 10% glycerol, 5 mM ATP, and 10 mM MgCl_2_, supplemented with protease inhibitor cocktail and the universal nuclease (Cat. No. 88700; ThermoFisher Scientific, Inc)) using a Dounce homogenizer and incubated on ice for 15 min. The lysed sample was centrifuged at 20,000 × g for 30 min in a prechilled centrifuge at 4°C. The supernatant was incubated for 2 h at 4°C with High Capacity Neutravidin Agarose resin (Cat. No. 29202; Thermo Fisher Scientific, Inc) that was preequilibrated with lysis buffer. The resins were next washed with wash buffer (50 mM HEPES pH 7.5, 100 mM NaCl, 10 % glycerol, 1 mM DTT, 2 mM ATP, and 5 mM MgCl_2_) and then incubated for 2 h at 25°C with TEV-digestion buffer (50 mM HEPES pH 7.5, 100 mM NaCl, 10 % glycerol, 1 mM DTT, 2 mM ATP, and 5 mM MgCl_2_) containing His-tagged TEV protease (Cat. No. 12575015; Thermo Fisher Scientific, Inc). To remove TEV enzyme, TALON Superflow resin (Cat. No. 28957502; Cytiva), pre-equilibrated with TEV-digestion buffer, was added to the mixture. The unbound mixture, containing cleaved 26S proteasome, was collected and protein was precipitated with ice-cold acetone.

#### Offline peptide fractionation

For crosslinked peptide enrichment, peptides were fractionated on an Ä KTA Pure system (GE Healthcare) using a Superdex 30 Increase 3.2/300 (GE Healthcare) at a flow rate of 10 μL/min using 30 % (v/v) acetonitrile and 0.1 % (v/v) trifluoroacetic acid as the mobile phase at 4°C. 50 μL fractions were collected from the elution volume 1.00 mL to 1.45 mL and dried for subsequent peptide strong cation exchange (SCX) chromatography or liquid chromatography–tandem mass spectrometry (LC–MS/MS) analysis.

SCX chromatography was performed on the same Ä KTA Pure system with a PolySULFOETHYL A^™^ column (100 mm × 2.1 mm, 3 μm, 300 Å; PolyLC Inc.) at 150 μL/min. Mobile phase A consisted of 10 mM KH_2_PO_4_ (pH 3.0) with 30 % (v/v) acetonitrile; mobile phase B was identical but contained an additional 1 M KCl. After sample loading, the column was washed with 3.5 % B, followed by a linear gradient to 40 % B to elute crosslinked peptides. Fractions of 200 μL were collected, partially dried (to remove acetonitrile), and cleaned on C18 StageTips before being analyzed via LC–MS/MS.

#### LC-MS/MS analysis of crosslinking mass spectrometry

Peptides were analyzed using mass spectrometry global parameters.^109^ High-field asymmetric waveform ion mobility spectrometry (FAIMS) was optionally applied depending on sample injection availability, using compensation voltages of −55, −45/−65, and/or −40/−70 at standard resolution. Targeted precursor selection ranges were also optionally used based on injection number and included the following m/z windows: 380–655, 500–755, 650–905, 750–1005, 900–1800, and 1000–1800. The BoxCar acquisition method was used with a 5.12-second duty cycle, targeted SIM scans acquired at 240,000 resolution, multiplex isolation of 10 ions (user-defined groups), custom AGC target with automatic injection time, and source fragmentation energy set to 10 V. Two BoxCar mass lists were used specifying center m/z values and isolation windows. Box 1: 414.1/30.2, 462.2/24.8, 504.1/23.6, 545.35/23.1, 587.8/25, 634.05/26.7, 686/31.4, 784.45/38.9, 829.65/48.5, 957.2/91.6. Box 2: 439.5/26.6, 483.45/23.7, 524.85/23.9, 566.15/24.3, 610.5/26.4, 658.85/28.9, 715.35/33.3, 786.65/43.5, 882.65/63.5, 1101/202. The AGC target for BoxCar acquisition was set to 50%.

#### Data analysis of PhIX-MS

A recalibration to control for detector error was conducted on MS1 and MS2 based on the median mass-shift of high-confidence (<1% FDR) linear peptide identifications from each raw file. To identify crosslinked peptides, the recalibrated peak lists were searched against the forward (target) and the reversed sequences (as decoys) of crosslinked peptides using the Xi software suite (v.1.8.6; https://github.com/Rappsilber-Laboratory/XiSearch).^90^ The following parameters were applied for the search: MS1 accuracy = 2 ppm; MS2 accuracy = 5 ppm; enzyme = trypsin allowing up to 3 missed cleavages and 2 missing monoisotopic peaks; cross-linker = SDA with an assumed NHS-ester reaction specificity for K, Y, S, T, and protein N-termini; diazirine reaction specificity for A, C, D, E, G, H, I, K, L, P, S, T, V, Y, and protein C- and *N*- termini; fixed modifications = carbamidomethylation on cysteine (Ccm); variable modifications = acetylation on lysine and protein N-termini, oxidation on methionine, hydrolyzed SDA on lysines and protein N-termini. MS cleavage of SDA crosslinks was considered during searches.

Before estimating the false-discovery rate (FDR), the resulting matches were filtered to those having greater than two fragments matched with a non-cleaved SDA and at least five matches total per peptide. These candidates were then filtered to a 2 % residue-pair target-decoy false discovery rate (FDR), with an additional threshold limiting the protein-protein FDR to 5 % using XiFDR, boosting for PPIs (v.2.3.2).^110^

In the RPN1 (datasets 1 and 2) pull-down, we identified 3,213 unique residue pairs (1,010 inter-protein). The RPN11 (dataset 3) pull-down yielded 3,371 unique residue pairs (1,847 inter-protein) at the same FDR threshold. When combined, the two datasets included 5,459 non-redundant crosslinked residue pairs. This includes 2,236 hetero-protein links describing 276 PPIs, with a combined estimated PPI-FDR of 7.7 % (Data S3). In the PSMB4 (dataset 4) pull-down, we identified 1,798 unique residue pairs (955 inter-protein) at the same FDR threshold (Data S3).

The crosslinking MS datasets used in this study are available in the PRIDE database under accession code PXD066071, PXD066061, PXD066021 and PXD066073.

#### AlphaFold structure prediction

We predicted pairwise structural models between the 19 established RP subunits and the 22 proteins identified as directly crosslinked to the RP (Data S5). Predictions were run using AlphaFold2-Multimer (v.2.3.2) with the following settings: 5 models, 3 recycles, templating enabled, and dropout disabled.^105^ All predictions were performed using a local installation of AlphaFold2-Multimer on a Linux server using NVIDIA V100 GPUs.

We built a Python pipeline to automatically extract spatial, confidence, and accuracy metrics, enabling systematic evaluation of predicted protein-protein interactions. Model quality was primarily assessed using predicted TM-score (pTM), which reflects confidence in the structure of individual chains, and interface predicted TM-score (ipTM), which indicates confidence in the predicted inter-chain interface. We used these two scores to evaluate each prediction’s model confidence, which is 0.8*ipTM + 0.2*pTM. Predictions with an average confidence score above 0.65 (across all five models) were considered high confidence.

An AlphaFold3 prediction containing UBE3C, RPN2, RPN3, RPN8, RPN9, RPN10, RPN11, and RPN12 was run via AlphaFold Server^106^ with the default parameters.

#### Purification of 26S proteasomes for cryo-EM

A frozen RPN1-tagged cell pellet (~2 g) from twenty 145 mm × 20 mm cell culture dishes (Cat. No. 639160; Greiner Bio-one Inc.) was homogenized using a Dounce homogenizer (Cat. No. 1234F35; Thomas Scientific Inc.) in 12 mL of lysis buffer (Buffer 1: 50 mM Tris, pH 7.5, 10 % glycerol, 2 mM ATP, 5 mM MgCl_2_ and 1 mM DTT supplemented with Protease inhibitor tablet) followed by 7 freeze-thaw cycles. The lysate was then centrifuged at 17,000 × g for 15 min at 4°C before aliquoting the supernatant, 1 mL each, in 1.5 mL Eppendorf tubes containing 100 μL of buffer equilibrated neutravidin agarose beads (Cat. No. 29204; Thermo Scientific). The bead: supernatant mixture was incubated for 2 h at 4°C before washing with wash buffer (Buffer 2: 50 mM Tris, pH 7.5, 10 % glycerol, 2 mM ATP, 5 mM MgCl_2_ and 1 mM DTT) for 10 min at 4°C. The washing step was repeated three times before incubating each samples in 500 mL TEV cleavage buffer (Buffer 3: 50 mM Tris, pH 7.5, 10 % glycerol, 5 mM ATP, 5 mM MgCl_2_, 1 mM DTT and 15 U mL^−1^ of AcTEV (Cat. No. 12575015; Life Technologies Inc.)) for 2 h at 25°C. After the cleavage the solution containing the cleaved proteasome was separated from beads via centrifugation at 800 × g for 5 min at 4°C. The cleaved proteasome complex was then concentrated using an Amicon filter with 100 kDa molecular weight cut-off (Cat. No. UFC510096; Millipore) to a final concentration of 0.75 mg/mL. The purified proteasome was aliquoted, flash frozen in liquid nitrogen, and stored in −80°C.

#### Cryo-EM sample preparation, grid preparation and data acquisition

To prepare cryo-grids, the purified proteasome samples were thawed on ice for 10–15 min and buffer exchanged with Buffer 4 (50 mM Tris, pH 7.5, 50 mM NaCl, 1.5 mM ATP-γ-S, 5 mM MgCl_2_ and 2 mM DTT) using a Zeba Micro Spin Desalting Columns (7k MWCO, Cat. No. 89883; Thermo Fisher).

Quantifoil grids (R 1.2/1.3 300 mesh, copper, Electron Microscopy Sciences) were glow discharged on each side for 30 s by using a Pelco easiGlow^™^ glow discharge cleaning system at a negative discharge of 25 mA plasma current and 0.38 mbar residual air pressure. The grids were plunge-frozen by using a Leica EM GP2 cryoplunger (Leica Microsystems, Germany) operated at 22°C and 80 % relative humidity. For each grid, 2.5 μL of proteasome sample (0.75 mg/mL) was applied to the carbon side and 1.0 μL of proteasome sample (0.75 mg/mL) was applied to the non-carbon side. The grids were blotted for 2, 2.5, 3, and 4 s at their non-carbon side and vitrified by plunge freezing into liquid ethane cooled by liquid nitrogen. The plunge-frozen grids were then clipped and stored in cryo-grid boxes under liquid nitrogen until the data collection step.

Cryo-EM data was acquired by using a Talos Arctica G2 electron microscope (Thermo Fisher Scientific) equipped with a K3 direct electron detector (Gatan) and an energy filter, operating at 200 kV in super resolution mode (pixel size 0.405 Å/pixel, × 100,000 nominal magnification). 40 frames per movie were acquired for a total dose of approximately 55.6 electrons per Å^2^ and an exposure time of 2.5 s. Data were collected using the EPU program (Thermo Fisher Scientific), with defocus values ranging from −2.0 to −0.8 μm. A total of 27,259 movies were collected from a selected cryo-grid.

#### Cryo-EM image processing

All cryo-EM data processing was performed by using cryoSPARC 4.5.3.^95^ A flowchart of the data processing is displayed in Figures S5A and S5B, and a summary of cryoEM reconstruction statistics is listed in Tables 1and 2. 27,259 dose-fractionated movies were gain-reference corrected, aligned, dose-weighted and summed to single-frame micrographs by patch motion correction, after which constant transfer function (CTF) estimation was done by patch CTF estimation. The micrographs were visually inspected and those with broken or thick ice, or CTF resolution > 5 Å were excluded from the stack, leaving 26,404 micrographs. An initial set of 350,329 particles were blob picked and particles were extracted from 2,330 micrographs, with a box size of 756 pixels and binned by 2x. Templates were created by selecting 9 classes by running 2D classification and template picking identified 254,030 particles from 2,330 micrographs and new templates were created by selecting 46 classes followed by 2D classification. An additional template picked from 6,772 micrographs identified 792,960 particles, for which 2D classifications were performed and new templates created by selecting 23 classes with 184,938 particles. A total of 5,299,947 particles were picked from 26,404 micrographs by template picking and extracted by using a box size of 410 pixels.

Particle stacks were subjected to one round of ab initio model generation and 3D heterogeneous refinement with binning into 3 classes. One class of 1,883,666 particles showed CP density with partial RP density capped on each side (Class 1 in Figure S5A) and was further subjected to one round of ab initio model generation and 3D heterogeneous refinement to remove unfolded particles. 1,199,862 particles were subjected to a heterogeneous refinement into 3 classes, and each class showed RP density on one side or both sides of an CP. Particles from each class were re-extracted by using a box size of 756 pixels, followed by non-uniform refinement and local refinement. To separate particles into individual proteasome states, two masks for RP plus α ring were generated for each class and particles were subjected to alignment-free 3D classification (8 classes, filtered resolution of 15 Å, initialization mode PCA, initial structure low-pass resolution of 30 Å). A total of 48 classes were sorted, pooled based on the conformation of the RP, and particle subtraction applied to remove CP signal beyond the RP-bound α-ring. CTF refinement, reference-based motion correction, non-uniform refinement, and local refinement were next applied. In additional to the 3 classes (105,579 particles) identified as the proteasome ground state (S_A_) and 5 classes (257,779 particles) identified as open-gate states (S_D_), 15 classes with 425,582 particles show extra density near RPN2, RPN11 and Rpn10 (Figure S5A). The heterogeneity of these particles showing extra density was analyzed and clustered in cryoDRGN^96^ followed by re-extracting particles using a box size of 480 pixels. Next, particle subtraction and local refinement were done to yield 26S^TXNL1-OPEN^ and 26S^TXNL1-CLOSED^ maps with overall resolutions of 3.82 Å (143,635 particles) and 4.0 Å (100,351 particles), respectively, as analyzed by the Gold Standard Fourier Shell Correlation (GSFSC) in cryoSPARC (Figures S5A and S5C–S5H).

Class 2 (1,911,783 particles) showed less RP density and no TXNL1 density (Figure S5A) and we therefore focused on class 3 (Figure S5B). After ab initio model generation and heterogeneous refinement (3 classes), one class of open-gate 26S proteasome (S_D_, 424,557 particles), one class of unfold particles (273,512), and one class of free RP (595,212) particles were identified (Figure S5B). Free RP particles were subjected to one more round of heterogeneous refinement, resulting in two classes of free RP particles and one class of RP some CP density visible. The two classes of free RP particles were re-extracted for particles by using a box size of 432 pixels and CTF refinement, reference-based motion correction, non-uniform refinement, and local refinement were performed to generate 19S^TXNL1–1,**Δ**RPT1,**Δ**RPT2^ and 19S^**Δ**TXNL1–1^ maps with overall resolutions of 3.02 Å (228,010 particles) and 3.92 Å (102,897 particles), respectively, as judged by the GSFSC in cryoSPARC (Figure S5B, SI-SN). An alignment-free 3D classification masked on the RPT1-RPT2-RPT5-RPT6 AAA+ domain and RPN1 (6 classes, filtered resolution of 15 Å, initialization mode PCA, initial structure low-pass resolution of 30 Å) of 19S^TXNL1–1,**Δ**RPT1,**Δ**RPT2^ resulted in a combined 3 classes of particles (127,238) with extra density adjacent to RPN1-RPN10-RPN11 or RPT1/RPT2 (Figure S5B). These particles were then subjected to focused local refinement and 3D variability analysis and clustering to generate the 19S^TXNL1–PSMD5^ and 19S^TXNL1–2^ maps with overall resolution of 4.07 Å (54,253 particles, Figures S5O–S5Q) and 3.89 Å (21,325 particles, Figures S5R–S5T), respectively. No additional density was observed near the RPT1-RPT2 in the 19S^TXNL1–2^ map. However, alignment-free 3D classification revealed an extra density near RPT3 in one particle class (Figure S5B). Following local refinement, this subset was reconstructed into the 19S^TXNL1-PSMD10^ map with an overall resolution of 5.35 Å (11,036 particles, Figures S5U–S5W). A parallel process was applied to 19S^**Δ**TXNL1–1^ to generate the 19S^**Δ**TXNL1–2^ map with an overall resolution of 4.24 Å (35,258 particles, Figures S5X–S5Z). Heterogeneous refinement of the class that showed RP with less CP particle density separated the particles of free RP and 26S proteasomes. These free RP particles were then re-extracted by using a box size of 432 pixels and subjected to CTF refinement, reference-based motion correction, non-uniform refinement, and local refinement, generating the 19S^TXNL1–ΔC^ map with an overall resolution of 4.22 Å (39,459 particles, Figures S5AA–S5AC).

The 26S^TXNL1-OPEN^, 26S^TXNL1-CLOSED^, 19S^TXNL1–1,**Δ**RPT1,**Δ**RPT2^, 19S^TXNL1-PSMD5^, 19S^TXNL1–2^, and 19S^TXNL1-ΔC^ maps were then used for further model building and refinement. All GSFSC and viewing direction distribution plots were generated in cryoSPARC, and the local resolution estimation maps were generated in cryoSPARC or the Phenix suite.^100^

#### Model building and structure analyses

RP and CP α subunits from a human 26S proteasome atomic model (PDB: 7W3I) and the TXNL1 PITH domain structure (PDB: 1WWY) were fitted as rigid bodies into the 26S^TXNL1-OPEN^ and 26S^TXNL1-CLOSED^ density maps by using UCSF ChimeraX.^94^ For the 26S^TXNL1-OPEN^ density map, the TXNL1 Trx domain atomic model (PDB: 1GH2) was also fitted as a rigid body into the density. Similarly, RP subunits from PDB: 7W3I and the TXNL1 PITH domain from PDB: 1WWY were fitted as rigid bodies into the 19S^TXNL1-PSMD5^, 19S^TXNL1–2^, and 19S^TXNL1-ΔC^ density maps by using UCSF ChimeraX.^94^ The AlphaFold3 model of PSMD5 with the ATPase ring verified by crosslinking distances was used to fit PSMD5 into the extra density adjacent to RPT1/RPT2 in the 19S^TXNL1-PSMD5^ map. For 19S^TXNL1–1,**Δ**RPT1, **Δ**RPT2^, the RP subunits except RPN1, RPT1, and RPT2 from a proteasome atomic model (PDB: 7W3I) was fitted as a rigid body. To assist model interpretation, the refined maps were processed by EMready^97^ or DeepEMhancer.^72^ The amino acid rotamers and peptide bonds were flipped to increase Ramachandran favorability, decrease rotamer outliers, reduce clashes, and improve fit to the densities by using ISOLDE^98^ and Coot.^99^ The structural models were further refined by iterative cycles of manual building in Coot^99^ and real-space refinement in the Phenix suite.^100^ The geometry and real-space correlation validation were performed by using the phenix.validation_cryoem module in Phenix suite.^100^ A summary of model building and validation statistics is listed in Tables 1 and 2. All low-pass-filtered maps were generated in CryoSPARC with volume tools.^95^

#### Native gel electrophoresis and in-gel peptidase activity

Purified proteasome samples from RPN1-tagged, RPN11-tagged, and PSMB4-tagged cells were checked by in-gel peptidase activity assays following native-PAGE. 2 μg of each proteasome sample was loaded into a native-PAGE gel (3–8% Tris Acetate NuPAGE gel, Cat. No. EA0375; Thermo Fisher Scientific, lnc.) and run for 5.5 h at 150 V at 4°C. The gel was incubated for 15 min at 37°C in in-gel activity assay buffer (50 mM Tris-HCl, pH 7.5, 2 mM ATP, 5 mM MgCl_2_,) with 12.5 μM suc-LLVY-AMC peptide (Cat. No. 10008119; Cayman Chemical Co, Inc.) and then imaged by a Gel Doc EZ Imager (Bio-Rad Laboratories, Inc.). The gel was next incubated again with in-gel activity buffer with addition of 0.02 % SDS and imaged again.

#### Over-expression of UBE3C^N-term^-GFP, HA-PSMD5, and HA-PSMD5ΔC

A 10 cm culture dish was seeded with 1.1 million cells for 18 h, after which 5 μg of UBE3C^N-term^-GFP plasmid was added. For PSMD5 over-expression, RPN1-tagged and PSMB4 -tagged cells were transfected in either a 6-well plate (Cat. No. 3506; Costar) or 10 cm dish using 1 μg or 5 μg of HA-PSMD5 or HA-PSMD5ΔC, respectively. All transfections were performed with Lipofectamine 3000 (Cat. No. L3000015; Thermo Fisher Scientific) in Opti-MEM reduced serum medium (Cat. No. 31985070; Life Technologies), following the manufacturer’s protocol. The cells were incubated at 37°C in a humidified atmosphere containing 5 % CO_2_ for 48 h before harvesting.

#### Pull-down experiment and immunoblotting

The pull-down experiment for RPN1-tagged cells over-expressed without or with UBE3C^N-term^-GFP was performed using Dynabeads MyOne Streptavidin T1 beads (Cat. No. 65601; Life Technologies Corp.). The samples were lysed using lysis buffer (50 mM Tris, pH 7.5, 100 mM NaCl, 1 mM DTT, 5 mM ATP, 5 mM MgCl2, 10 % glycerol, 0.05 % NP-40 and supplemented with protease inhibitor cocktail) and then the lysate was centrifuged to remove cell debris. The total protein amount in the supernatant was measured by Pierce 660 nm protein assay reagent (Cat. No. 22660; Thermo Fisher Scientific) and 1 mg of total protein was incubated with streptavidin beads for 2 h followed by washing 2–3 times with wash buffer (50 mM Tris, pH 7.5, 100 mM NaCl, 1 mM DTT, 5 mM ATP, 5 mM MgCl_2_, and 10 % glycerol). The bound proteasome complex was eluted with addition of 2X SDS loading dye containing 50 mM DTT. Immunoblotting was done by overnight or for a 1 h incubation with primary antibodies (GFP (Cat. No. 2955S; CST), UBE3C (Cat. No. A304–123A; Bethyl Laboratories Inc.), PSMB5 (Cat. No. BML-PW8895; Enzo Life Sciences Inc.), β-actin (Cat. No. 4970T; CST), RPN11 (Cat. No. 4197S; CST), RPN1 (Cat. No. 25430S; CST)) in 5 % skim milk-Tris buffered saline with 0.1 % Tween 20 (TBST), followed by washing 3–4 times for 10 min each, and 1 h incubation with HRP-conjugated anti-rabbit (Cat. No. A16110; Life Techonologies) or anti-mouse (Cat. No. A9917; Sigma) secondary antibodies. The blots were washed again with TBST for 3–4 times (10 min each) before developing them on autoradiography films (Cat. No. 1141J52; Thomas Scientific, Inc) by addition of Pierce ECL chemiluminescent substrate (Cat. No. 32106; Thermo Fisher Scientific, Inc).

The pull-down experiment and LFQ data processing using RPN1-tagged and PSMB4-tagged cells, without or with HA-PSMD5 and HA-PSMD5ΔC over-expression, was performed following the same procedure described for Pulldown ID mass spectrometry (MS) above. Percentage abundance was normalized to the mean MaxLFQ intensity of RP or CP subunits within each replicate using the following equation:

$$Abundance\;\left(\%\right)\;=\;\frac{{\overset{-}{MaxLFQ}}_{CP\;or\;RP,r}}{{\overset{-}{MaxLFQ}}_{RP\;or\;CP,r}}\;\times \;100$$

The raw and processed mass-spectrometry datasets for RPN1 and PSMB4 pull-downs with PSMD5 over-expression are available in the PRIDE database under accession code PXD066535 and PXD076795. The pull-down ID mass spectrometry was performed across three biological replicates, and the data were normalized to the mean RP subunit MaxLFQ intensity within each replicate. The statistical significance was analyzed using Student’s *t* test.

#### Chymotrypsin-like proteasome activity assay

Following transient over-expression, the harvested cells were resuspended in lysis buffer containing 50 mM HEPES (pH 7.5), 100 mM NaCl, 5 % glycerol, 0.1 mM EDTA, 2 mM ATP, and 4 mM MgCl_2_, and lysed by repeated freeze–thaw cycles. Protein concentrations were determined using the Pierce^™^ Dilution-Free^™^ Rapid Gold BCA Protein Assay Kit (Thermo Fisher Scientific), according to the manufacturer’s instructions. Aliquots containing 50 μg of total protein were prepared and adjusted to a final volume of 50 μL with lysis buffer. Proteasome activity was measured using the Proteasome-Glo^™^ Chymotrypsin-Like Assay Kit (Promega), following the manufacturer’s protocol. Luminescence was detected using an Infinite^®^ M200 PRO microplate reader (Tecan). The activity assay was performed across three biological replicates, and the statistical significance was calculated using Student’s *t* test. The whole cell lysates were also immunoprobed with primary antibodies; HA-HRP (Cat. No. 2999S; CST), RPN1 (Cat. No. 25430S; CST), PSMB5 (Cat. No. 12919S; CST), ECM29 (Cat. No. AB28666; Abcam), β-actin (Cat. No. AB8226; Abcam) in Pierce^™^ Protein-Free T20 (TBS) Blocking Buffer (Cat. No. 37571; Thermo Fisher Scientific). After three 10-min washes in TBST, blots were incubated for 1 h with HRP-conjugated anti-rabbit (Cat. No. 31460; Life Techonologies) or anti-mouse (Cat. No. 31430; Life Techonologies) secondary antibodies, washed again, and developed on a ChemiDoc imaging system (Bio-Rad) using SuperSignal West Pico PLUS substrate (Cat. No. 34577; Thermo Fisher Scientific, Inc).

#### Protein expression and purification

All the bacterial expression plasmids were expressed in *Escherichia coli* strain BL21(DE3) cells (Cat. no. EC0114; Thermo Fisher Scientific, lnc) expect for RPN13 Pru and GST-RPN2 (940–953), for which BL21(DE3) pLysS cells (Cat. No. C606003; Life Technologies Corp.) were used. The cells were grown at 37 °C to optical density at 600 nm of 0.5–0.6 and induced for protein expression by addition of 0.5 mM of isopropyl-1-thio-β-D-thiogalactopyranoside (IPTG, Cat. No. P101010; UBPBio). Post induction, the cells were grown at 17°C for 18 h before harvesting them via centrifugation at 4,550 × g for 30 min at 4°C. The cells were lysed by sonication, and cellular debris was removed by centrifugation at 31,000 × g for 30 min. The supernatant was incubated either with glutathione S-sepharose 4B resin (Cat. no. GE17075605; Millipore Sigma) or Talon Metal Affinity resin (Cat. No. 635502; Takara Bio Inc.) for 2 h. GST-RPN10^UIM1–2^, GST-RPN10^UIM1^ and GST-RPN2 (940–953) were eluted from the resin using 20 mM of reduced glutathione (Cat. No. G4251; Sigma) in 20 mM Tris (pH 7.5), 300 mM NaCl, and 10 mM β-mercaptoethanol (βME). TXNL1 Trx, RPN13 DEUBAD, and RPN13 Pru were eluted from the glutathione and Talon resin by overnight incubation with 50 units of PreScission protease (Cat. No. 27084301; Cytivia Life Sciences) in 50 mM Tis buffer (pH 7.5), 100 mM NaCl and 1 mM DTT. The eluted proteins were subjected to size exclusion chromatography with a HiLoad 16/600 Superdex 75 pg column on an FPLC system for further purification. ^15^N ammonium chloride was used for isotopic labeling of RPN13 Pru and RPN13 DEUBAD proteins.

#### NMR experiment

Aliquots of ^15^N-labeled RPN13 Pru, ^15^N-labeled RPN13 DEUBAD, and unlabeled TXNL1 Trx were dialyzed using Slide-A-Lyzer mini dialysis devices into identical NMR buffer (20 mM sodium phosphate, 50 mM NaCl, pH 6.0) without any reducing reagent. For the oxidation of ^15^N-labeled RPN13 Pru and ^15^N-labeled RPN13 DEUBAD, the proteins were incubated with 0.05% H_2_O_2_ on ice for 10 min and the final round of buffer exchange into NMR buffer was done to remove remaining H_2_O_2_ from the samples. For the reduced ^15^N-labeled RPN13 Pru and ^15^N-labeled RPN13 DEUBAD samples, the purified proteins were buffer exchanged into NMR buffer containing 1 mM DTT.

2D ^1^H,^15^N HSQC spectra were recoded for 20 μM of ^15^N-labeled RPN13 Pru preincubated with equimolar unlabeled RPN2 peptide, and next with 5-fold, 10-fold, or 15-fold molar excess unlabeled TXNL1 Trx. ^1^H,^15^N HSQC was also recorded for 22 μM of reduced or oxidized ^15^N-labeled RPN13 Pru, and 22 μM of oxidized ^15^N-labeled RPN13 Pru with 4-fold excess of TXNL1 Trx (88 μM). ^1^H,^15^N HSQC spectra was also recorded for 25 μM of reduced or oxidized ^15^N-labeled RPN13 DEUBAD, and 25 μM of oxidized ^15^N-labeled RPN13 DEUBAD with 4-fold excess of TXNL1 Trx (100 μM). All of the NMR experiments were recorded at 10°C on a Bruker Advance 600 MHz or 900 MHz spectrometer. The data were processed by NMRPipe^101^ (version 11.5) and spectra analyzed and visualized with XEASY^102^ or NMRFAM-SPARKY^103^ (version 3.190) by using NMRbox.^104^

#### ITC experiment

For the ITC experiments, RPN13 Pru and TXNL1 Trx proteins were dialyzed utilizing a Slide-A-Lyzer mini dialysis device either in reducing ITC buffer (20 mM sodium phosphate, 50 mM NaCl, 10 mM βME, pH 6.0) or in non-reducing ITC buffer (20 mM sodium phosphate, 50 mM NaCl, pH 6.0). For the TXNL1 Trx-treated RPN13 Pru sample, RPN13 Pru was first oxidized as described above and then incubated with 3-fold molar excess of TXNL1 Trx for 2 h and then removed from the sample by size exclusion chromatography. All the ITC experiments were performed at 25 °C on a MicroCal iTC200 system (Malvern, PA, USA), with RPN13 Pru in the cell and RPN2 (940–953) in the syringe. One aliquot of 0.5 μL followed by 18 aliquots of 2.1 μL of 156 μM, 205.7 μM, or 160 μM RPN2 peptide was injected at 1,000 r.p.m. into the calorimeter cell (volume 200.7 μL), which contained either 15.6 μM (reduced), 20.57 μM (oxidized), or 16 μM (TXNL1 Trx-treated oxidized) RPN13 Pru in reducing or non-reducing conditions. Blank experiments were performed by replacing protein samples with buffer and the resulting data subtracted from the experimental data during analyses. The integrated interaction heat values were normalized as a function of protein concentration, and the data were fit with MicroCal Origin 7.0 software. Binding was assumed to be at one site to yield a binding affinity K_a_ (1/K_d_), stoichiometry and other thermodynamic parameters.

#### UBE3C pull-down experiment

8 nmoles of GST, GST-RPN10^UIM1–2^ and GST-RPN10^UIM1^ were bound to 25 μL slurry of glutathione S-sepharose 4B resins for 1 hour at 4°C. The beads were spun down (300 × g) and washed 2–3 times to remove the unbound excess proteins before incubating them with 1 mg of cell lysate from HCT116 cells, lysed in lysis buffer (50 mM TrisHCl (pH 7.5), 150 mM NaCl, 1 mM phenylmethylsulfonyl fluoride (PMSF, Cat. No. 10837091001; Sigma) and 1% Triton-X supplemented with EDTA-free protease inhibitor cocktail (Cat. No. A32955; Thermo Fisher Scientific, lnc.) for 3 h at 4°C. The beads were again spun down and washed with lysis buffer before eluting the sample by adding 37.5 μL of 3X LDS loading dye to each sample. The samples were heated at 72°C before loading them on an SDS gel and then transferred onto a PVDF membrane. Before immunoblotting with UBE3C antibodies, the PVDF membrane was stained with Ponceau stain to check the recombinant proteins amount on the membrane.

#### Pull-down of proteasomes using biotinylated MC1 against RPN1

Harvested RPN1-tagged or RPN10^VWA^ cells were lysed in lysis buffer containing Tris (50 mM, pH 7.5), DTT (1mM), glycerol (10%), ATP (2.5mM) and MgCl_2_ (5 mM) with protease inhibitor cocktail. 700 μg of total protein was incubated without or with biotinylated MC1 (20 μM).^55^ Following 60 minutes of incubation, 50 μL of DynabeadsTM Streptavidin T1 slurry was added and the beads washed three times. The samples were eluted from the beads using 4X LDS loading dye. The pull-down samples along with the lysate samples were immunoprobed with UBE3C, Rpn10, or β-actin antibodies.

### QUANTIFICATION AND STATISTICAL ANALYSIS

Statistical details for experiments subjected to statistical analysis are provided in the corresponding figure legends. Statistical analysis for pull down ID mass spectrometry and chymotrypsin-like proteasome activity assay was done using Student’s *t* test across three biological replicates. Statistical analyses were performed using custom Python scripts employing the SciPy library.

## Supplementary Material

Figures S1–S9 and Tables S1–S4

Video S1

Video S2

Video S3

Data S1

Data S2

Data S5

Data S6

Data S7

Data S8

Data S4

Data S3

SUPPLEMENTAL INFORMATION

Supplemental information can be found online at https://doi.org/10.1016/j.molcel.2026.06.032.

## Figures and Tables

**Table 1.** Statistics for cryo-EM analyses of 26S^TXNL1-OPEN^ and 26S^TXNL1-CLOSED^, related to Figures 3 and 4

Proteasome complex	26^STXNL1-OPEN^	26^STXNL1-CLOSED^
Data collection and image processing		
Microscope	Talos Arctica G2	
Camera	K3	
Magnification	100,000	
Voltage (kV)	200	
Electron exposure (e^−^/Å^2^)	55.6	
Defocus range (μm)	0.8–2.0	
Pixel size (Å)	0.81	
Symmetry imposed	C1	
Total micrographs	27,259	
Initial particle images	5,299,947	
Final particle images	143,635	100,351
Map resolution (Å)	3.82	4.0
FSC threshold	0.143	0.143
Map sharpening *B* factor (Å^2^)	−58.0	−41.8
EMDB code	EMD-71740	EMD-71741
Model building and refinement		
Initial model used (PDB ID)	7W3I, 1WWY, 1GH2	7W3I, 1WWY
Model resolution (Å)	4.43	4.40
FSC threshold	0.143	0.143
Model composition		
Non-hydrogen atoms	71,049	69,593
Amino acid residues	8,972	8,790
Protein molecules	26	26
Real-space correlation		
CC (volume)	0.58	0.70
CC (mask)	0.61	0.73
Root mean square (RMS) deviations		
Bond lengths (Å)	0.007	0.007
Bond angles (°)	1.388	1.468
Validation		
MolProbity score	1.77	2.12
Clash score	6.46	7.82
Rotamers outliers (%)	0.41	1.86
CaBLAM outliers (%)	4.30	5.27
Cβ outliers (%)	0.04	0.01
Ramachandran plot		
Favored (%)	93.54	92.07
Allowed (%)	6.46	7.93
Outliers (%)	0.00	0.00
PDB code	9PMO	9PMQ

FSC, Fourier shell correlation; CaBLAM, C-alpha-based low-resolution annotation method.

**Table 2.** Statistics for cryo-EM analyses of 19S^TXNL1-1,ΔRPT1 ΔRPT2^, 19S^TXNL1-PSMD5^, 19S^TXNL1-2^, 19S^TXNL1-ΔC^, 19S^TXNL1-PSMD10^, 19S^ΔTXNL1-1^, and 19S^ΔTXNL1-2^, related to Figure 6

Proteasome complex	19S^TXNL1-1,ΔRPT1 ΔRPT2^	19S^TXNL1-PSMD5^	19S^TXNL1-2^	19S^TXNL1-ΔC^
Data collection and image processing				
Microscope	Talos Arctica G2			
Camera	K3			
Magnification	100,000			
Voltage (kV)	200			
Electron exposure (e^−^/Å^2^)	55.6			
Defocus range (μm)	0.8–2.0			
Pixel size (Å)	0.81			
Symmetry imposed	C1			
Total micrographs	27,259			
Initial particle images	5,299,947			
Final particle images	228,010	54,253	21,325	39,459
Map resolution (Å)	3.02	4.07	3.89	4.22
FSC threshold	0.143	0.143	0.143	0.143
Map sharpening *B* factor (Å^2^)	−88.9	−89.0	−51.0	−47.3
EMDB code	EMD-71813	EMD-71810	EMD-76283	EMD-71737
Model building and refinement				
Initial model used (PDB ID)	7W3I, 1WWY	7W3I, 1WWY	7W3I, 1WWY	7W3I, 1WWY
Model resolution (Å)	3.85	4.24	3.91	4.24
FSC threshold	0.143	0.143	0.143	0.143
Model composition				
Non-hydrogen atoms	41,598	57,766	53,094	54,953
Amino acid residues	5,206	7,267	6,678	6,903
Protein molecules	16	20	19	19
Real-space correlation				
CC (volume)	0.51	0.63	0.62	0.64
CC (mask)	0.52	0.66	0.64	0.67
RMS deviations				
Bond lengths (Å)	0.006	0.007	0.008	0.009
Bond angles (°)	1.310	1.336	1.533	1.561
Validation				
MolProbity score	1.64	1.83	2.20	2.22
Clash score	5.45	6.39	6.19	9.27
Rotamers outliers (%)	0.26	0.52	3.74	1.61
CaBLAM outliers (%)	2.89	4.69	3.72	6.17
Cβ outliers (%)	0.00	0.00	0.05	0.00
Ramachandran plot				
Favored (%)	94.94	92.33	93.48	89.32
Allowed (%)	5.06	7.67	6.52	10.68
Outliers (%)	0.00	0.00	0.00	0.00
PDB code	9PRT	9PRO	12BM	9PMJ

Proteasome complex	19S^TXNL1-PSMD10^	19S^ΔTXNL1-1^		19S^ΔTXNL1-2^
Data collection and image processing				
Microscope	Talos Arctica G2			
Camera	K3			
Magnification	100,000			
Voltage (kV)	200			
Electron exposure (e^−^/Å^2^)	55.6			
Defocus range (μm)	0.8–2.0			
Pixel size (Å)	0.81			
Symmetry imposed	C1			
Total micrographs	27,259			
Initial particle images	5,299,947			
Final particle images	11,036	102,897		35,258
Map resolution (Å)	5.35	3.92		4.24
FSC threshold	0.143	0.143		0.143
Map sharpening *B* factor (Å^2^)	−51.0	−57.5		−85.2
EMDB code	EMD-76415	EMD-71791		EMD-71795

**KEY RESOURCES TABLE:** 

REAGENT or RESOURCE	SOURCE	IDENTIFIER
Antibodies		
GFP	Cell signaling technology	cat#2955S; RRID: AB_2255011
UBE3C	Bethyl Laboratories Inc.	cat#A304-123A
PSMB5	Enzo Life Sciences Inc.	cat#BML-PW8895; RRID:AB_10540901
PSMB5	Cell signaling technology	cat#12919S; RRID:AB_2798061
β-actin	Cell signaling technology	cat#4970T; RRID:AB_2223172
β-actin	Abcam	cat#AB8226; RRID:AB_306371
RPN11	Cell signaling technology	cat#4197S; RRID:AB_11178935
RPN1	Cell signaling technology	cat#25430S; RRID:AB_2798903
RPN10	Cell signaling technology	cat#3336S; RRID:AB_11178520
HRP conjugated anti-rabbit	Life Technologies	cat#A16110; RRID:AB_2534782
HRP conjugated anti-rabbit	Life Technologies	cat#31460; RRID:AB_228341
HRP conjugated anti-mouse	Sigma Aldrich	cat#A9917; RRID:AB_258476
HRP conjugated anti-mouse	Life Technologies	cat#31430; RRID:AB_228307
HA-HRP	Cell signaling technology	cat#2999S; RRID:AB_1264166
ECM29	Abcam	cat#AB28666; RRID:AB_732066
Bacterial and virus strains		
Escherichia coli strain BL21(DE3) cells	Life Technologies	cat#EC0114
Escherichia coli strain BL21(DE3) pLysS cells	Life Technologies	cat#C606003
Chemicals, peptides, and recombinant proteins		
Lipofectamine 3000	Thermo Fisher Scientific	cat#L3000015
Opti-MEM reduced serum medium	Life Technologies	cat#31985070
McCoy’s 5A (modified) medium	Thermo Fisher Scientific	cat#16600082
DMEM medium	Thermo Fisher Scientific	cat#10566016
Fetal bovine serum	Thermo Fisher Scientific	cat#A5209402
Succinimidyl 4,4′-azipentanoate (SDA)	Thermo Fisher Scientific	cat#26167
Dimethylsulfoxide (DMSO)	Sigma-Aldrich	cat#276855-12X100ML
Phosphate-buffered saline (PBS)	Research Products International Corp	cat#P32060
Tris-HCl	Millipore Sigma	cat#10812846001
HEPES	Sigma-Aldrich	cat#H4034
Sodium chloride	Sigma-Aldrich	cat#S9888
NP-40	EMD Millipore Corp	cat#492018
Glycerol	Sigma-Aldrich	cat#49767
Adenosine 5′-triphosphate disodium salt hydrate (ATP)	Sigma-Aldrich	cat#A2383
Magnesium chloride hexahydrate	EMD Millipore Corp	cat#442615
Urea	Sigma-Aldrich	cat#51456
Ammonium bicarbonate	Sigma-Aldrich	cat#A6141
Dithiothreitol	Sigma-Aldrich	cat#43815
Iodoacetamide	Sigma-Aldrich	cat#I1149
Lysyl Endopeptidase (Lys-C)	Fujifilm Wako Chemicals USA Corporation	cat#125-05061
Trypsin (MS grade)	Thermo Fisher Scientific	cat#90057
Empore C18 SPE Disks	Millipore Sigma	cat#66883-U
Protease Inhibitor Cocktail	Roche	cat#11697498001
Pierce Universal Nuclease for Cell Lysis	Thermo Fisher Scientific	cat#88700
MagReSyn Streptavidin MS beads	ReSyn Biosciences	cat#MR-STP002
Acetonitrile (LC-MS grade)	Fisher Scientific	cat#047138.K7
Formic acid (LC-MS grade)	VWR	cat#PI85178
EASY-Spray PepMap Neo 75 μm × 500 mm C18 column	Thermo Fisher Scientific	cat#ES75500PN
High Capacity Neutravidin Agarose resin	Thermo Fisher Scientific	cat#29202
TEV protease	Thermo Fisher Scientific	cat#12575015
TALON Superflow resin	Cytiva	cat#28957502
Trifluoroacetic acid (MS grade)	Fisher Scientific	cat#PI85183
Potassium phosphate monobasic	Sigma-Aldrich	cat#P0662
Potassium chloride	Sigma-Aldrich	cat#P3911
Acetone (HPLC)	Sigma-Aldrich	cat#270725
AttractSPE^®^Disks Bio – C18 disk for stage-tip	AFFINISEP	cat#SPE-Disks-Bio-C18-100.47.20
Adenosine 5′-[γ-thio]triphosphate tetralithium salt (ATP-γ-S)	Sigma-Aldrich	cat#A1388
Dynabeads MyOne Streptavidin T1 beads	Life Technologies	cat#65601
Pierce^™^ Protein-Free T20 (TBS) Blocking Buffer	Thermo Fisher Scientific	cat#37571
Isopropyl-1-thio-β-D-thiogalactopyranoside (IPTG)	UBPBio	cat#P101010
Glutathione S-sepharose 4B resins	Millipore Sigma	cat#GE17075605
Talon Metal Affinity resin	Sigma-Aldrich	cat#G4251
PreScission protease	Cytivia Life Sciences	cat#27084301
Phenylmethylsulfonyl fluride (PMSF)	Sigma-Aldrich	cat#10837091001
Triton X-100	Sigma-Aldrich	cat#X100
4’,6-diamidino-2-phenylindole (DAPI)	Thermo Fisher Scientific	cat#D3571
Trypsin (cell culture grade)	Life Technologies	cat#25200056
Critical commercial assays		
Phusion Plus PCR Master Mix	Thermo Fisher Scientific	cat#F631S
Pierce^™^ Dilution-Free^™^ Rapid Gold BCA Protein Assay Kit	Thermo Fisher Scientific	cat#A55860
Proteasome-Glo^™^ Chymotrypsin-Like Assay Kit	Promega	cat#G8621
suc-LLVY-AMC peptide	Cayman Chemical Co Inc.	cat#10008119
Pierce 660 nm protein assay reagent	Thermo Fisher Scientific	cat#22660
SuperSignal West Pico PLUS substrate	Thermo Fisher Scientific	cat#34577
Pierce ECL chemiluminescent substrate	Thermo Fisher Scientific	cat#32106
Deposited data		
Pull-down ID-1 (non-crosslinked WT vs RPN1 tag) MS data	This manuscript	PXD066281
Pull-down ID-2 (crosslinked WT vs RPN1 tag) MS data	This manuscript	PXD066280
Pull-down ID-3 (non-crosslinked WT vs RPN11 tag) MS data	This manuscript	PXD066279
Pull-down ID-4 (crosslinked WT vs RPN11 tag) MS data	This manuscript	PXD066278
Pull-down ID-5 (non-crosslinked WT vs PSMB4 tag) MS data	This manuscript	PXD066268
Pull-down ID-6 (crosslinked WT vs PSMB4 tag) MS data	This manuscript	PXD066284
Co-fractionation MS data	This manuscript	PXD066287
Crosslinking MS data	This manuscript	PXD066071, PXD066061, PXD066021 and PXD066073
Pull-down ID (RPN1 with HA-PSMD5 overexpression) MS data	This manuscript	PXD066535
Pull-down ID (PSMB4 with HA-PSMD5 overexpression) MS data	This manuscript	PXD076795
Cryo-EM density map of the 26S^TXNL1-OPEN^ structure	This manuscript	EMDB: EMD-71740
Coordinates of the 26S^TXNL1-OPEN^ structure	This manuscript	PDB: 9PMO
Cryo-EM density map of the 26S^TXNL1-CLOSED^ structure	This manuscript	EMDB: EMD-71741
Coordinates of the 26S^TXNL1-CLOSED^ structure	This manuscript	PDB: 9PMQ
Cryo-EM density map of the 19S^ΔTXNL1-1,ΔRPT1,ΔRPT2^ structure	This manuscript	EMDB: EMD-71813
Coordinates of the 19S^TXNL1-1,ΔRPT1,ΔRPT2^ structure	This manuscript	PDB: 9PRT
Cryo-EM density map of the 19S^TXNL1-PSMD5^ structure	This manuscript	EMDB: EMD-71810
Coordinates of the 19S^TXNL1-PSMD5^ structure	This manuscript	PDB: 9PRO
Cryo-EM density map of the 19S^TXNL1-2^ structure	This manuscript	EMDB: EMD-76283
Coordinates of the 19S^TXNL1-2^ structure	This manuscript	PDB: 12BM
Cryo-EM density map of the 19S^TXNL1-ΔC^ structure	This manuscript	EMDB: EMD-71737
Coordinates of the 19S^TXNL1-ΔC^ structure	This manuscript	PDB: 9PMJ
Cryo-EM density map of the 19S^TXNL1-PSMD10^ structure	This manuscript	EMDB: EMD-76415
Cryo-EM density map of the 19S^ΔTXNL1-1^ structure	This manuscript	EMDB: EMD-71791
Cryo-EM density map of the 19S^ΔTXNL1-2^ structure	This manuscript	EMDB: EMD-71795
Raw micrographs	This manuscript	EMPIAR: EMPIAR-12891
Raw immunoblots	This manuscript	Mendeley Data: http://www.doi.org/10.17632/zcpm9k38yr.1
Coordinates of the USP14 bound 26S proteasome structure	Zhang et al.^46^	PDB: 7W3M, 7W3K, 7W3J, 7W3I, 7W3H, 7W3G, 7W3F, 7W3C, 7W3B, 7W3A, 7W39, 7W38, 7W37
Coordinates of the 26S proteasome structure	Dong et al.^86^	PDB: 6MSK, 6MSJ, 6MSH, 6MSG, 6MSE, 6MSD, 6MSB
Coordinates of the PA28α-β bound Immunoproteasome structure	Chen et al.^45^	PBD: 7DR6
Coordinates of the K11/K48-branched ubiquitin chain	Draczkowski et al.^87^	PDB: 8JTI
Coordinates of the RPN13, UCHL5, and ubiquitin complex	Sahtoe et al.^48^	PDB: 4UEL
Coordinates of the p28-bound 19S RP structure	Lu et al.^71^	PDB: 5VHI
Cryo-EM density map of the p28-bound 19S RP structure	Lu et al.^71^	EMDB: EMD-8676
Coordinates of the TXNL1 bound 26S proteasome structure	Arkinson et al.^58^	PDB: 9E8K, 9E8O, 9E8H, 9E8G,
Experimental models: Cell lines		
HCT116	ATCC	cat#CCL-247
RPN10VWA	Lu et al.^56^	N/A
HEK293T	ATCC	cat#CRL-3216
Oligonucleotides		
Guide RNAs tested in 293T cells	Integrated DNA Technologies	Table S1
Oligos for guide RNAs	Integrated DNA Technologies	Table S3
Primers used in this study	Integrated DNA Technologies	Table S4
Recombinant DNA		
pJT0059_100901_PSMB4-Cterm-JT-IVT-44-48	This manuscript	N/A
pJT0069_PSMB4-Cterm-Biotin-mScarlet	This manuscript	N/A
pMC0437_SpCas9-2A-Puro_PSMD14-Cterm-IVT-2995-IVT-2997	This manuscript	N/A
pMG0826_PSMD14-Cterm-Biotin-2A-mScarlet	This manuscript	N/A
pDG458	Adikusuma et al.^88^	Addgene #100900; RRID:Addgene_100900
pGMC00018	A gift from Raj Chari	Addgene #195320; RRID:Addgene_195320
pX458	Ran et al.^89^	Addgene #48138; RRID:Addgene_48138
Software and algorithms		
AlphaFold2 prediction analysis Python pipeline	This manuscript	Zenodo Data: http://www.doi.org/10.5281/zenodo.20708496
Custom Python scripts for statistical analysis	This manuscript	Zenodo Data: http://www.doi.org/10.5281/zenodo.20707940
xiSearch (v.1.8.6)	Mendes et al.^90^	https://www.rappsilberlab.org/software/xisearch/
xiFDR (v.2.3.2)	Mendes et al.^90^	https://www.rappsilberlab.org/software/xifdr/
xiView	Combe et al.^91^	https://www.rappsilberlab.org/software/xiview/
sgRNA Scorer 2.0	Chari et al.^92^	https://frederick.cancer.gov/resources/repositories/sgrnascorer
FragPipe (v.22)	Github	https://github.com/Nesvilab/FragPipe
MSFragger (v.4.1)	Kong et al.^93^	https://github.com/Nesvilab/MSFragger
QProMS (v.2)	Github	https://github.com/FabioBedin/QProMS
ChimeraX (v.1.9)	Pettersen et al.^94^	https://www.cgl.ucsf.edu/chimerax/download.html
EPU program (v.3.3.0)	Thermo Fisher Scientific	https://www.thermofisher.com/us/en/home/electron-microscopy/products/software-em-3d-vis/epu-software.html
cryoSPARC (v.4.5.3)	Punjani et al.^95^	https://cryosparc.com/
cryoDRGN (v.3.4.2)	Zhong et al.^96^	https://github.com/ml-struct-bio/cryodrgn
Emready (v.2.0)	He et al.^97^	http://huanglab.phys.hust.edu.cn/EMReady/
DeepEMhancer (v.0.15)	Sanchez-Garcia et al.^72^	https://github.com/rsanchezgarc/deepEMhancer
ISOLDE (v.1.9)	Croll^98^	https://tristanic.github.io/isolde/index.html
Coot (v.0.9.8.93 EL)	Emsley et al.^99^	https://www2.mrc-lmb.cam.ac.uk/personal/pemsley/coot/
Phenix (v.1.21.2)	Liebschner et al.^100^	https://phenix-online.org/
NMRPipe (v.11.5)	Delaglio et al.^101^	https://spin.niddk.nih.gov/bax-apps/NMRPipe/info.html
XEASY (v.1.3.11)	Bartels et al.^102^	
NMRFAM-SPARKY (v.3.190)	Lee et al.^103^	https://nmrfam.wisc.edu/nmrfam-sparky-distribution/
NMRbox	Maciejewski et al.^104^	https://nmrbox.nmrhub.org/
AlphaFold 2 (v.2.3.2)	Evans et al.^105^	https://github.com/google-deepmind/alphafold
AlphaFold 3	Abramson et al.^106^	https://alphafoldserver.com/
MicroCal Origin (v.7.0)	Microcal Software, Inc.	N/A
Other		
BD FACSymphony S6 Cell Sorter	Waters Biosciences	https://www.bdbiosciences.com/en-us/products/instruments/flow-cytometers/research-cell-sorters/bd-facsymphony-s6?utm_source=chatgpt.com
LED Cube 100 IC	Hönle UV Technology	https://www.hoenle.com/products/uv-equipment-uv-systems-uv-units/uvacube-led-cube/led-cube-100-ic
Yarra SEC-4000 (3μm, 300x7.8mm) column	Phenomenex	cat#00H-4514-K0
AKTA pure micro	Cytivia Life Sciences	cat#29302479
Superdex 30 Increase 3.2/300 column	Cytivia Life Sciences	cat#29219758
PolySULFOETHYL A^™^ column (100 mm × 2.1 mm, 3 μm, 300 Å)	PolyLC Inc.	cat#102SE0303
Infinite^®^ M200 PRO microplate reader	Tecan	https://lifesciences.tecan.com/plate_readers/infinite_200_pro
Dounce homogenizer	Thomas Scientific Inc.	cat#1234F35
3-8% Tris Acetate NuPAGE gel	Thermo Fisher Scientific	cat#EA0375
Zeba Micro Spin Desalting Column (7k MWCO)	Thermo Fisher Scientific	cat#89883
Quantifoil grids (R 1.2/1.3 300 mesh, copper)	Electron Microscopy Sciences	cat#Q350CR1.3
Pelco easiGlow^™^ glow discharge cleaning system	Ted Pella, Inc.	https://www.tedpella.com/easiGlow_html/Glow-Discharge-Cleaning-System.aspx
Leica EM GP2 cryoplunger	Leica Microsystems	https://www.leica-microsystems.com/products/sample-preparation-for-electron-microscopy/p/leica-em-gp2/
Talos Arctica G2 electron microscope	Thermo Fisher Scientific	https://www.thermofisher.com/us/en/home/electron-microscopy/products/transmission-electron-microscopes/glacios-cryo-tem.html?SID=srch-srp-TALOSARCTICA
ChemiDoc imaging system	BIO RAD	https://www.bio-rad.com/en-us/product/chemidoc-imaging-system?ID=OI91XQ15
HiLoad 16/600 Superdex 75 pg column	Cytivia Life Sciences	cat#28989333
Slide-A-Lyzer mini dialysis	Thermo Fisher Scientific	cat#88403
MicroCal iTC200 system	Malvern	https://www.malvernpanalytical.com/en/support/product-support/microcal-range/microcal-itc-range/microcal-itc200
Autoradiography films	Thomas Scientific Inc	cat#1141J52

## Highlights

- PhIX-MS provides residue-level contacts for transient interactions *in situ*
- Cryo-EM and PhIX-MS define PSMD5-RP structures with tail insertion into the RP ATPase
- The TXNL1–26S structure with dynamic Trx is viewed by cryo-EM and supported by PhIX-MS
- An AI-based model and *in situ* crosslinks place UBE3C along the upper RP rim
