# Supplementary material for: Structures of dynamic interactors at native proteasomes by PhIX-MS and cryo-electron microscopy
Source: Mol Cell. Author manuscript; Available in PMC 2026 Sep 16. (PMC13580404; doi:10.1016/j.molcel.2026.06.032)
Supplement: Supplementary file 1 [file NIHMS2194827-supplement-Figures_S1_S9_and_Tables_S1_S4.pdf]

upplemental information

Structures of dynamic interactors at native proteasomes by PhIX-MS and cryo-electron microscopy

Kitaik Lee[1,6], Hitendra Negi[2,6], Xiang Chen[2,6], Katerina Atallah-Yunes[1], Sophia Truslow[2], Rithik E. Castelino[2], Mary R. Guest[3], Anthony M. Ciancone[1], Xiuxiu Lu[2], Sergey G. Tarasov[4], Raj Chari[3], Kylie J. Walters[2,7,8], Francis J. O'Reilly[1,5,7]

[1]Structural System Biology Section, Center for Structural Biology, Center for Cancer Research, National Cancer Institute (NCI), National Institutes of Health, Frederick, MD 21702-1201, USA.

[2]Protein Processing Section, Center for Structural Biology, Center for Cancer Research, National Cancer Institute (NCI), National Institutes of Health, Frederick, MD 21702-1201, USA.

[3]Genome Modification Core, Laboratory Animal Sciences Program, Frederick National Laboratory for Cancer Research, Frederick, MD 21702-1201, USA.

[4]Biophysics Resource, Center for Structural Biology, Center for Cancer Research, National Cancer Institute (NCI), National Institutes of Health, Frederick, MD 21702-1201, USA.

[5]Center of Excellence for Data Driven Discovery, Department of Structural Biology, St Jude Children's Research Hospital, Memphis, Tennessee 38105, United States

[6]These authors contributed equally

[7]Senior Author

[8]Lead contact: kylie.walters@nih.gov

Inventory

Table S1-S4

Figure S1-S9

Table S1. Guide RNAs tested in 293T cells, related to Figure 1.

Name	Gene	Description	Target
IVT-2994	PSMD14	PSMD14-Cterm_FASTA:75-97	TTAGGACCCCAAACGTCATTTGG
IVT-2995*	PSMD14	PSMD14-Cterm_FASTA:81-103	GTTCCTCCAAATGACGTTTGGGG
IVT-2996	PSMD14	PSMD14-Cterm_FASTA:83-105	ATGTTCCTCCAAATGACGTTTGG
IVT-2997*	PSMD14	PSMD14-Cterm_FASTA:248-270	GGGACCTCTGAAGGTGTACTTGG
JT-IVT-44*	PSMB4	PSMB4_Cterm_83forw	TGGACAGTACAGCTATTTTTAGG
JT-IVT-45	PSMB4	PSMB4_Cterm_127rev	TCATTCAAAGCCACTAAAAAAGG
JT-IVT-46	PSMB4	PSMB4_Cterm_166rev	AAGTAGGGCAACTTCAGTTCTGG
JT-IVT-47	PSMB4	PSMB4_Cterm_202forw	CCTACTTTTAACTTTGAACTTGG
JT-IVT-48*	PSMB4	PSMB4_Cterm_181rev	CAAGTTCAAAGTTAAAAGTAGGG
JT-IVT-49	PSMB4	PSMB4_Cterm_182rev	CCAAGTTCAAAGTTAAAAGTAGG

* Guide RNAs used for knock-in experiments.

Table S2. Chromosomal locations of PSMD14 and PSMB4 as per GRCh38/Hg38, related to Figure 1.

Name	Regions	Chromosomal location
hRpn11-01	IVT-2995	chr2:161,411,304-161,411,326
hRpn11-02	IVT-2997	chr2:161,411,471-161,411,493
PSMB4-01	JT-IVT-44	chr1:151,401,743-151,401,765
PSMB4-02	JT-IVT-48	chr1:151,401,861-151,401,883

Table S3. Oligos for guide RNAs, related to Figure 1.

Name	Forward oligo	Reverse oligo
IVT-2995	CACCGGTTCCTCCAAATGACGTTTG	AAACCAAACGTCATTTGGAGGAACC
IVT-2997	ACCGGGGACCTCTGAAGGTGTACT GT	TAAAACAGTACACCTTCAGAGGTCCC
JT-IVT-44	CACCGTGGACAGTACAGCTATTTTT	AAACAAAAATAGCTGTACTGTCCAC
JT-IVT-48	ACCGCAAGTTCAAAGTTAAAAGTA GT	TAAAAC ACTTTTAACTTTGAACTTG

Table S4. Details of the primers used in the study, related to Figure 1.

Primer Name	Primer sequence
PSMD14-GT-V1-F	GTTAGAAAAGCATGGCCAAG
PSMD14-GT-V1-R	CTATTAGAAAACTGGGTAGTC
PSMD14-GT-V2-F	GTTAGGAGTCTTGGGTATTT
PSMD14-GT-V2-R	AGATGAGACCAAACAATAGC
PSMB4-GT-V1-F	CGCTATTACTGGGCAGATGG
PSMB4-GT-V1-R	CTGCCTGCATGGCTTAGTAG
PSMB4-GT-V2-F	GTGGTTGTCTGCTTTTCTCC
PSMB4-GT-V2-R	CTGTGTTGAGACTGAATGTG

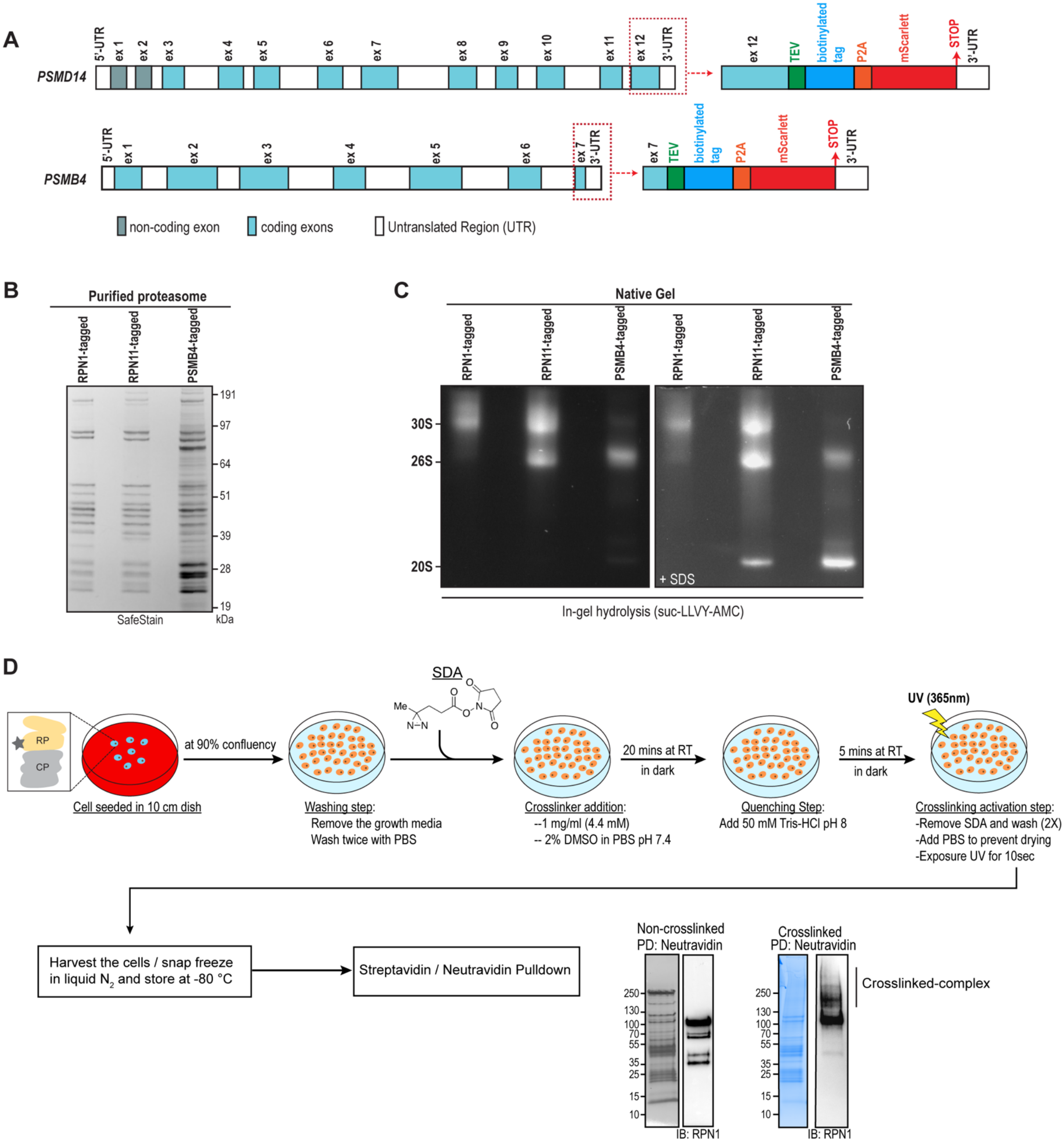

Figure S1. Gene editing and crosslinking methodology for RPN1-, RPN11-, and PSMB4- tagged cell lines, related to Figure 1. **(A)** Schematic representation of the *PSMD14* and *PSMB4* gene with their respective coding and non-coding exons and untranslated regions (UTR). CRISPR editing of *RPN11* and *PSMB4* at their terminal 3'-exon introduced a purification handle which contains a TEV-cleavable biotinylated tag for purification and P2A-

mediated auto cleavable mScarlet signal. **(B)** Purified proteasome complexes (1 μg) from RPN1-, RPN11-, and PSMB4-tagged cell lines were separately loaded onto a denaturing SDS-PAGE gel and stained with safe stain. **(C)** Purified proteasome complexes (2 μg) from RPN1-, RPN11-, and PSMB4-tagged cell lines were subjected to native-PAGE gel followed by in-gel peptidase activity by incubating with gel peptidase buffer (containing suc-LLVY-AMC peptide) without or with SDS. **(D)** Illustration of the in situ photo-crosslinking workflow. In short, tagged cells were grown to 90% confluency, media was removed via PBS washes, and cells were crosslinked with SDA (4.4 mM, 20 mins., room temperature) in the dark. Cells were re-washed with PBS and irradiated with 365 nm wavelength light for 10 seconds (see methods). Cells were then harvested, lysed, and the tagged proteins were enriched. Crosslinking was visualized as band streakiness and upward shifting on a gel via immunoblotting.

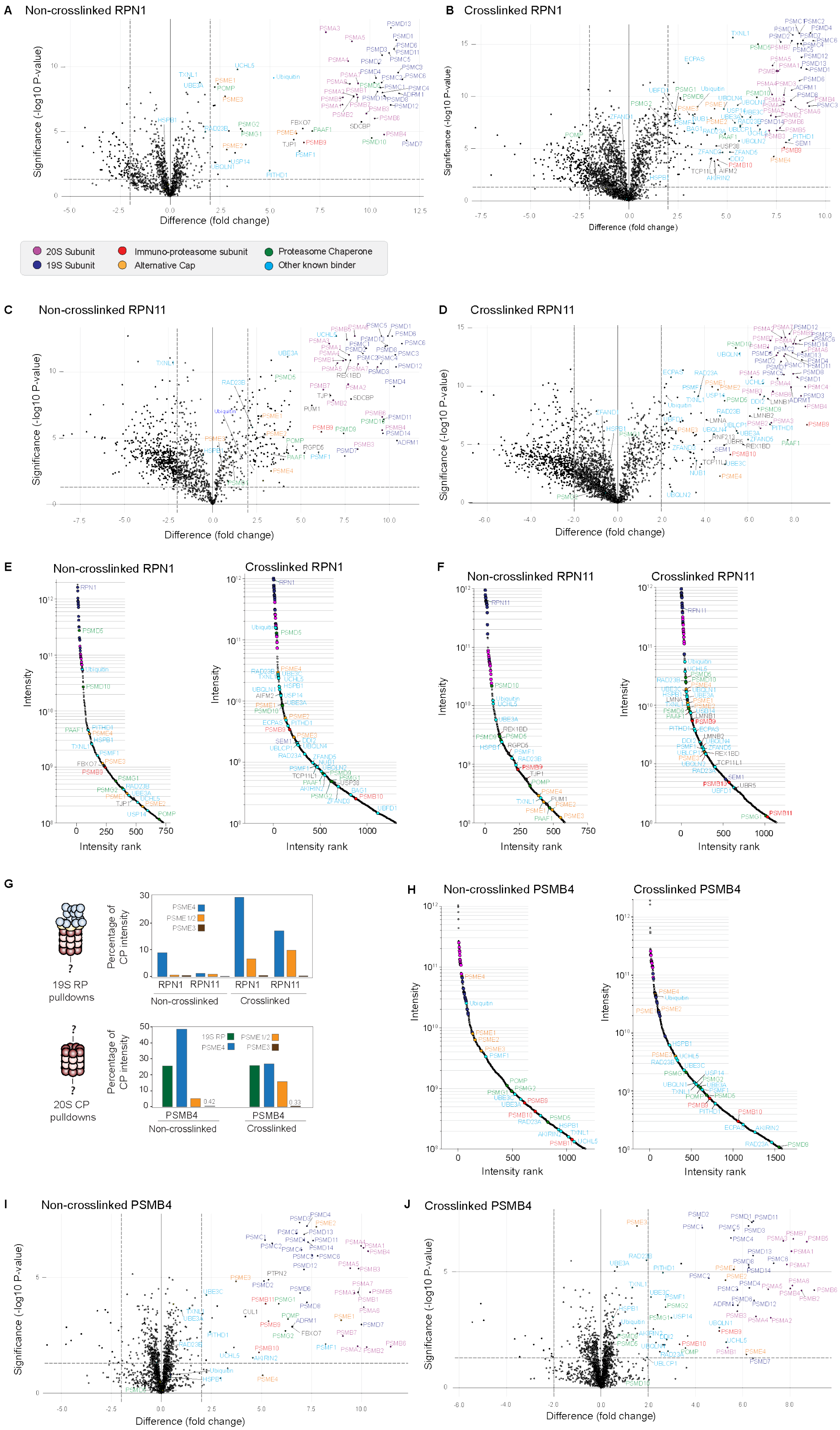

A Non-crosslinked RPN1
B Crosslinked RPN1
Significance (-log10 P-value)
Difference (fold change)
20S Subunit
19S Subunit
Immuno-proteasome subunit
Alternative Cap
Proteasome Chaperone
Other known binder
C Non-crosslinked RPN11
D Crosslinked RPN11
E Non-crosslinked RPN1
Crosslinked RPN1
F Non-crosslinked RPN11
Crosslinked RPN11
Intensity
Intensity rank
G
19S RP pulldowns
20S CP pulldowns
Percentage of CP intensity
PSME4
PSME 1/2
PSME3
RPN1
RPN11
Non-crosslinked
Crosslinked
19S RP
PSME 1/2
PSME4
PSME3
PSMB4
0.42
0.33
H Non-crosslinked PSMB4
Crosslinked PSMB4
I Non-crosslinked PSMB4
J Crosslinked PSMB4

Figure S2. AP-MS enrichment plots from biotin-tagged proteasome subunit pulldowns, related to Figure 1. (A-D, I-J) Volcano plots depicting enrichment of pulled-down proteins for non-crosslinked (**A, C, I**) or crosslinked (**B, D, J**) for RPN1 (**A, B**), RPN11 (**C, D**), and PSMB4 (**I, J**) affinity-purification mass spectrometry (AP-MS) experiments. Enrichment is measured against pulldowns from parental HCT116 cells, either non-crosslinked (**A, C, I**) or crosslinked (**B, D, J**). Proteins are color-coded depending on whether they are part of the CP (pink), RP (dark blue), an immuno-proteasome subunit (red), an alternative cap (orange), a proteasome chaperone (green), or another known proteasome binder (cyan). The dashed line on the y-axis indicates the level of significance (P-value with BH correction < 0.05) and the dashed lines on the x-axis indicate the difference in abundance (log_2-fold change) between tagged and wild-type pulldown samples. Each dataset contains three independent biological replicates injected twice on the mass spectrometer. FragPipe/MSFragger was used to generate label-free quantification (LFQ) for the identified proteins, and that output was subsequently analyzed using QProMS to generate volcano plots, which were redrawn using custom Python scripts. **(E, F, H)** Ranked intensity plots for protein abundances using LFQ for non-crosslinked (left) and crosslinked (right) RPN1 **(E)**, RPN11 **(F)**, and PSMB4 **(H)** AP-MS experiments. **(G)** Bar plots for the non-crosslinked and crosslinked AP-MS datasets that compare the abundances of 19S RP (green), PSME4 (blue), PSME1/2 (orange), and PSME3 (brown) as the percentage of 20S CP intensity for 19S RP (top) or 20S CP pulldowns (bottom).

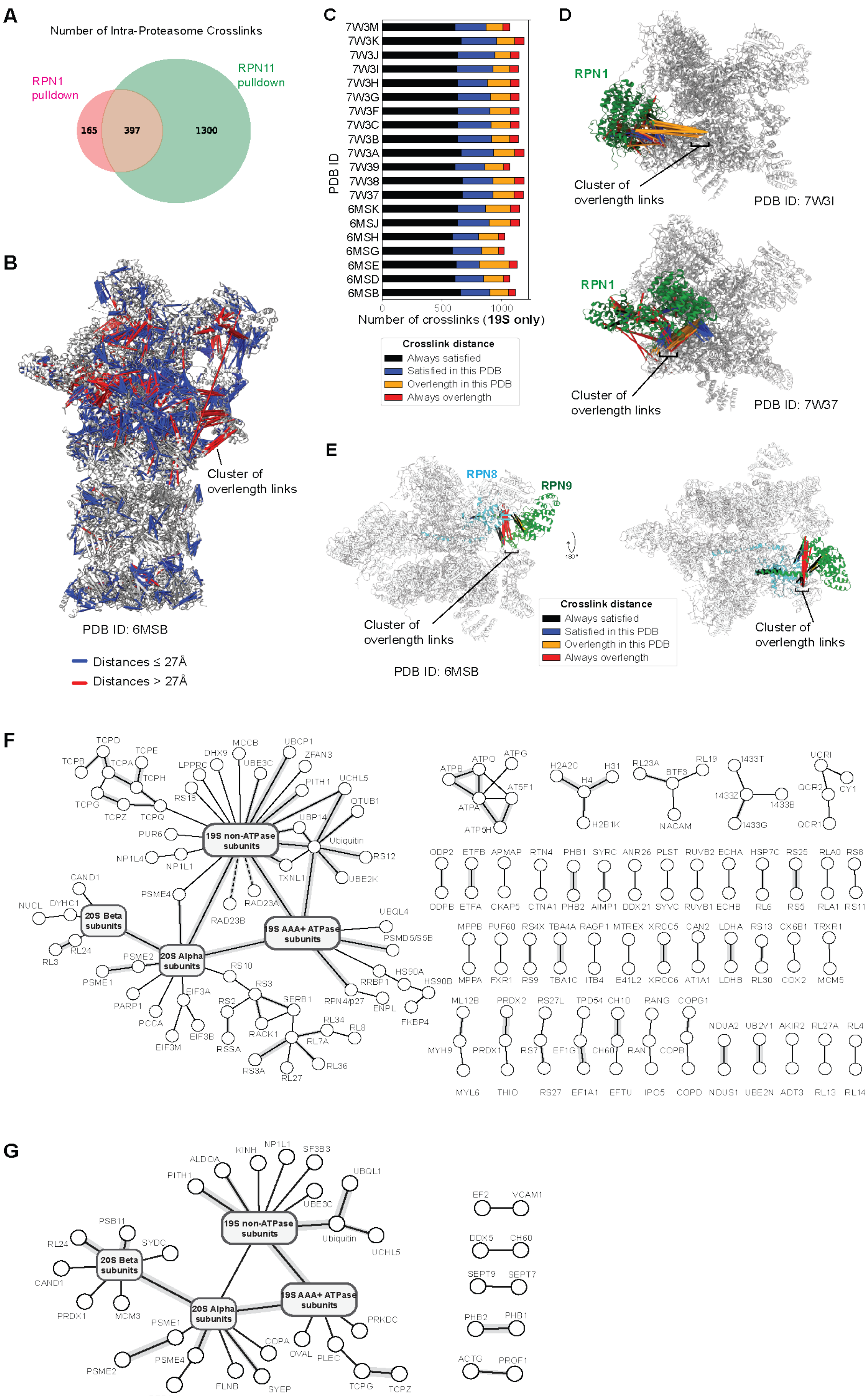

A
Number of Intra-Proteasome Crosslinks
RPN1 pulldown
RPN11 pulldown
165
397
1300
B
Cluster of overlength links
PDB ID: 6MSB
Distances ≤ 27Å
Distances > 27Å
C
7W3M
7W3K
7W3J
7W3I
7W3H
7W3G
7W3F
7W3C
7W3B
7W3A
7W39
7W38
7W37
6MSK
6MSJ
6MSH
6MSG
6MSE
6MSD
6MSB
PDB ID
0
500
1000
Number of crosslinks (19S only)
Crosslink distance
Always satisfied
Satisfied in this PDB
Overlength in this PDB
Always overlength
D
RPN1
Cluster of overlength links
PDB ID: 7W3I
RPN1
Cluster of overlength links
PDB ID: 7W37
E
RPN8
RPN9
Cluster of overlength links
PDB ID: 6MSB
180°
Crosslink distance
Always satisfied
Satisfied in this PDB
Overlength in this PDB
Always overlength
Cluster of overlength links
F
19S non-ATPase subunits
20S Beta subunits
20S Alpha subunits
19S AAA+ ATPase subunits
Ubiquitin
TCPD
TCPE
TCPB
TCPA
TCPH
TCPG
TCPZ
TCPQ
DHX9
MCCB
UBCP1
LPPRC
UBE3C
ZFAN3
PITH1
UCHL5
RS18
OTUB1
UBP14
PUR6
NP1L4
NP1L1
RS12
TXNL1
UBE2K
CAND1
PSME4
RAD23A
RAD23B
NUCL
DYHC1
UBQL4
PSMD5/S5B
RL24
RL3
PSME2
PSME1
RS10
HS90A
HS90B
RRBP1
ENPL
RPN4/p27
FKBP4
EIF3A
RS3
PARP1
SERB1
RS2
PCCA
EIF3B
EIF3M
RACK1
RSSA
RL34
RL8
RL7A
RS3A
RL36
RL27
ATPB
ATPO
ATPG
ATP5F1
ATPA
ATP5H
H2A2C
H31
H4
H2B1K
RL23A
BTF3
RL19
NACAM
1433T
1433Z
1433B
1433G
UCRI
QCR2
CY1
QCR1
ODP2
ODPB
ETFB
ETFA
APMAP
CKAP5
RTN4
CTNA1
PHB1
PHB2
SYRC
AIMP1
ANR26
DDX21
PLST
SYVC
RUVB2
RUVB1
ECHA
ECHB
HSP7C
RL6
RS25
RS5
RLA0
RLA1
RS8
RS11
MPPB
MPPA
PUF60
FXR1
RS4X
RS9
TBA4A
TBA1C
RAGP1
ITB4
MTREX
E41L2
XRCC5
XRCC6
CAN2
AT1A1
LDHA
LDHB
RS13
RL30
CX6B1
COX2
TRXR1
MCM5
ML12B
MYH9
MYL6
PRDX2
PRDX1
THIO
RS27L
RS7
RS27
TPD54
EF1G
EF1A1
CH10
CH60
EFTU
RANG
RAN
IPO5
COPG1
COPB
COPD
NDUA2
NDUS1
UB2V1
UBE2N
AKIR2
ADT3
RL27A
RL13
RL4
RL14
G
19S non-ATPase subunits
20S Beta subunits
20S Alpha subunits
19S AAA+ ATPase subunits
Ubiquitin
KINH
NP1L1
SF3B3
ALDOA
PITH1
UBQL1
UBE3C
UCHL5
PSB11
RL24
SYDC
CAND1
PRDX1
MCM3
PSME1
PSME4
PSME2
PRP6
FLNB
SYEP
COPA
OVAL
PLEC
PRKDC
TCPG
TCPZ
EF2
VCAM1
DDX5
CH60
SEPT9
SEPT7
PHB2
PHB1
ACTG
PROF1

Figure S3. Crosslinks on proteasome structures, related to Figure 2. **(A)** Overlap of crosslinked residue pairs between subunits of the proteasome identified in the RPN1 and RPN11 pulldowns. Data come from 90 or 152 MS acquisitions from crosslinked RPN1 or RPN11 pulldown material, respectively. **(B)** A proteasome structure (PDB ID: 6MSB) with crosslinks mapped as pseudobonds and colored depending on Euclidean distance between residue-pair Cα–Cα distances. Crosslink-based distances of $\leq$ 27 Å are considered 'satisfied' (blue). Crosslinks that exceed this distance are colored in red. **(C)** Bar plot illustrating 20 different proteasome models (y-axis, PDB IDs) and whether the mapped crosslinks (x-axis, number of crosslinks mapped to the 19S subunit) satisfied the distance restraint. 93.5 % of crosslinks were considered satisfied in at least one state. **(D)** Clusters of overlength crosslinks from photo-crosslinking MS indicate conformational changes. RPN1 is particularly flexible in relation to the rest of the RP; in PDB IDs 7W3I and 7W37, different clusters of crosslink distance-restraints are satisfied. **(E)** A cluster of overlength crosslinks between RPN8 and RPN9 remains unsatisfied in all investigated proteasome structures. This suggests that an unsolved configuration exists between these subunits. **(F)** 276 distinct PPIs from RPN1 and RPN11 datasets combined at an estimated combined 7.7 % PPI-level FDR. Subunits of the 20S CP and the 19S RP are merged for simplicity. Each edge represents one or more crosslinks and line thickness scales with number of unique residue pairs per protein-protein interaction. The crosslink mapping to RAD23A or RAD23B is to a common peptide sequence prohibiting each to be distinguished. **(G)** 156 distinct protein-protein interactions discovered by crosslinking MS at 5 % FDR from the PSMB4 pulldown.

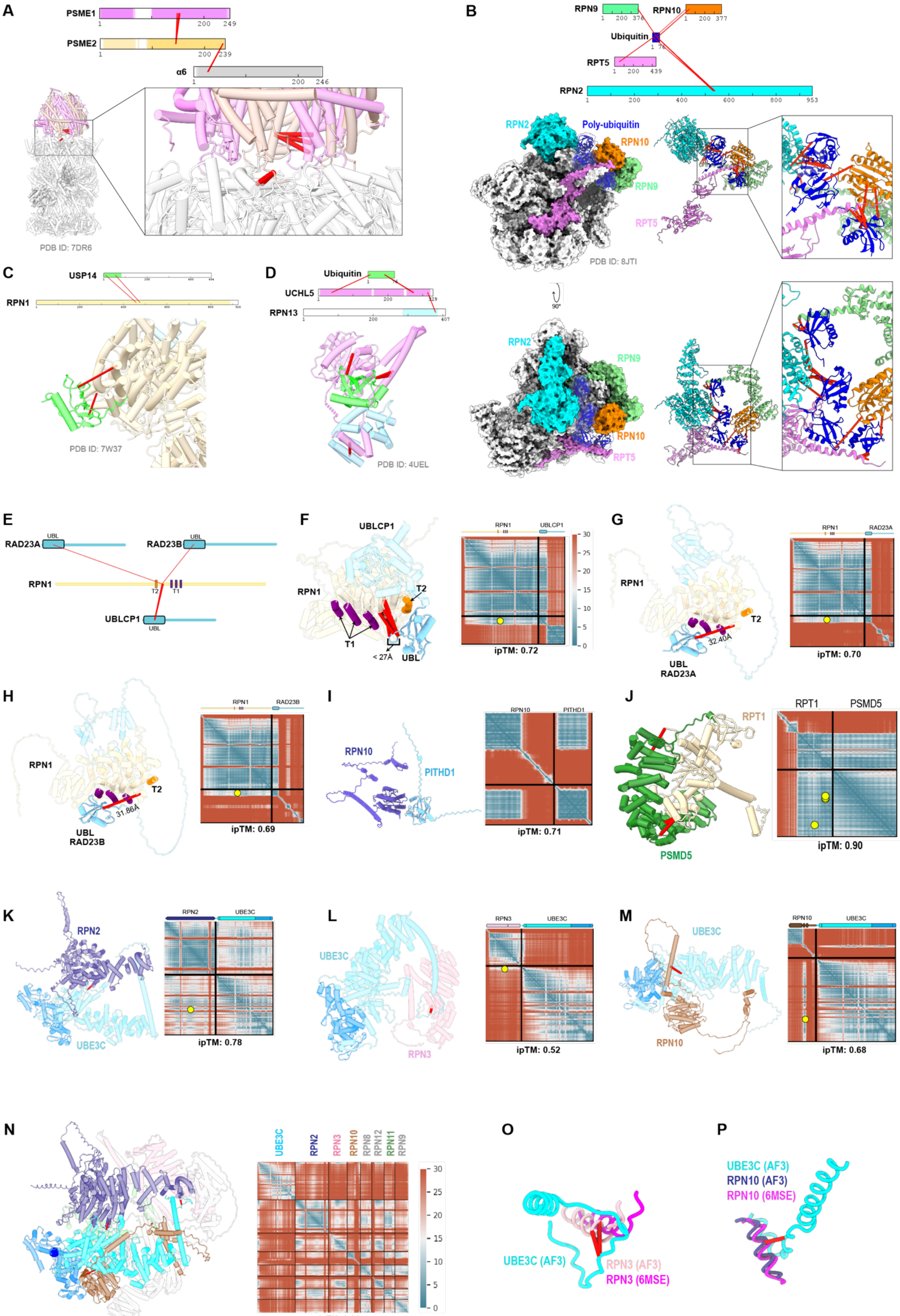

A
PSME1
PSME2
α6
PDB ID: 7DR6
B
RPN9
RPN10
Ubiquitin
RPT5
RPN2
RPN2
Poly-ubiquitin
RPN10
RPN9
RPT5
PDB ID: 8JTI
90°
RPN2
RPN9
RPN10
RPT5
C
USP14
RPN1
PDB ID: 7W37
D
Ubiquitin
UCHL5
RPN13
PDB ID: 4UEL
E
RAD23A
RAD23B
UBL
RPN1
T2
T1
UBLCP1
F
UBLCP1
RPN1
T1
T2
UBL
< 27Å
ipTM: 0.72
G
RPN1
T2
32.40Å
UBL
RAD23A
ipTM: 0.70
H
RPN1
T2
31.86Å
UBL
RAD23B
ipTM: 0.69
I
RPN10
PITHD1
ipTM: 0.71
J
RPT1
PSMD5
ipTM: 0.90
K
RPN2
UBE3C
ipTM: 0.78
L
UBE3C
RPN3
ipTM: 0.52
M
UBE3C
RPN10
ipTM: 0.68
N
UBE3C
RPN2
RPN3
RPN10
RPN8
RPN12
RPN11
RPN9
ipTM: 0.64
O
UBE3C (AF3)
RPN3 (AF3)
RPN3 (6MSE)
P
UBE3C (AF3)
RPN10 (AF3)
RPN10 (6MSE)

Figure S4. Crosslinks on known and predicted structures for transiently associated proteins with proteasomes or their intrinsic subunits, related to Figure 2. **(A)** Crosslinks on structure of the CP bound to the PSME1/2 alternate cap (PDB ID: 7DR6). **(B)** In situ-derived crosslinks on the structure of a K11/K48-branched ubiquitin chain on the 19S RP. Identified crosslinks with RPN2, RPN9, RPN10, and RPT5 agree with the available structure (PDB ID: 8JTI). **(C)** Crosslinks on the structure of USP14 bound to RPN1 (PDB ID: 7W37). **(D)** Crosslinks on the structure of the RPN13, UCHL5, and ubiquitin complex (PDB ID: 4UEL). **(E)** Bar representation of RPN1 and its crosslinked binders UBLCP1 and RAD23A/B with the crosslinked connections in red. The UBL domains and T1 and T2 sites are displayed and labeled. **(F-L)** AlphaFold-predicted as binary complexes (left) with ipTM scores >0.65, including **(F)** the UBLCP1 UBL domain bound to the RPN1 T2 site, RPN1 T1 bound to **(G)** RAD23A and **(H)** RAD23B UBL domains, **(I)** RPN10 VWA bound to PITHD1, **(J)** PSMD5 with RPT1, UBE3C bound to **(K)** RPN2, **(L)** RPN3, or **(M)** RPN10 VWA. No crosslinks were detected between RPN10 and PITHD1. In (K) – (N), UBE3C is shown in cyan with the HECT domain dark blue. (**N)** AlphaFold3 model comprising RPN2, RPN3, RPN8, RPN9, RPN10, RPN11, RPN12, and UBE3C that was used in Figure 2E. Crosslinked sites are depicted in red. Predicted alignment error (PAE) plots are included (right) showing residue-by-residue confidence scores of the predictions based on pairwise residue alignments with a yellow dot representing crosslinked residues. **(O-P)** Expanded regions of UBE3C (cyan) bound to RPN3 (aa 288-315, O) or RPN10 (aa 525-538, P) in the AlphaFold3 model of panel N (light pink in O and blue in P) superimposed as in Figure 2E onto a human 26S proteasome cryo-EM structure (PDB 6MSE, magenta in O and P). UBE3C residues 1 – 38 are displayed in (O) and 539 – 579 in (P).

A

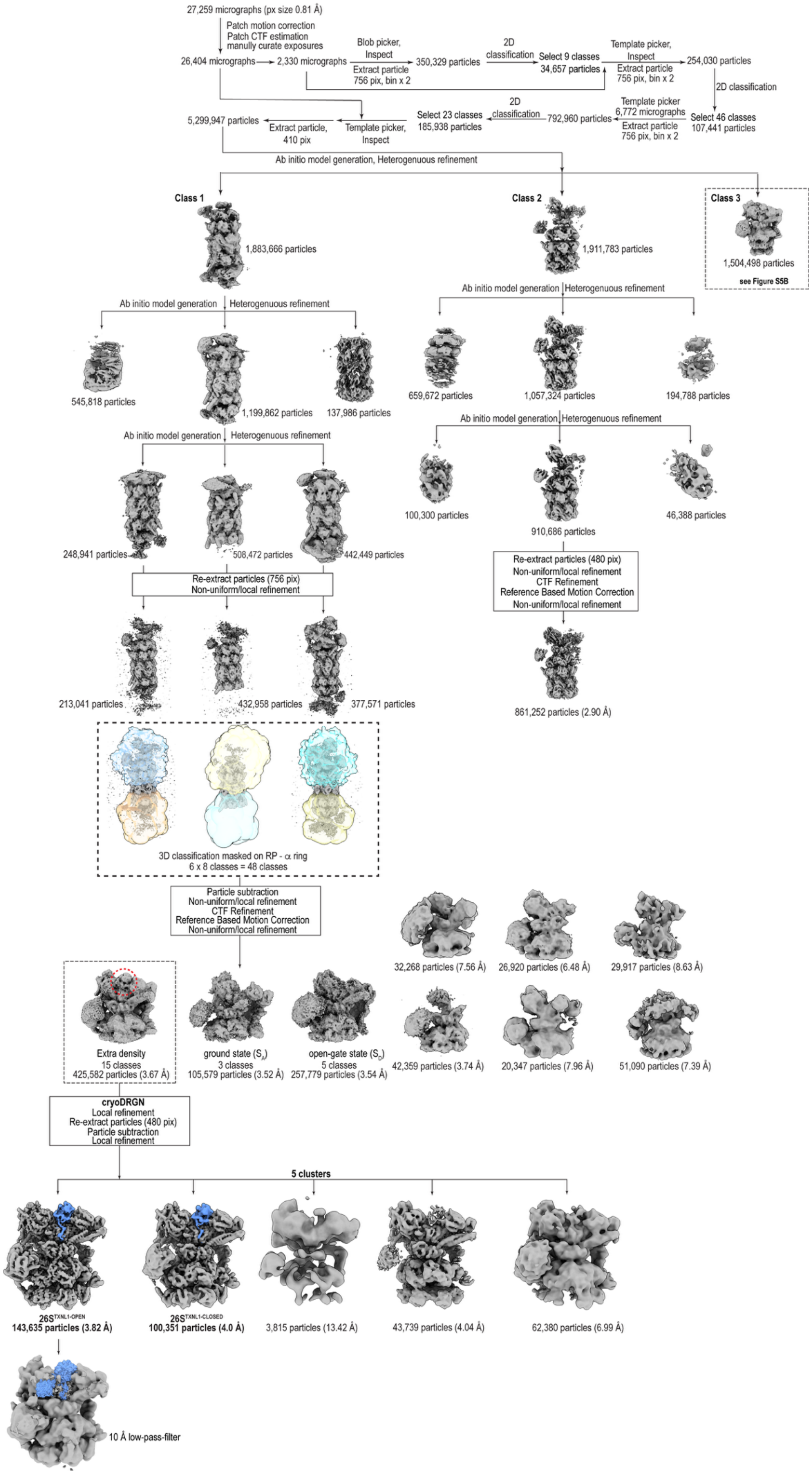

27,259 micrographs (px size 0.81 Å)
Patch motion correction
Patch CTF estimation
manully curate exposures
26,404 micrographs
2,330 micrographs
Blob picker,
Inspect
Extract particle
756 pix, bin x 2
350,329 particles
2D
classification
Select 9 classes
34,657 particles
Template picker,
Inspect
Extract particle
756 pix, bin x 2
254,030 particles
2D classification
Select 46 classes
107,441 particles
Template picker
6,772 micrographs
Extract particle
756 pix, bin x 2
792,960 particles
2D
classification
Select 23 classes
185,938 particles
Template picker,
Inspect
Extract particle,
410 pix
5,299,947 particles
Ab initio model generation, Heterogenuous refinement
Class 1
1,883,666 particles
Class 2
1,911,783 particles
Class 3
1,504,498 particles
see Figure S5B
Ab initio model generation
Heterogenuous refinement
545,818 particles
1,199,862 particles
137,986 particles
Ab initio model generation
Heterogenuous refinement
659,672 particles
1,057,324 particles
194,788 particles
Ab initio model generation
Heterogenuous refinement
Ab initio model generation
Heterogenuous refinement
248,941 particles
508,472 particles
442,449 particles
100,300 particles
910,686 particles
46,388 particles
Re-extract particles (756 pix)
Non-uniform/local refinement
Re-extract particles (480 pix)
Non-uniform/local refinement
CTF Refinement
Reference Based Motion Correction
Non-uniform/local refinement
213,041 particles
432,958 particles
377,571 particles
861,252 particles (2.90 Å)
3D classification masked on RP - α ring
6 x 8 classes = 48 classes
Particle subtraction
Non-uniform/local refinement
CTF Refinement
Reference Based Motion Correction
Non-uniform/local refinement
32,268 particles (7.56 Å)
26,920 particles (6.48 Å)
29,917 particles (8.63 Å)
Extra density
15 classes
425,582 particles (3.67 Å)
ground state (S_A)
3 classes
105,579 particles (3.52 Å)
open-gate state (S_D)
5 classes
257,779 particles (3.54 Å)
42,359 particles (3.74 Å)
20,347 particles (7.96 Å)
51,090 particles (7.39 Å)
cryoDRGN
Local refinement
Re-extract particles (480 pix)
Particle subtraction
Local refinement
5 clusters
$26S^{TXNL1\text{-}OPEN}$
143,635 particles (3.82 Å)
$26S^{TXNL1\text{-}CLOSED}$
100,351 particles (4.0 Å)
3,815 particles (13.42 Å)
43,739 particles (4.04 Å)
62,380 particles (6.99 Å)
10 Å low-pass-filter

B

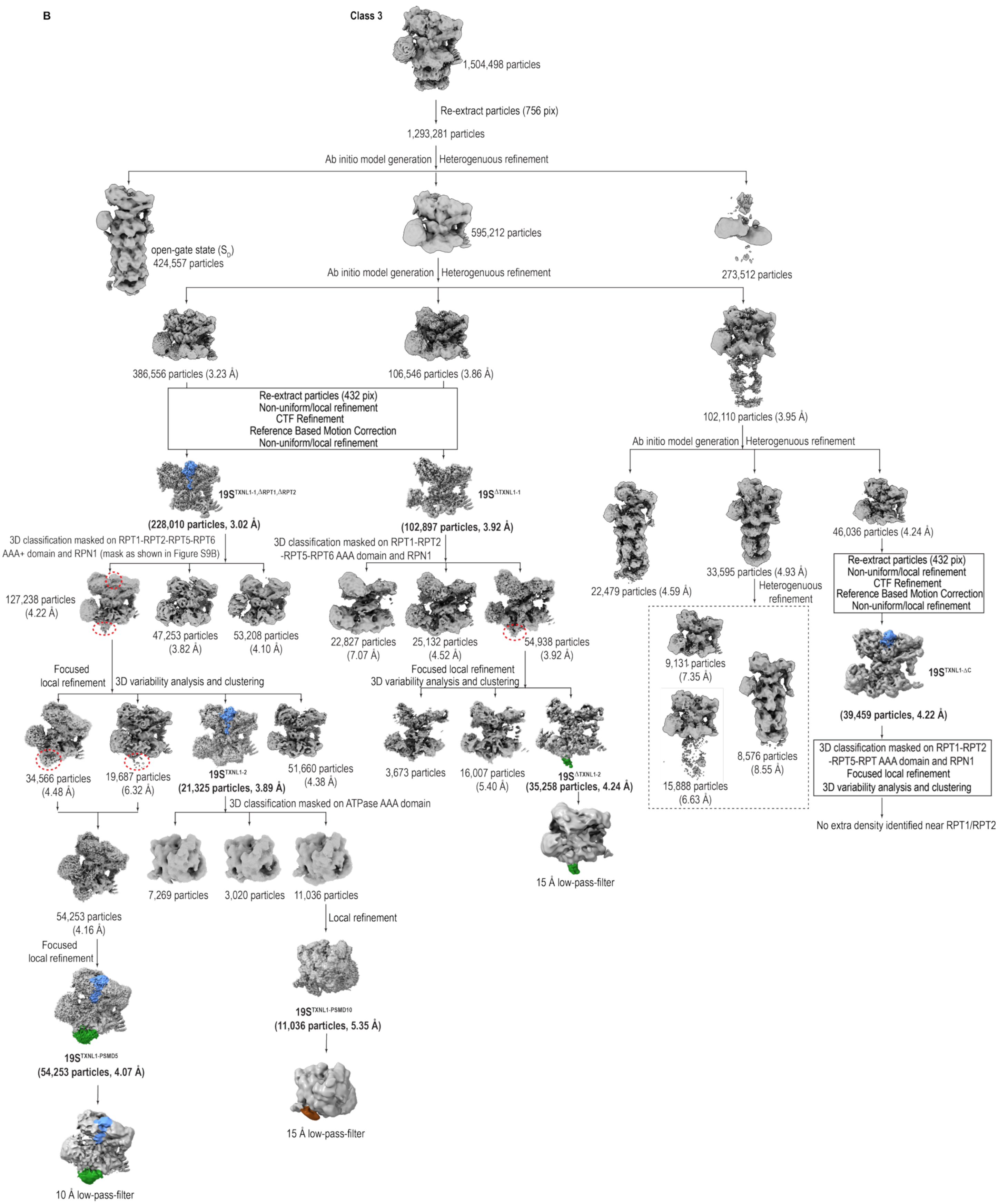
Class 3
1,504,498 particles
Re-extract particles (756 pix)
1,293,281 particles
Ab initio model generation
Heterogenuous refinement
open-gate state (S_D)
424,557 particles
595,212 particles
273,512 particles
Ab initio model generation
Heterogenuous refinement
386,556 particles (3.23 Å)
106,546 particles (3.86 Å)
102,110 particles (3.95 Å)
Re-extract particles (432 pix)
Non-uniform/local refinement
CTF Refinement
Reference Based Motion Correction
Non-uniform/local refinement
$19S^{TXNL1-1,\Delta RPT1,\Delta RPT2}$
(228,010 particles, 3.02 Å)
$19S^{\Delta TXNL1-1}$
(102,897 particles, 3.92 Å)
Ab initio model generation
Heterogenuous refinement
22,479 particles (4.59 Å)
33,595 particles (4.93 Å)
46,036 particles (4.24 Å)
3D classification masked on RPT1-RPT2-RPT5-RPT6
AAA+ domain and RPN1 (mask as shown in Figure S9B)
3D classification masked on RPT1-RPT2
-RPT5-RPT6 AAA domain and RPN1
127,238 particles
(4.22 Å)
47,253 particles
(3.82 Å)
53,208 particles
(4.10 Å)
22,827 particles
(7.07 Å)
25,132 particles
(4.52 Å)
54,938 particles
(3.92 Å)
Heterogenuous
refinement
9,131 particles
(7.35 Å)
15,888 particles
(6.63 Å)
8,576 particles
(8.55 Å)
Re-extract particles (432 pix)
Non-uniform/local refinement
CTF Refinement
Reference Based Motion Correction
Non-uniform/local refinement
$19S^{TXNL1-\Delta C}$
(39,459 particles, 4.22 Å)
3D classification masked on RPT1-RPT2
-RPT5-RPT AAA domain and RPN1
Focused local refinement
3D variability analysis and clustering
No extra density identified near RPT1/RPT2
Focused
local refinement
3D variability analysis and clustering
Focused local refinement
3D variability analysis and clustering
34,566 particles
(4.48 Å)
19,687 particles
(6.32 Å)
$19S^{TXNL1-2}$
(21,325 particles, 3.89 Å)
51,660 particles
(4.38 Å)
3,673 particles
16,007 particles
(5.40 Å)
$19S^{\Delta TXNL1-2}$
(35,258 particles, 4.24 Å)
15 Å low-pass-filter
3D classification masked on ATPase AAA domain
7,269 particles
3,020 particles
11,036 particles
54,253 particles
(4.16 Å)
Focused
local refinement
Local refinement
$19S^{TXNL1-PSMD10}$
(11,036 particles, 5.35 Å)
15 Å low-pass-filter
$19S^{TXNL1-PSMD5}$
(54,253 particles, 4.07 Å)
10 Å low-pass-filter

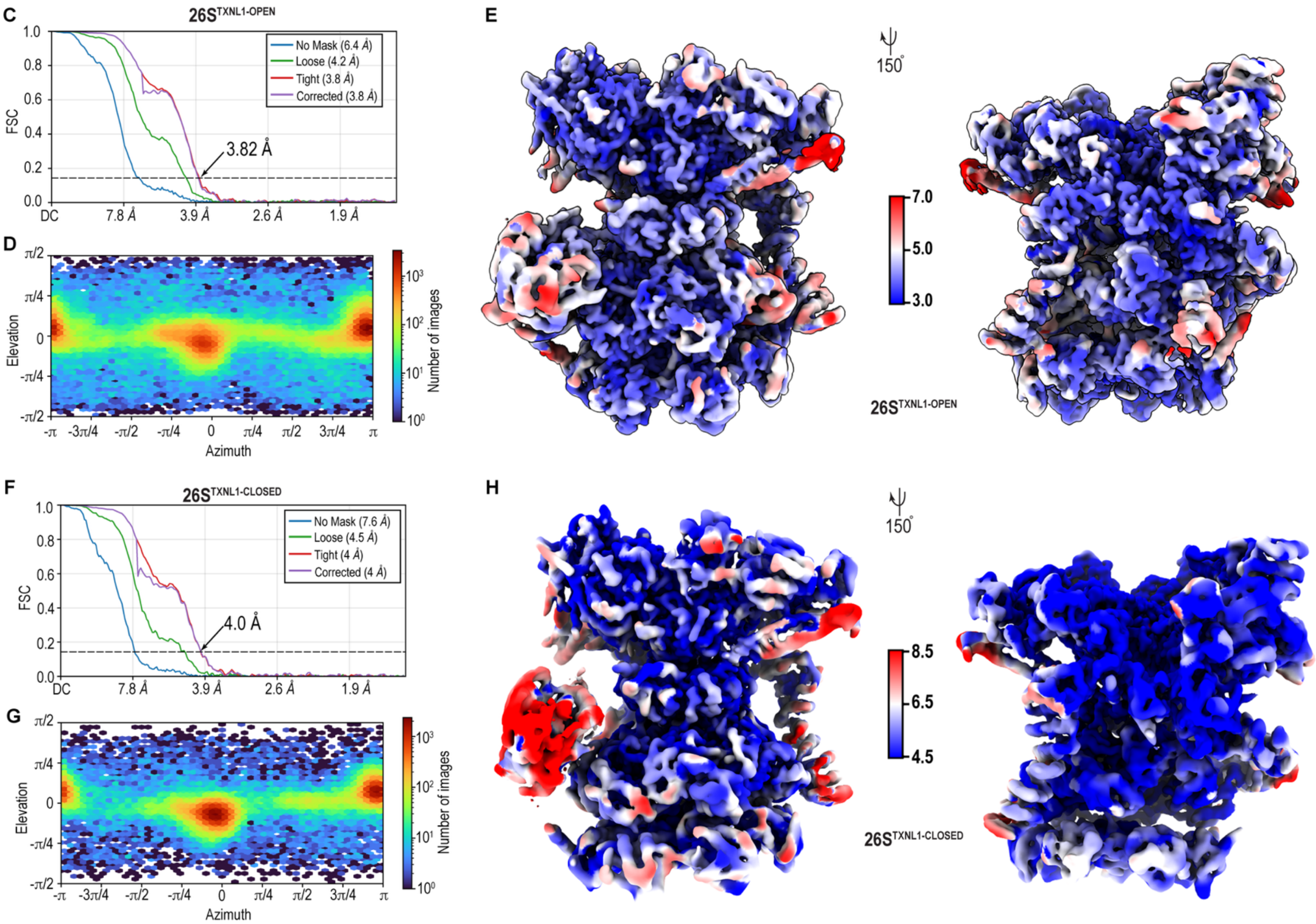
C
26S TXNL1-OPEN
FSC
No Mask (6.4 Å)
Loose (4.2 Å)
Tight (3.8 Å)
Corrected (3.8 Å)
3.82 Å
DC
7.8 Å
3.9 Å
2.6 Å
1.9 Å
D
Elevation
Azimuth
Number of images
E
150°
7.0
5.0
3.0
26S TXNL1-OPEN
F
26S TXNL1-CLOSED
No Mask (7.6 Å)
Loose (4.5 Å)
Tight (4 Å)
Corrected (4 Å)
4.0 Å
G
H
8.5
6.5
4.5
26S TXNL1-CLOSED

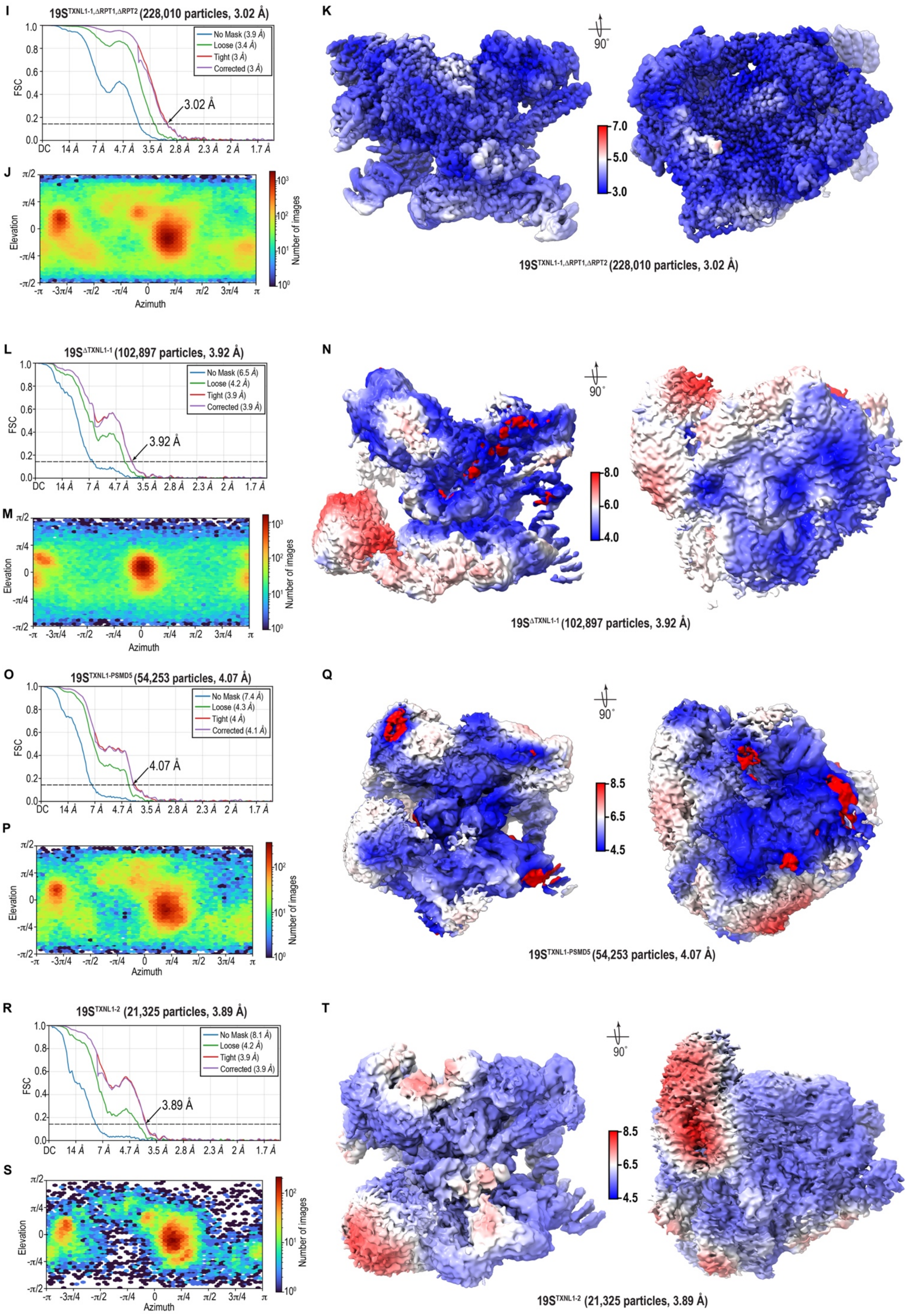

I
19S TXNL1-1,ΔRPT1,ΔRPT2 (228,010 particles, 3.02 Å)
No Mask (3.9 Å)
Loose (3.4 Å)
Tight (3 Å)
Corrected (3 Å)
3.02 Å
FSC
DC 14 Å 7 Å 4.7 Å 3.5 Å 2.8 Å 2.3 Å 2 Å 1.7 Å
J
Elevation
Azimuth
Number of images
K
90°
7.0
5.0
3.0
19S TXNL1-1,ΔRPT1,ΔRPT2 (228,010 particles, 3.02 Å)
L
19S ΔTXNL1-1 (102,897 particles, 3.92 Å)
No Mask (6.5 Å)
Loose (4.2 Å)
Tight (3.9 Å)
Corrected (3.9 Å)
3.92 Å
M
N
8.0
6.0
4.0
19S ΔTXNL1-1 (102,897 particles, 3.92 Å)
O
19S TXNL1-PSMD5 (54,253 particles, 4.07 Å)
No Mask (7.4 Å)
Loose (4.3 Å)
Tight (4 Å)
Corrected (4.1 Å)
4.07 Å
P
Q
8.5
6.5
4.5
19S TXNL1-PSMD5 (54,253 particles, 4.07 Å)
R
19S TXNL1-2 (21,325 particles, 3.89 Å)
No Mask (8.1 Å)
Loose (4.2 Å)
Tight (3.9 Å)
Corrected (3.9 Å)
3.89 Å
S
T
8.5
6.5
4.5
19S TXNL1-2 (21,325 particles, 3.89 Å)

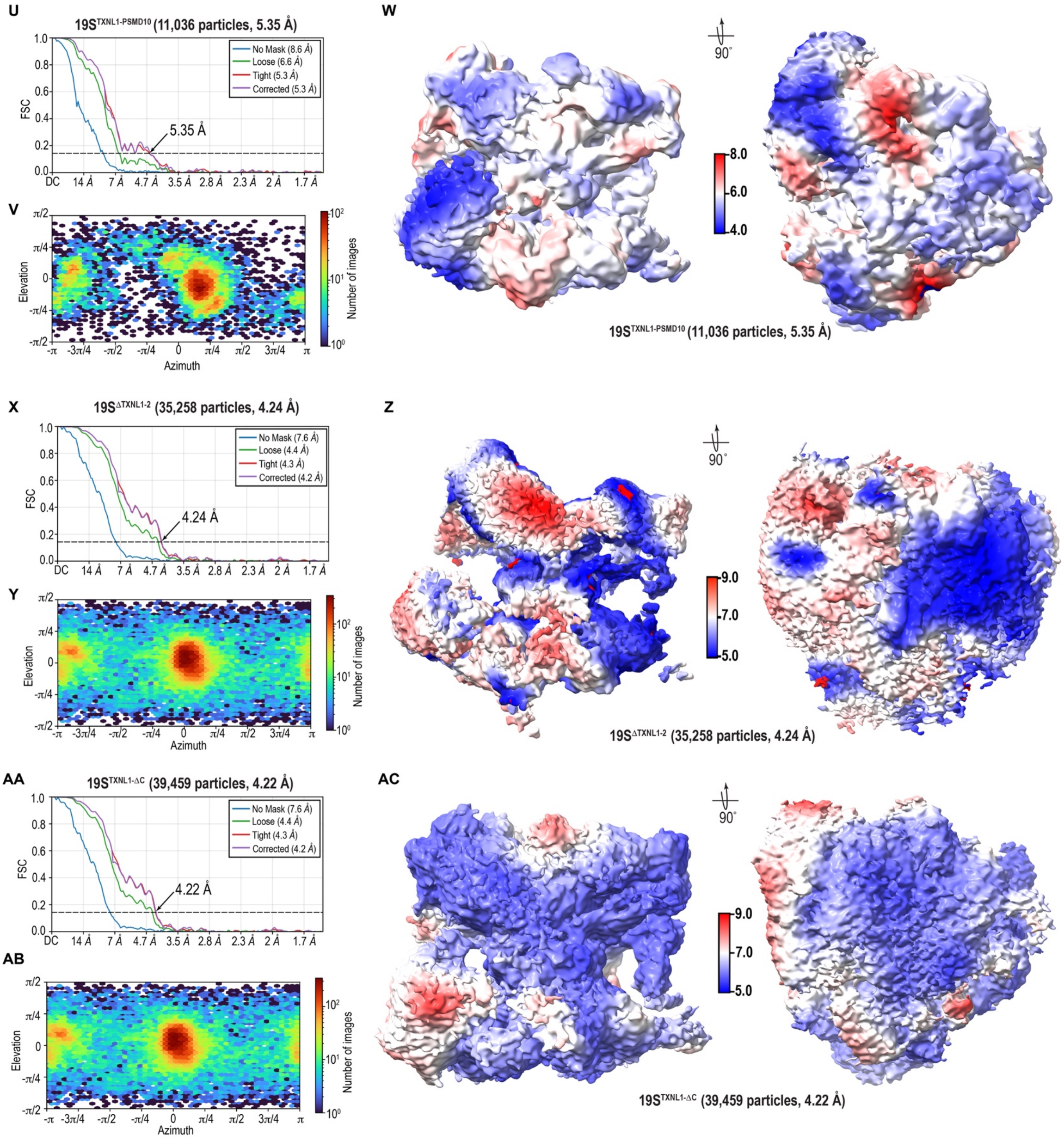

Figure S5. Cryo-EM workflow for proteasome complexes purified using an RPN1-affinity tag, related to Figure 3, 4, and 6 (A) Processing scheme for the cryo-EM classification process for the full dataset showing reconstructions following refinement of $26S^{TXNL1\text{-}OPEN}$ and $26S^{TXNL1\text{-}CLOSED}$. Data were collected on proteasomes isolated from RPN1-tagged HCT116 cells by using a Talos Arctica G2 electron microscope (Thermo Fisher

Scientific, Inc) equipped with a K3 direct electron detector (Gatan) and an energy filter, operating at 200 kV in super resolution mode (pixel size 0.405 Å/pixel, x 100,000 nominal magnification). **(B)** Processing scheme for the classification of class 3 particles from panel A showing reconstructions following refinement of $19S^{TXNL1\text{-}1,\Delta RPT1,\Delta RPT2}$, $19S^{\Delta TXNL1\text{-}1}$, $19S^{TXNL1\text{-}PSMD5}$, $19S^{TXNL1\text{-}2}$, $19S^{TXNL1\text{-}PSMD10}$, $19S^{\Delta TXNL1\text{-}2}$, and $19S^{TXNL1\text{-}\Delta C}$. **(C-AC)** GSFSC plots (C, F, I, L, O, R, U, X, and AA), viewing direction distribution plots (D, G, J, M, P, S, V, Y, and AB), and cryo-EM density maps displaying local resolution as calculated by cryoSPARC or Phenix suite (E, H, K, N, Q, T, W, Z, and AC) for the refined maps of $26S^{TXNL1\text{-}OPEN}$ (C, D, and E), $26S^{TXNL1\text{-}CLOSED}$ (F, G, and H), $19S^{TXNL1\text{-}1,\Delta RPT1,\ \Delta RPT2}$ (I, J, and K), $19S^{\Delta TXNL1\text{-}1}$ (L, M, and N), $19S^{TXNL1\text{-}PSMD5}$ (O, P, and Q), $19S^{TXNL1\text{-}2}$ (R, S, and T), $19S^{TXNL1\text{-}PSMD10}$ (U, V, and W), $19S^{\Delta TXNL1\text{-}2}$ (X, Y, and Z), and $19S^{TXNL1\text{-}\Delta C}$ (AA, AB, and AC). FSC value of 0.143 is indicated by a black dashed line in C, F, I, L, O, R, U, X, and AA.

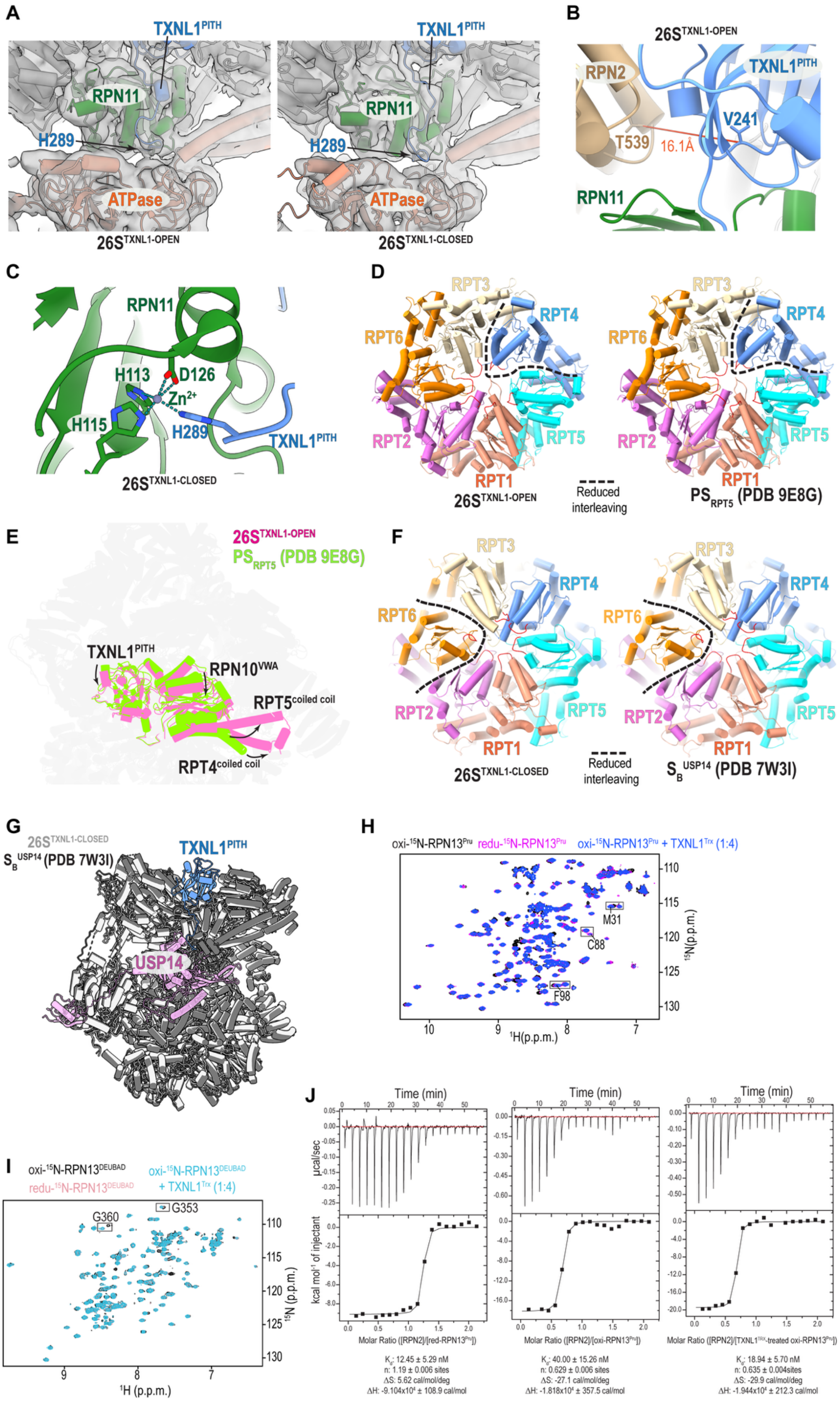
A
TXNL1PITH
RPN11
H289
ATPase
26STXNL1-OPEN
26STXNL1-CLOSED
B
26STXNL1-OPEN
RPN2
TXNL1PITH
V241
T539
16.1Å
RPN11
C
RPN11
H113
D126
Zn2+
H115
H289
TXNL1PITH
26STXNL1-CLOSED
D
RPT3
RPT4
RPT6
RPT5
RPT2
RPT1
26STXNL1-OPEN
Reduced interleaving
PSRPT5 (PDB 9E8G)
E
26STXNL1-OPEN
PSRPT5 (PDB 9E8G)
TXNL1PITH
RPN10VWA
RPT5coiled coil
RPT4coiled coil
F
26STXNL1-CLOSED
Reduced interleaving
SBUSP14 (PDB 7W3I)
G
26STXNL1-CLOSED
SBUSP14 (PDB 7W3I)
TXNL1PITH
USP14
H
oxi-15N-RPN13Pru redu-15N-RPN13Pru oxi-15N-RPN13Pru + TXNL1Trx (1:4)
M31
C88
F98
1H(p.p.m.)
15N(p.p.m.)
I
oxi-15N-RPN13DEUBAD
redu-15N-RPN13DEUBAD
oxi-15N-RPN13DEUBAD + TXNL1Trx (1:4)
G360
G353
1H (p.p.m.)
15N (p.p.m.)
J
Time (min)
µcal/sec
kcal mol-1 of injectant
Molar Ratio ([RPN2]/[red-RPN13Pru])
Molar Ratio ([RPN2]/[oxi-RPN13Pru])
Molar Ratio ([RPN2]/[TXNL1Trx-treated oxi-RPN13Pru])
Kd: 12.45 ± 5.29 nM
n: 1.19 ± 0.006 sites
ΔS: 5.62 cal/mol/deg
ΔH: -9.104x104 ± 108.9 cal/mol
Kd: 40.00 ± 15.26 nM
n: 0.629 ± 0.006 sites
ΔS: -27.1 cal/mol/deg
ΔH: -1.818x104 ± 357.5 cal/mol
Kd: 18.94 ± 5.70 nM
n: 0.635 ± 0.004sites
ΔS: -29.9 cal/mol/deg
ΔH: -1.944x104 ± 212.3 cal/mol

Figure S6. TXNL1 activities at the proteasome, related to Figure 3 and 4. **(A)** Density maps (grey) for $26S^{TXNL1\text{-}OPEN}$ (left) and $26S^{TXNL1\text{-}CLOSED}$ (right) focused on the C-terminus with fitted cartoon models. The coloring scheme follows Figure 3B. **(B)** Expanded view of $26S^{TXNL1\text{-}OPEN}$ showing a crosslink between TXNL1 V241 (blue) and RPN2 T539 (beige), represented as an orange line with the Cα - Cα distance indicated. **(C)** Expanded view of $26S^{TXNL1\text{-}CLOSED}$ to display TXNL1 PITH domain and C-terminal residue H289 coordination with the RPN11 Zn^{2+} (purple sphere) and its additional coordination with H113, H115 and D126 of RPN11. **(D and F)** Top views of the ATPase large AAA+ subdomains in aligned $26S^{TXNL1\text{-}OPEN}$ (D, left) and PS_{RPT5} (PDB 9E8G, D, right), or $26S^{TXNL1\text{-}CLOSED}$ (F, left) and S_B^{USP14} (PDB 7W3I, F, right), with the same color scheme as used in Figure 3E and 3F. The reduced interleaving between RPT4 (D) or RPT6 (F) and the neighboring RPT subunits is highlighted as black dashed lines. **(E)** Superimposed structures of $26S^{TXNL1\text{-}OPEN}$ and PS_{RPT5} (PDB 9E8G), with the TXNL1 PITH domain, RPN10 VWA domain, and RPT4:RPT5 coiled coil highlighted in pink ($26S^{TXNL1\text{-}OPEN}$) and green (PS_{RPT5}). The shift or rotation of these regions in $26S^{TXNL1\text{-}OPEN}$ against PS_{RPT5} is indicated by black arrows. **(G)** Superimposed structures of $26S^{TXNL1\text{-}CLOSED}$ and USP14-bound 26S proteasome S_B^{USP14} (PDB 7W3I) with $TXNL1^{PITH}$ and USP14 colored in blue and pink, respectively. The other subunits are colored in light grey ($26S^{TXNL1\text{-}CLOSED}$) and dark grey ($S_B^{USP14}$). **(H)** Overlaid ^1H-^{15}N HSQC spectra of reduced ^{15}N-$RPN13^{Pru}$ (22 μM, redu, pink), oxidized ^{15}N-$RPN13^{Pru}$ (22 μM, oxi, black) and oxidized ^{15}N-$RPN13^{Pru}$ with 4-fold molar excess of $TXNL1^{Trx}$ (88 μM, blue). Enlarged regions for residues M31, C88, and F98, highlighted in black boxes, are shown in Figure 4F. **(I)** Overlaid ^1H-^{15}N HSQC spectra of reduced (redu) ^{15}N-$RPN13^{DEUBAD}$ (25 μM, rose-pink), oxidized (oxi) ^{15}N- $RPN13^{DEUBAD}$ (25 μM, black) and oxidized $RPN13^{DEUBAD}$ with 4-fold molar excess of $TXNL1^{Trx}$ (100 μM, cyan blue). Enlarged region for residues G353 and G360, highlighted in black boxes, are shown in Figure 4G. All spectra were acquired at 600 MHz and 10 °C with NMR buffer containing 20 mM sodium phosphate and 50 mM NaCl (pH 6.0). In addition, 1 mM DTT was added for samples under reducing condition. **(J)** Left panel: 156 μM RPN2 peptide (940-953) in reducing ITC buffer (20 mM sodium phosphate, 50 mM NaCl, 10 mM βME, pH 6.0) was injected into a calorimeter cell containing reduced 15.6 μM $RPN13^{Pru}$. Middle panel: 205.7 μM RPN2 peptide in non-reducing ITC buffer (20 mM sodium phosphate, 50 mM NaCl, pH 6.0) was injected into a calorimeter cell containing oxidized 20.57 μM $RPN13^{Pru}$. Right panel: 160 μM RPN2 peptide in non-reducing ITC buffer was injected into a calorimeter cell containing 16 μM oxidized $RPN13^{Pru}$ pre-incubated with 3-fold molar

excess of TXNL1^{Trx} and separated by size exclusion chromatography. All binding isotherms (top) were integrated to yield the change in enthalpy as a function of RPN2 peptide addition. The data were fit to a “One Set of Sites” binding model with the indicated thermodynamic values (bottom). All ITC experiment were performed at 25 °C.

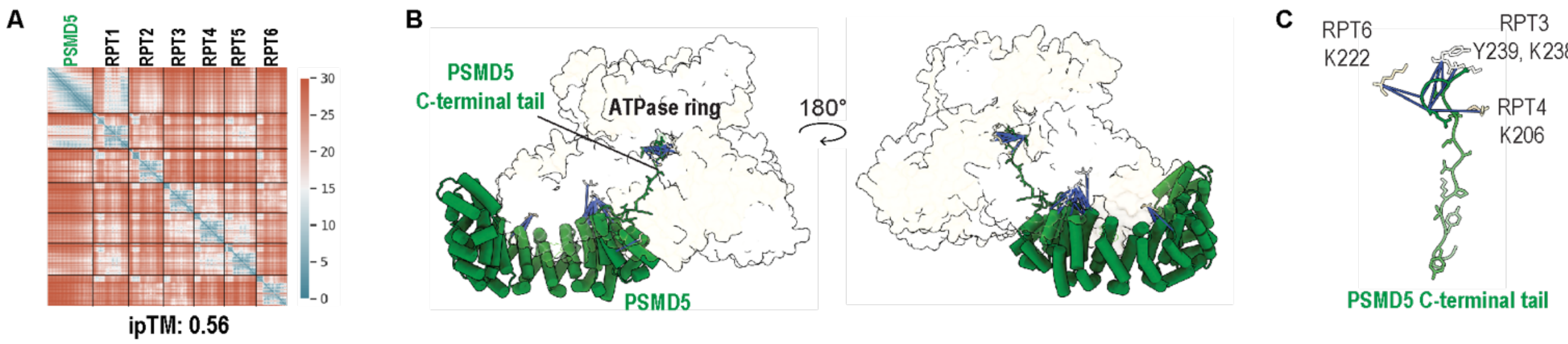

Figure S7. AlphaFold predicts a confidently scored model of PSMD5 bound to the proteasome ATPase ring, related to Figure 5. (A) Predicted Alignment Error (PAE) plot of a selected AlphaFold prediction. **(B)** AlphaFold3 predicted model of PSMD5 (green, cartoon view) bound to the ATPase ring (white, surface rendering) with crosslinks displayed by blue lines. **(C)** Expanded view an AlphaFold3 model centered on the PSMD5 C-terminal tail (green) to display its crosslinks (blue lines) with RPT6, RPT3, and RPT4. The sidechain of residues K222 (RPT6), K238 (RPT3), Y239 (RPT3), and K206 (RPT4) are displayed.

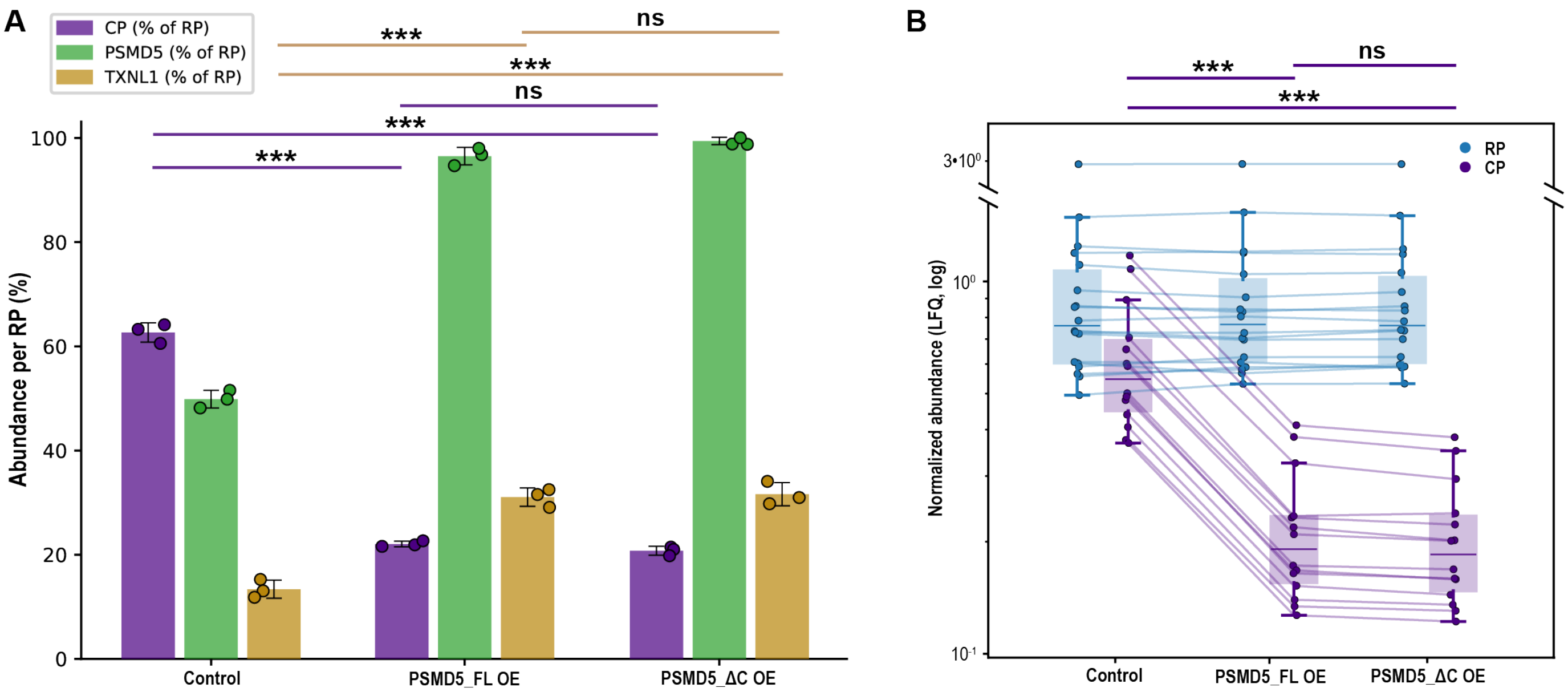

Figure S8. Over-expression of PSMD5 or PSMD5ΔC impairs RP interaction with the CP, related to Figure 5. (A) Percentage abundance of CP subunits (purple), PSMD5 (green), and TXNL1 (beige) in RPN1 pulldown experiments without or with over-expression of HA-PSMD5 (PSMD5 OE) or HA-PSMD5ΔC (PSMD5ΔC OE). The data were normalized to the mean RP subunit MaxLFQ intensity within each replicate. Bars represent the mean ± SD of three independent biological replicates. **(B)** Distributions of the protein intensity of individual RP (blue) and CP (purple) subunits in RPN1 pulldown experiments under control, HA-PSMD5, or HA-PSMD5ΔC conditions. Pulldown were performed with three biological replicates. Each dot represents the mean normalized abundance of an individual proteasome subunit across three biological replicates. Lines connect the same subunit across conditions. Box plots show the median and interquartile range (IQR), and whiskers extend to 1.5 × IQR. Statistical significance asterisks indicate two-sample Student's t-test; p-values; * p<0.05, ** p<0.01, *** p<0.001, ns p =/> 0.05. OE, over-expression; ns, not significant.

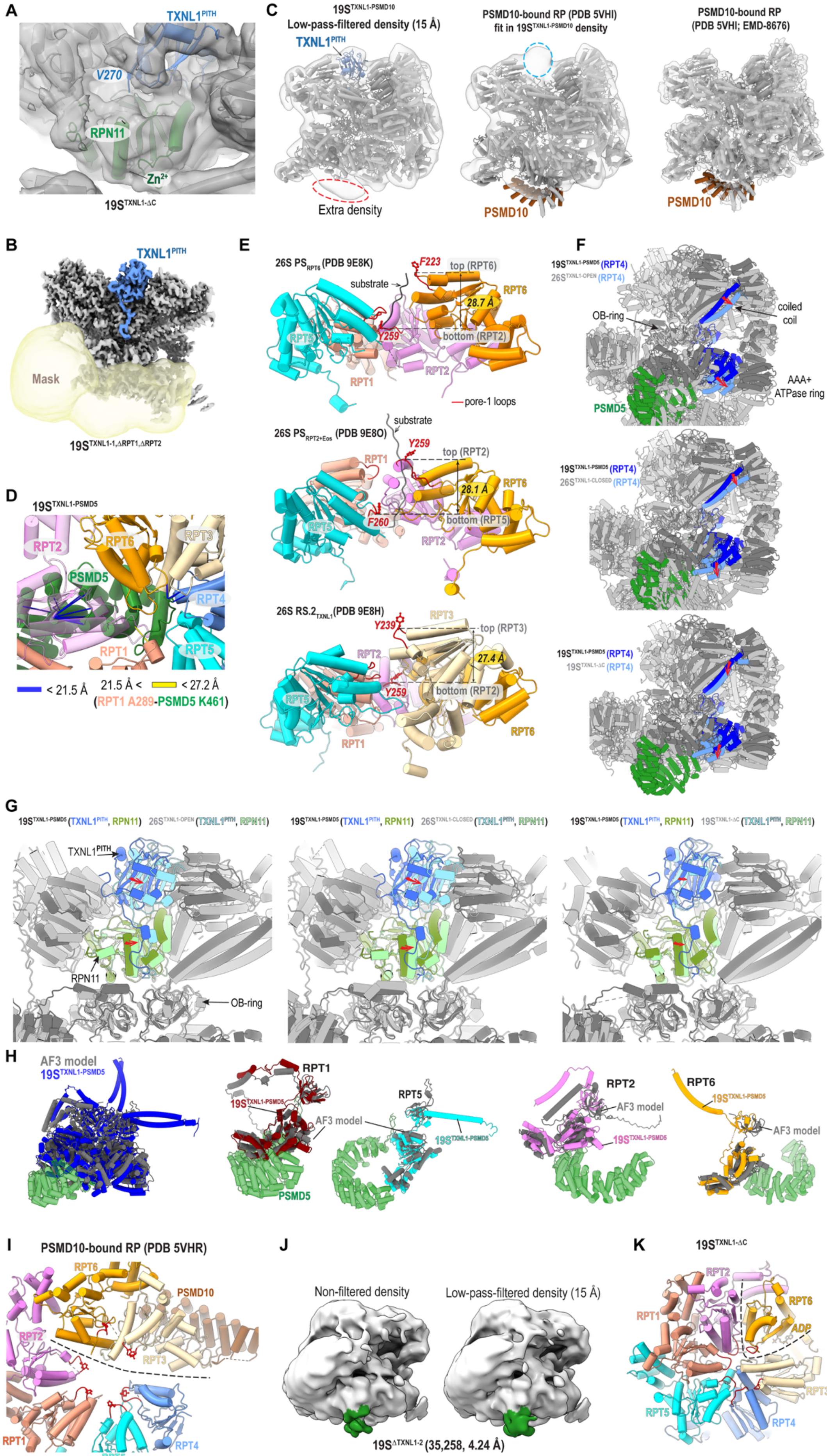
A
TXNL1^PITH
V270
RPN11
Zn2+
19S^TXNL1-ΔC
B
TXNL1^PITH
Mask
19S^TXNL1-1,ΔRPT1,ΔRPT2
C
19S^TXNL1-PSMD10
Low-pass-filtered density (15 Å)
TXNL1^PITH
Extra density
PSMD10-bound RP (PDB 5VHI) fit in 19S^TXNL1-PSMD10 density
PSMD10
PSMD10-bound RP (PDB 5VHI; EMD-8676)
PSMD10
D
19S^TXNL1-PSMD5
RPT2
RPT6
RPT3
PSMD5
RPT4
RPT1
RPT5
< 21.5 Å
21.5 Å <
< 27.2 Å
(RPT1 A289-PSMD5 K461)
E
26S PS_RPT6 (PDB 9E8K)
F223
top (RPT6)
substrate
RPT6
28.7 Å
Y259
bottom (RPT2)
RPT5
RPT1
RPT2
pore-1 loops
substrate
26S PS_RPT2+Eos (PDB 9E8O)
Y259
RPT1
top (RPT2)
RPT6
28.1 Å
F260
bottom (RPT5)
RPT5
RPT2
26S RS.2_TXNL1 (PDB 9E8H)
Y239
RPT3
top (RPT3)
RPT2
27.4 Å
Y259
bottom (RPT2)
RPT5
RPT1
RPT6
F
19S^TXNL1-PSMD5 (RPT4)
26S^TXNL1-OPEN (RPT4)
coiled coil
OB-ring
AAA+ ATPase ring
PSMD5
19S^TXNL1-PSMD5 (RPT4)
26S^TXNL1-CLOSED (RPT4)
19S^TXNL1-PSMD5 (RPT4)
19S^TXNL1-ΔC (RPT4)
G
19S^TXNL1-PSMD5 (TXNL1^PITH, RPN11) 26S^TXNL1-OPEN (TXNL1^PITH, RPN11)
19S^TXNL1-PSMD5 (TXNL1^PITH, RPN11) 26S^TXNL1-CLOSED (TXNL1^PITH, RPN11)
19S^TXNL1-PSMD5 (TXNL1^PITH, RPN11) 19S^TXNL1-ΔC (TXNL1^PITH, RPN11)
TXNL1^PITH
RPN11
OB-ring
H
AF3 model
19S^TXNL1-PSMD5
RPT1
19S^TXNL1-PSMD5
AF3 model
PSMD5
RPT5
19S^TXNL1-PSMD5
RPT2
AF3 model
19S^TXNL1-PSMD5
RPT6
19S^TXNL1-PSMD5
AF3 model
I
PSMD10-bound RP (PDB 5VHR)
RPT6
PSMD10
RPT2
RPT3
RPT1
RPT5
RPT4
J
Non-filtered density
Low-pass-filtered density (15 Å)
19S^ΔTXNL1-2 (35,258, 4.24 Å)
K
19S^TXNL1-ΔC
RPT2
RPT6
RPT1
ADP
RPT3
RPT5
RPT4

Figure S9. PSMD5 inserts its C-terminus into the AAA+ ATPase ring flattening the distance between its top-most and bottom-most pore-1 loops, related to Figure 6. **(A)** Expanded view of the $19S^{TXNL1\text{-}\Delta C}$ structure to illustrate that the 20 amino acids after V270 in the TXNL1 C-terminal tail (blue) is not detected, nor interacting with the RPN11 Zn^{2+} ion (green). The $19S^{TXNL1\text{-}\Delta C}$ density map is displayed in grey. **(B)** A mask (yellow map) focused on the RPT1-RPT2-RPT5-RPT6 AAA+ domain and RPN1 was created and used for 3D classification, 3D variability analyses and local refinement of $19S^{TXNL1\text{-}1,\Delta RPT1,\ \Delta RPT2}$. **(C)** Density map (grey) for $19S^{TXNL1\text{-}PSMD10}$ with fitted cartoon model (left; TXNL1 PITH in blue and extra density highlighted by a red circle), or PSMD10-bound RP structure (middle; PDB 5VHI, PSMD10 in brown and the region lacking TXNL1 density highlighted by a blue circle). The density map of the PSMD10-bound RP (EMD-8676) with fitted structure (PDB 5VHI) is shown in right panel. (**D**) Expanded view of $19S^{TXNL1\text{-}PSMD5}$ showing the crosslinks between PSMD5 (green) and RPT subunits, represented as lines colored blue for distances <21.5 Å and yellow for one between 21.5 and 27.2 Å. **(E)** Expanded views of the ATPase large AAA+ subdomains in 26S PS_{RPT6} (PDB 9E8K, top), $PS_{RPT2+Eos}$ (PDB 9E8O, middle), and $RS.2_{TXNL1}$ (PDB 9E8H, bottom). RPT subunits are displayed and colored as in Figure 6F. **(F and G)** Superimposed cartoon structures of $19S^{TXNL1\text{-}PSMD5}$ (dark grey) with $26S^{TXNL1\text{-}OPEN}$ (grey, top in F and left in G), $26S^{TXNL1\text{-}CLOSED}$ (grey, middle in F and G), and $19S^{TXNL1\text{-}\Delta C}$ (grey, bottom in F and right in G), aligned by the OB ring. In F, RPT4 and PSMD5 in $19S^{TXNL1\text{-}PSMD5}$ are colored dark blue and green, respectively. RPT4 in $26S^{TXNL1\text{-}OPEN}$, $26S^{TXNL1\text{-}CLOSED}$, and $19S^{TXNL1\text{-}\Delta C}$ is colored light blue. In G, the TXNL1 PITH domain and RPN11 are colored blue and green, respectively, which darker coloring in $19S^{TXNL1\text{-}PSMD5}$ and lighter coloring shades in $26S^{TXNL1\text{-}OPEN}$, $26S^{TXNL1\text{-}CLOSED}$, and $19S^{TXNL1\text{-}\Delta C}$. Black arrows indicate the positional shifts of proteasome subunits upon PSMD5 binding. For clarity, the TXNL1 Trx domain of $26S^{TXNL1\text{-}OPEN}$ was omitted in the left panel of G. **(H)** The ATPase ring and PSMD5 from the $19S^{TXNL1\text{-}PSMD5}$ (blue) and the AlphaFold3 model (grey) are aligned based on PSMD5 (left). Expanded views highlight the alignment of RPT1, RPT2, RPT5, and RPT6. The RPT subunits in $19S^{TXNL1\text{-}PSMD5}$ are colored according to the scheme used in Figure 6F, whereas the corresponding RPT subunits in the AlphaFold3 model are shown in dark grey. PSMD5 is colored green. **(I)** Top view of the ATPase large AAA+ subdomains in PSMD10-bound RP (PDB 5VHR). The color scheme is as in Figure 6G. The reduced interleaving between RPT3/RPT6 and their neighboring RPT subunits is highlighted by a black dashed line. **(J)** Extra density (green) is detected in the non-filtered (left) and low-pass-filtered (15 Å, right) $19S^{\Delta TXNL1\text{-}2}$ map near the ATPase ring. **(K)** Top views of the ATPase large AAA+ subdomains in $19S^{TXNL1\text{-}\Delta C}$, with a black dashed line highlighting the seam position of RPT6.
